# Screening of phenolic components and antimicrobial properties of *Iris persica* L. subsp. *persica* extracts by in vitro and in silico methods

**DOI:** 10.1002/fsn3.4251

**Published:** 2024-06-24

**Authors:** Tuba Unver, Harun Uslu, Ismet Gurhan, Bunyamin Goktas

**Affiliations:** ^1^ Department of Pharmaceutical Microbiology, Faculty of Pharmacy Inonu University Malatya Turkey; ^2^ Department of Pharmaceutical Chemistry, Faculty of Pharmacy Fırat University Elazığ Turkey; ^3^ Department of Pharmaceutical Botany, Faculty of Pharmacy Inonu University Malatya Turkey

**Keywords:** antimicrobial activity, herbal medicine, *Iris persica*, molecular docking, phenolic contents

## Abstract

The tendency toward natural herbal products has increased due to the antibiotic resistance developed by microorganisms and the severe side effects of antibiotics commonly used in infectious diseases worldwide. Although antimicrobial studies have been conducted with several species of the *Iris* genus, this study is the first in the literature to be performed with *Iris persica* L. subsp. *persica* aqueous and methanol extracts. In this study, the phenolic content of *I. persica* was determined by LC–MS/MS analysis, the in vitro antimicrobial activity of *I. persica* aqueous and methanol extracts was examined, and this study was supported by in silico analysis. Consequently, methanol and aqueous extracts were observed to have inhibitory effects against all tested microorganisms except *Candida krusei*. Although the MIC values of aqueous extract and methanol extract against *Staphylococcus aureus* and *Klebsiella pneumoniae* are the same (22.5 and 11.25 mg/mL, respectively), the inhibitory effect of aqueous extract is generally more potent (MIC value is 11.25 mg/mL for *Candida parapsilosis* and other bacterial species, and 90 mg/mL for *Candida albicans* and *Candida tropicalis*) than that of methanol extract. In silico results showed that hydroxybenzaldeyde, vanillin, resveratrol, isoquercitrin, kaempferol‐3‐glucoside, fisetin, and luteolin were more prone to antifungal activity. Hence, shikimic, gallic, protocatechuic, vanillic, caffeic, *o*‐coumaric, *trans*‐ferulic, sinapic acids, and hesperidin were more prone to antibacterial activity. In vitro and in silico results show that the antibacterial activity of our extracts may be higher than the antifungal activity. This preliminary study indicates the anti‐infective potential of *I. persica* extracts and their usability in medicine and pharmacology.

## Graphical abstract

This study, conducted in silico and in vitro, explored the antimicrobial properties of *Iris persica*. *I. persica* aqueous and methanol extracts were analyzed by LC–MS/MS, revealing shikimic acid and vanillic acid as the most dominant compounds. *I. persica* extracts possess antimicrobial properties against tested microorganisms (MICs ranged between 11.25 and 180 mg/mL), with the exception of *Candida krusei*. This research suggests that *I. persica* could be a valuable natural protectant to control infections, offering potential in medicine and pharmaceutical science.

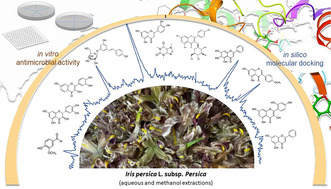

## 1 INTRODUCTION

Infectious diseases constitute a large portion of total pathologies, and fighting infectious diseases worldwide has required severe effort, time, and economic power for centuries (CDC, 2021). More than 100 different types of chemotherapeutic antibiotics have been discovered today (Nigam et al., 2014). Antibiotics can cure infectious diseases by acting on microorganisms through various mechanisms (Reygaert, 2018). However, the development of resistance in microorganisms to antibiotics of synthetic origin has caused serious problems in treatment (Costelloe et al., 2010; Dong et al., 2007; Livermore, 2004a). An example is *Staphylococcus aureus*, which has developed resistance to methicillin and fluoroquinolone (Kaatz, 2005). Thus, antibiotic resistance reduces treatment success, causes serious global health problems, and poses a serious economic burden to the healthcare sector worldwide (Berger‐Bächi, 2002; Levy, 2001; Livermore, 2004b). Therefore, researchers are turning to structural changes in existing antibiotics and developing natural alternative antimicrobial products (Bilal et al., 2023; Costelloe et al., 2010). Herbal medicines are in demand today because they play an essential role in developing powerful therapeutic agents, and their popularity as natural alternative antimicrobial products is increasing daily. Approximately 80% of people in developing countries use traditional medicine based on plant species for general health care (Hussain et al., 2021). Herbal medicines treat diseases or injuries using whole plants or parts of plants (Gossell‐Williams et al., 2006). Herbal medicines are the oldest health care known to man and are used to prevent disease, support treatment, or as a purely therapeutic agent (De Smet, 1997). The World Health Organization (WHO) has potent ingredients derived from plants and different herbal medicines as labeled medicinal products. WHO has established strict guidelines for evaluating the quality, safety, and effectiveness of herbal medicines (WHO, 1996). Since herbs are natural resources, they are generally considered safe and have advantages such as easy accessibility, low cost, and high potency in some plant species (Abhishek et al., 2006). At the same time, herbal medicines, unlike allopathic medicines, have fewer side effects and are used naturally (Naja et al., 2015; WHO, 2002).

The *Iris* genus, which includes primarily species of medical importance, belongs to the Iridaceae family, which includes more than 300 species (Hussain et al., 2021; Ibrahim et al., 2012). Plants of the genus *Iris* are medicinal plants and have been proven to have anticancer, antioxidant, and antituberculosis effects (Kaššák, 2012; Kukuła‐Koch et al., 2015). They are widely distributed worldwide, growing wild, mainly in Europe, Africa, Asia, North America, and Turkey (Desam & Al‐Rajab, 2021; Hussain et al., 2021; Rigano et al., 2009; Venditti et al., 2017). Most *Iris* species are found in desert, semi‐desert, or dry, rocky habitats and are attractive for their beauty and pleasant scent (Basser et al., 2011). *Iris* is a valuable medicinal and aromatic plant, so it is also grown as a cultivated plant (Hussain et al., 2021). So far, various antimicrobial studies have been carried out with a few species belonging to the *Iris* genus, but molecular docking studies still need to be added to the literature (Basgedik et al., 2014; Bilal et al., 2023; Kovalev et al., 2017; Yousefsani et al., 2021). Another antifungal study was conducted with essential oil obtained from *Iris persica* (Amin et al., 2017). This is the first study in the literature conducted with *I. persica* aqueous and methanol extracts. In this study, aqueous and methanol extracts of *I. persica*, collected primarily from the mountains of Malatya city in Turkey, were obtained under appropriate conditions. Later, the antimicrobial activity of these extracts was proven using agar dilution and broth dilution methods. The results were detailed with in silico molecular docking studies.

## 2 MATERIAL METHODS

### 2.1 Plant collection and preparation of extracts

**FIGURE 1:**
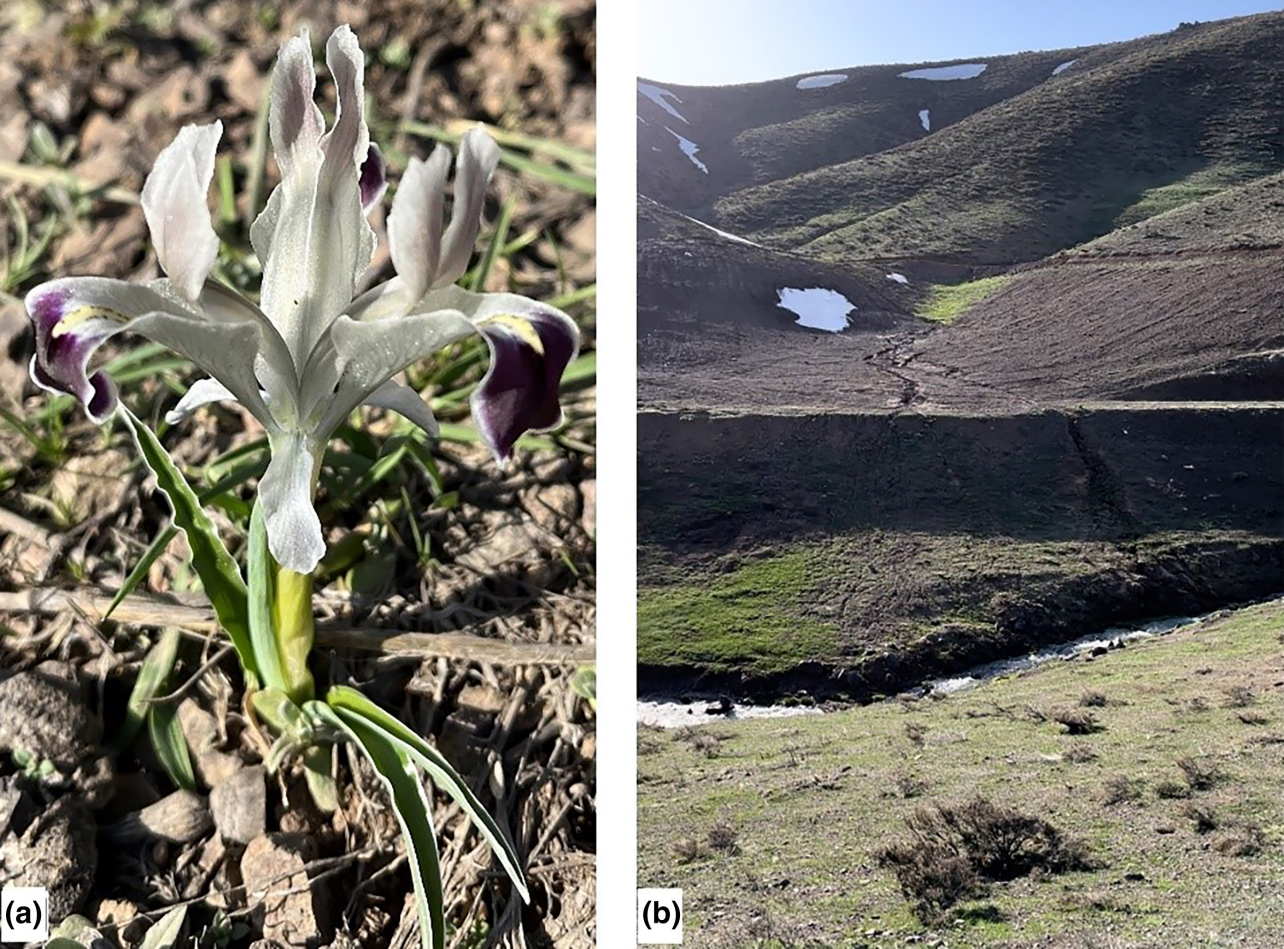
(a) *Iris persica* L. subsp. *persica*. (b) The nature where *I. persica* grows and is collected (Kubbe Mountains, Puturge‐Malatya, Turkey).

The plant *I. persica* L. subsp. *persica* was collected in March 2023 from the Kubbe Mountain of the Puturge region, Malatya, Turkey (Figure 1). It was brought to the Pharmaceutical Botany Laboratory of the Faculty of Pharmacy (Inonu University) (Voucher Specimen Code: TU1003) and identified by Prof. Dr. Adil Güner (Güner, 2021). Aerial parts of the plant (stem, leaf, and flower) were dried and then crushed with a grinder. For extraction, water and methanol were used. 100 mL of water and methanol were added separately to the 10 g of plant aerial parts and left for maceration at room temperature of 15–20°C. This protocol was replicated three times by adding solvent and removing the filtrate. The filtrates were collected and evaporated in the rotary evaporator (Heidolph Laborota 4000, Germany) till the solvents were removed (Radulović et al., 2007). The obtained extracts were stored in the refrigerator at −19°C for chemical analyses and antimicrobial activity tests.

### 2.2 LC–MS/MS analysis

Samples were taken at 50 mg and dissolved in 1 mL methanol. The latter was centrifuged at 7690 g for 10 min. Then, 100 μL was taken from the methanol phase and diluted with 9000 μL (450 water/450 methanol). Afterward, LC–MS/MS analysis was performed after filtering with a 0.25 filter. LC–MS/MS studies were conducted using an Electrospray Ionization Source and a Reversed Phase C18 column (Poroshell 120 SB‐C18 (3.0 × 100 mm, I.D., 2.7 μm)) on an Agilent 6460 Triple Quad LC–MS/MS with a 1290 Infinity UPLC system. The mobile phases A and B comprised 1% formic acid in water and 1% in acetonitrile, respectively. Applying gradient elution, the column temperature was set to 30°C, and the mobile phase flow rate was set to 0.4 mL/min. The retention times of the compounds were defined by comparing them with reference HRMS data obtained from the Eastern Anatolia High Technology Application and Research Center. The mobile phase (1 mL; A: B; 50:50; v/v) was dissolved with 1 mg of dry aqueous and methanol extract. Before being injected into the LC in a volume of 5 μL, the solution was filtered through a 0.45‐m filter.

### 2.3 Bacterial and fungal strains and growth conditions

The inhibitory effects of *I. persica* extracts were tested against two Gram‐positive and three Gram‐negative bacteria and four yeast strains. *Staphylococcus aureus* ATCC (American Type Culture Collection) 12600, *Enterobacter aerogenes* ATCC 51697, *Pseudomonas aeruginosa* ATCC 10145, *Klebsiella pneumoniae* ATCC 13883, *Escherichia coli* NEB (New England Biolabs) C2987, C*andida albicans* ATCC 14053, *Candida tropicalis* ATCC 13803, *Candida parapsilosis* ATCC 22019, and *Candida krusei* ATCC 14243 were used in this study. Bacterial strains were subcultured on Muller Hinton agar plates, and Muller Hinton agar and broth were used for antibacterial activity determination. Saboraud dextrose agar and broth were used for *Candida* species subculturing and antimicrobial assays.

### 2.4 Antibacterial and antifungal activities of the extracts

The minimum inhibitory concentrations (MIC) of the methanol and aqueous extracts were determined using the agar dilution and broth microdilution methods. In the agar dilution method adapted from the Clinical and Laboratory Standards Institute (CLSI), as a first step, 2.160 g of *I. persica* methanol and aqueous extracts were added to 6 mL of agar medium separately. Then, a two‐fold dilution was carried out (CLSI, 2005). Consequently, the concentration of plant extracts in the plates ranged from 180 to 0.176 mg/mL. Pure Muller Hinton agar for bacterial strains and Sabouraud dextrose agar for yeast strains were prepared separately without the extracts for control plates. After that, Muller Hinton agar plates were divided into five sections for bacterial strains. *S. aureus*, *E. aerogenes*, *P. aeruginosa*, *E. coli*, and *K. pneumoniae* were inoculated onto sections 1, 2, 3, 4, and 5, respectively. Furthermore, Sabouraud dextrose agar plates were divided into four sections. *C. albicans*, *C. krusei*, *C. tropicalis*, and *C. parapsilosis* were inoculated onto sections 1, 2, 3, and 4 of agar plates, respectively, as applied before (Unver et al., 2023; Unver & Gurhan, 2024). The final inoculum sizes were 1.5 × 10^8^ colony‐forming units CFU/mL for bacteria and 1–1.5 × 10^6^ CFU/mL for *Candida* in every plate. One microliter of bacteria and yeast suspension was spread onto agar plates, which included different concentrations of the extracts. Subsequently, the plates were incubated at 36°C for 18–24 h. The next day, the growth of bacterial and fungal species on the plates was evaluated, and MIC values were determined. The experiment was repeated three times with the same protocol.

In the broth dilution method, a 96‐well microplate was used, and the CLSI standard methodology was carried out with minor modifications (CLSI, 2018). 200 μL of Muller Hinton broth (for bacterial strains) or Sabouraud dextrose broth (for fungal strains) was placed into the microplate wells. Then, 72 mg of plant extract was transferred to 200 μL of Muller Hinton broth in the first well to yield a 180 mg/mL concentration. After twofold serial dilution of the extracts, 180, 90, 45, 22.5, 11.25, 5.625, 2.813, 1.406, 0.703, and 0.352 mg/mL extract concentrations were obtained from wells 1 to 10. Subsequently, a direct colony suspension for each strain was made in distilled water, and their concentration was set to 1.5 × 10^8^ CFU/mL for bacteria and 1–1.5 × 10^6^ CFU/mL for yeast strains. After that, 1 μL inoculations of *S. aureus*, *E. aerogenes*, *P. aeruginosa*, *E. coli*, *K. pneumoniae*, *C. albicans*, *C. krusei*, *C. tropicalis*, and *C. parapsilosis* in the A, B, C, D, E, F, G, H, and I rows were prepared, respectively. Microorganisms used in Muller Hinton broth and Saboraud dextrose broth were placed in the 11th well for positive control. Pure Muller Hinton broth and saboraud dextrose broth without extracts were placed in the 12th well for negative controls. The microplate with different extracts of *I. persica* concentrations was incubated at 36°C for 18–24 h. The next day, 15 μL of the 0.15% resazurin (w/v) was added to each well in the microplate. It was incubated at 36°C for 4–5 h, and the color change was observed to determine the growth of the microorganism.

### 2.5 Molecular docking analysis

**FIGURE 2:**
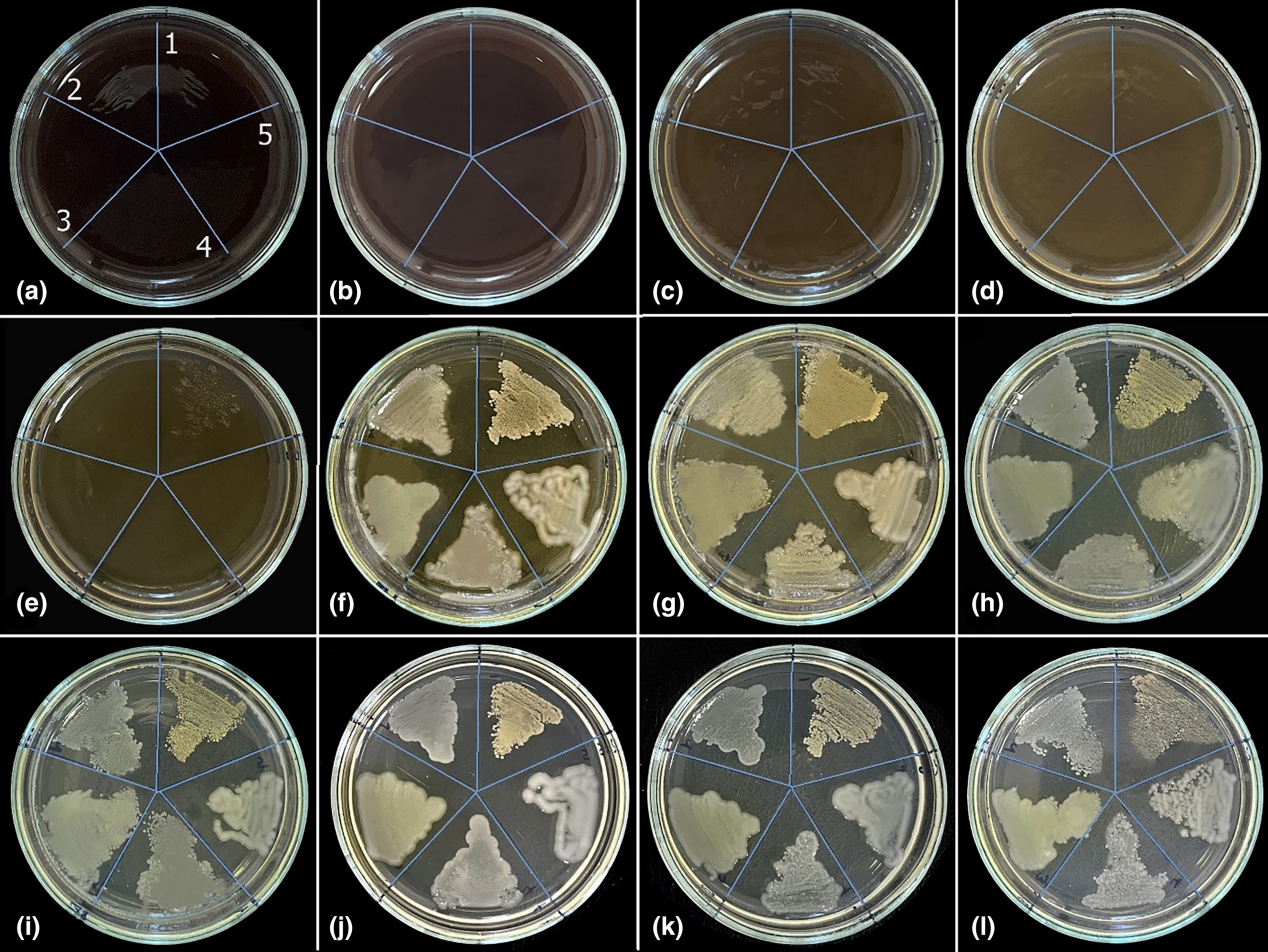
The pictures of the antibacterial activity assay of *I. persica* aqueous extract with different concentrations against (1) *S. aureus*, (2) *E. aerogenes*, (3) *P. aeruginosa*, (4) *E. coli*, and (5) *K. pneumoniae*. (a) 180, (b) 90, (c) 45, (d) 22.5, (e) 11.25, (f) 5.625, (g) 2.813, (h) 1.406, (i) 0.703, (j) 0.352, (k) 0.176 mg/mL *I. persica* aqueous extract, and (l) control.

**FIGURE 3:**
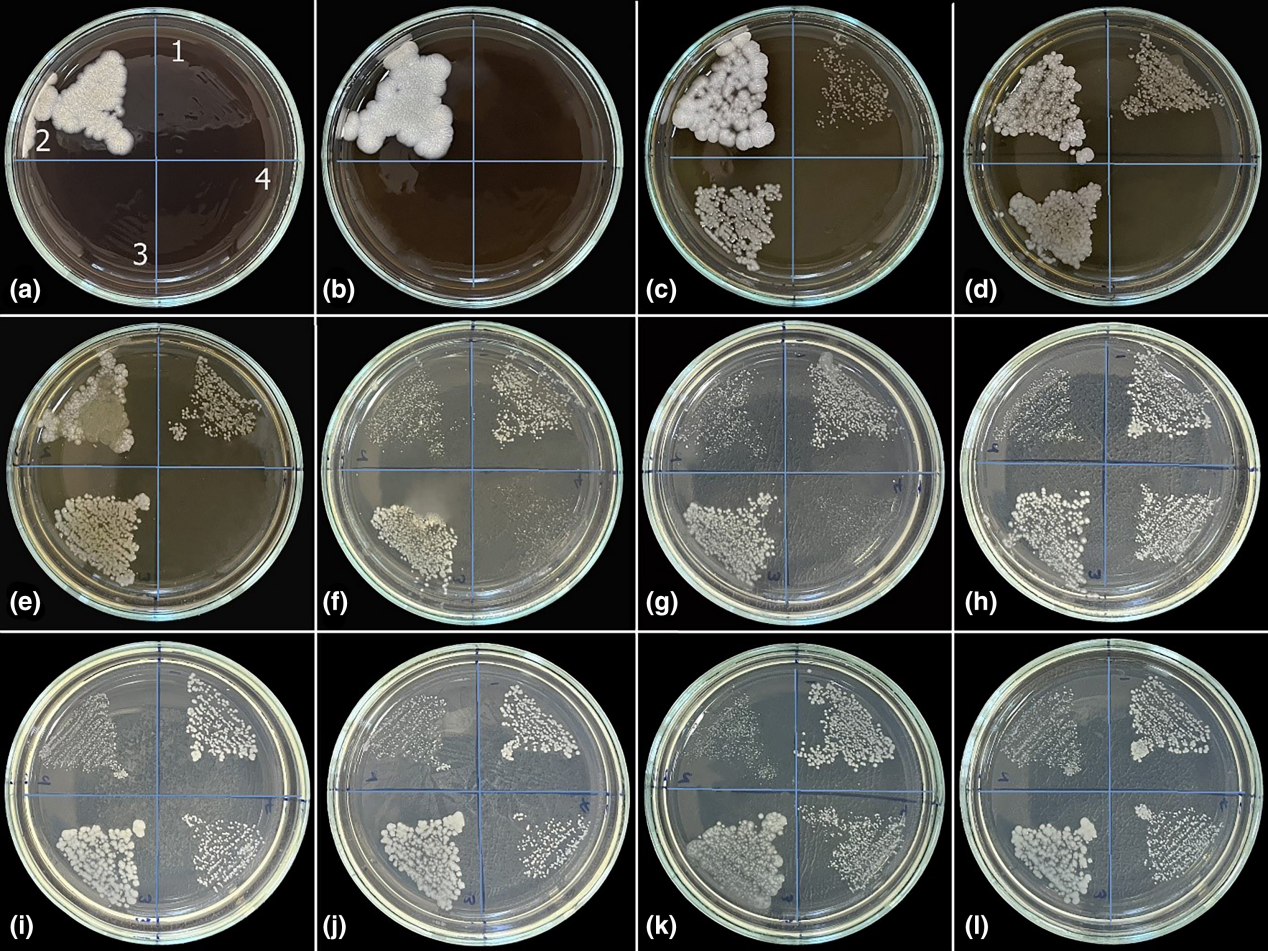
The pictures of the antifungal activity assay of *I. persica* aqueous extract with different concentrations against (1) *C. albicans*, (2) *C. krusei*, (3) *C. tropicalis*, and (4) *C. parapsilosis*. (a) 180, (b) 90, (c) 45, (d) 22.5, (e) 11.25, (f) 5.625, (g) 2.813, (h) 1.406, (i) 0.703, (j) 0.352, (k) 0.176 mg/mL *I. persica* aqueous extract, and (l) control.

**FIGURE 4:**
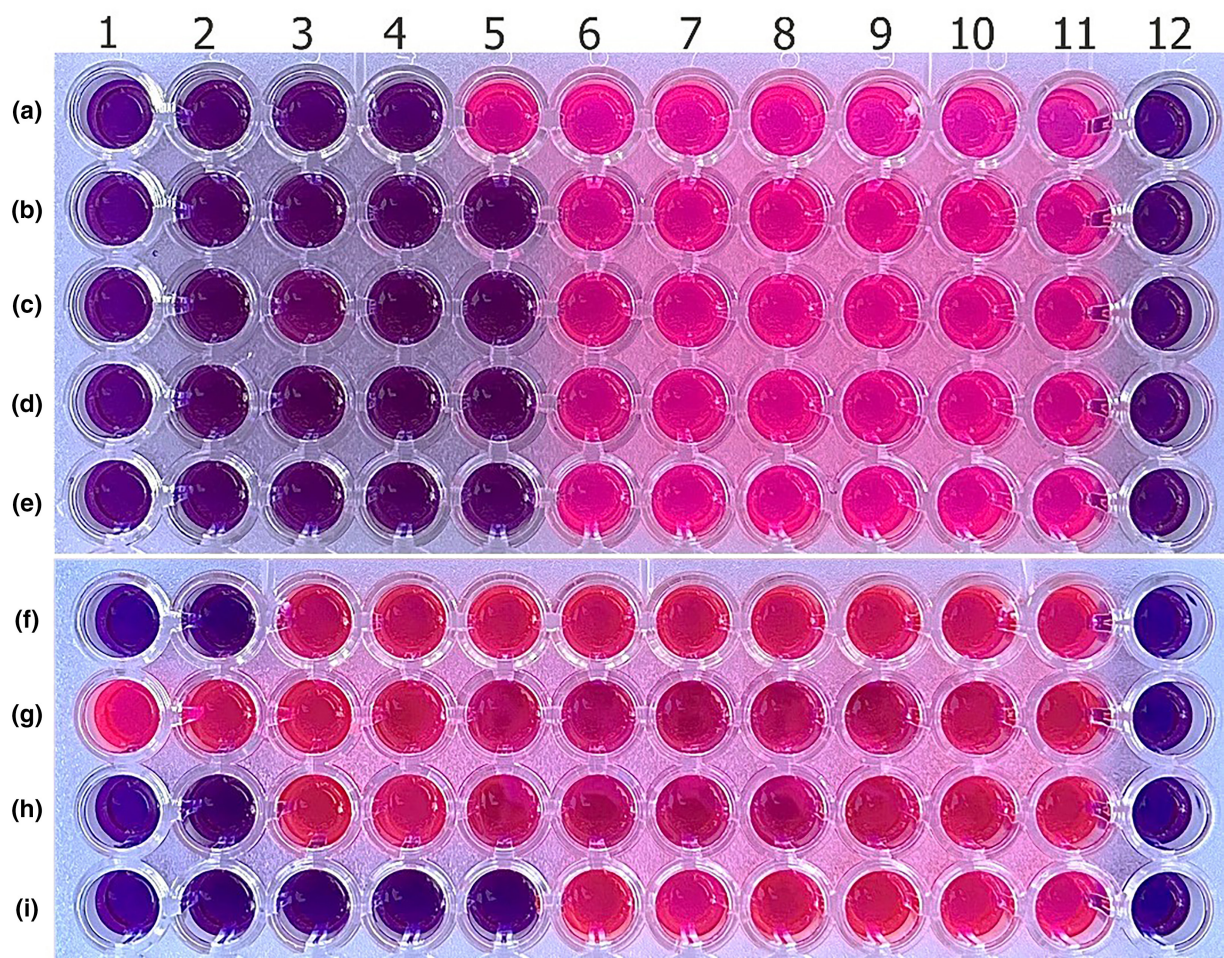
Microplate photo of *I. persica* aqueous extract antimicrobial activity assay against *S. aureus* (a), *E. aerogenes* (b), *P. aeruginosa* (c), *E. coli* (d), *K. pneumonia* (e), *C. albicans* (F), *C. krusei* (g), *C. tropicalis* (h), and *C. parapsilosis* (i). The aqueous extract concentrations in wells A4 (22.5 mg/mL), B5 (11.25 mg/mL), C5 (11.25 mg/mL), D5 (11.25 mg/mL), E5 (11.25 mg/mL), F2 (90 mg/mL), H2 (90 mg/mL), and I5 (11.5 mg/mL) without any color change were accepted as MIC values. 11. positive control, 12. negative control.

**FIGURE 5:**
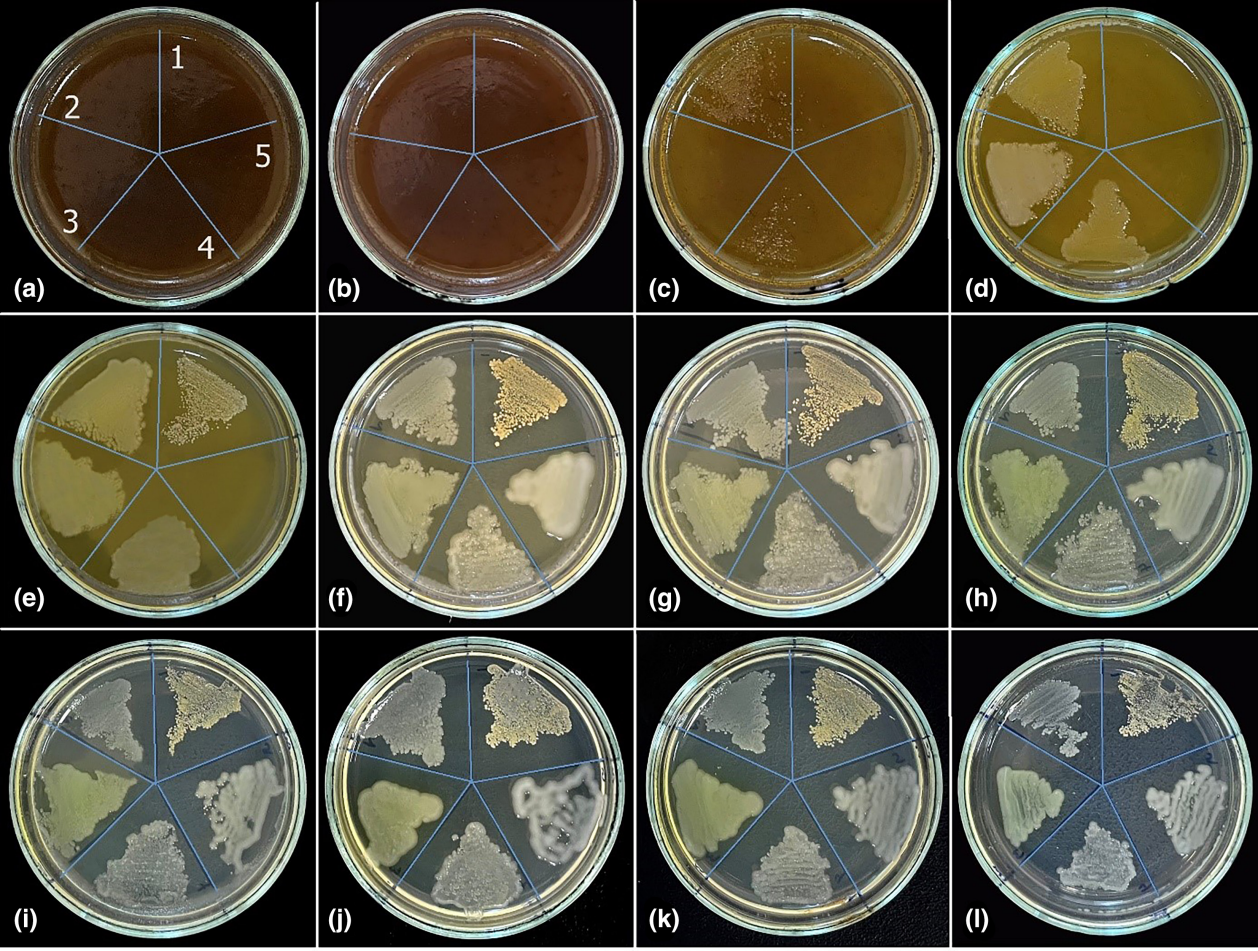
The pictures of the antibacterial activity assay of *I. persica* methanol extract with different concentrations against (1) *S. aureus*, (2) *E. aerogenes*, (3) *P. aeruginosa*, (4) *E. coli*, and (5) *K. pneumoniae*. (a) 180, (b) 90, (c) 45, (d) 22.5, (e) 11.25, (f) 5.625, (g) 2.813, (h) 1.406, (i) 0.703, (j) 0.352, (k) 0.176 mg/mL *I. persica* methanol extract, and (l) control.

**FIGURE 6:**
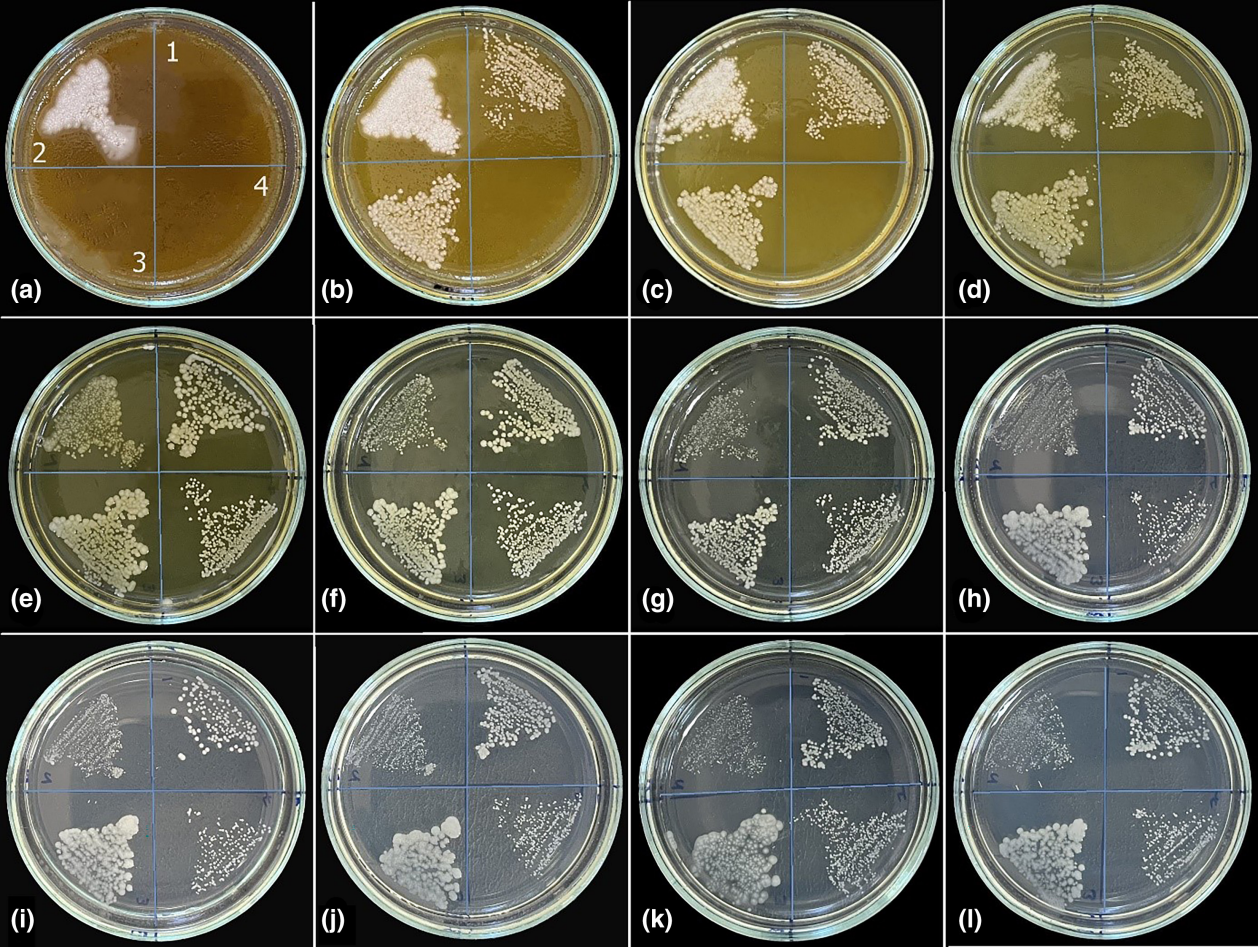
The pictures of the antifungal activity assay of *I. persica* methanol extract with different concentrations against (1) *C. albicans*, (2) *C. krusei*, (3) *C. tropicalis*, and (4) *C. parapsilosis*. (a) 180, (b) 90, (c) 45, (d) 22.5, (e) 11.25, (f) 5.625, (g) 2.813, (h) 1.406, (i) 0.703, (j) 0.352, (k) 0.176 mg/mL *I. persica* methanol extract, and (l) Control.

**FIGURE 7:**
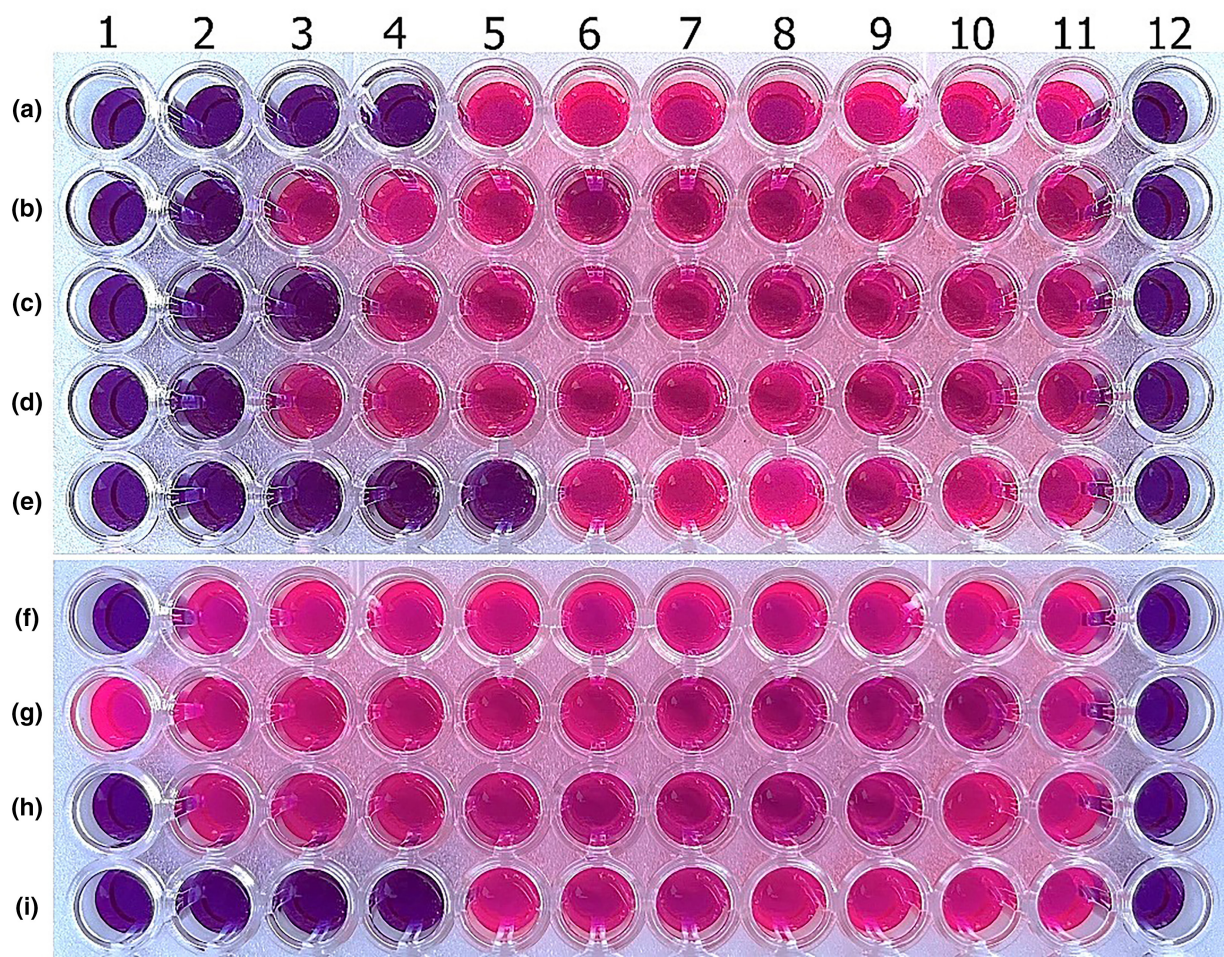
Microplate photo of *I. persica* methanol extract antimicrobial activity assay against *S. aureus* (a), *E. aerogenes* (b), *P. aeruginosa* (c), *E. coli* (d), *K. pneumonia* (e), *C. albicans* (f), *C. krusei* (g), *C. tropicalis* (h), and *C. parapsilosis* (i). The methanol extract concentration in wells A4 (22.5 mg/mL), B2 (90 mg/mL), C3 (45 mg/mL), D2 (90 mg/mL), E5 (11.25 mg/mL), F1 (180 mg/mL), H1 (180 mg/mL), and I4 (22.5 mg/mL) without any color change was accepted as MIC values. 11. positive control, 12. negative control.

**FIGURE 8:**
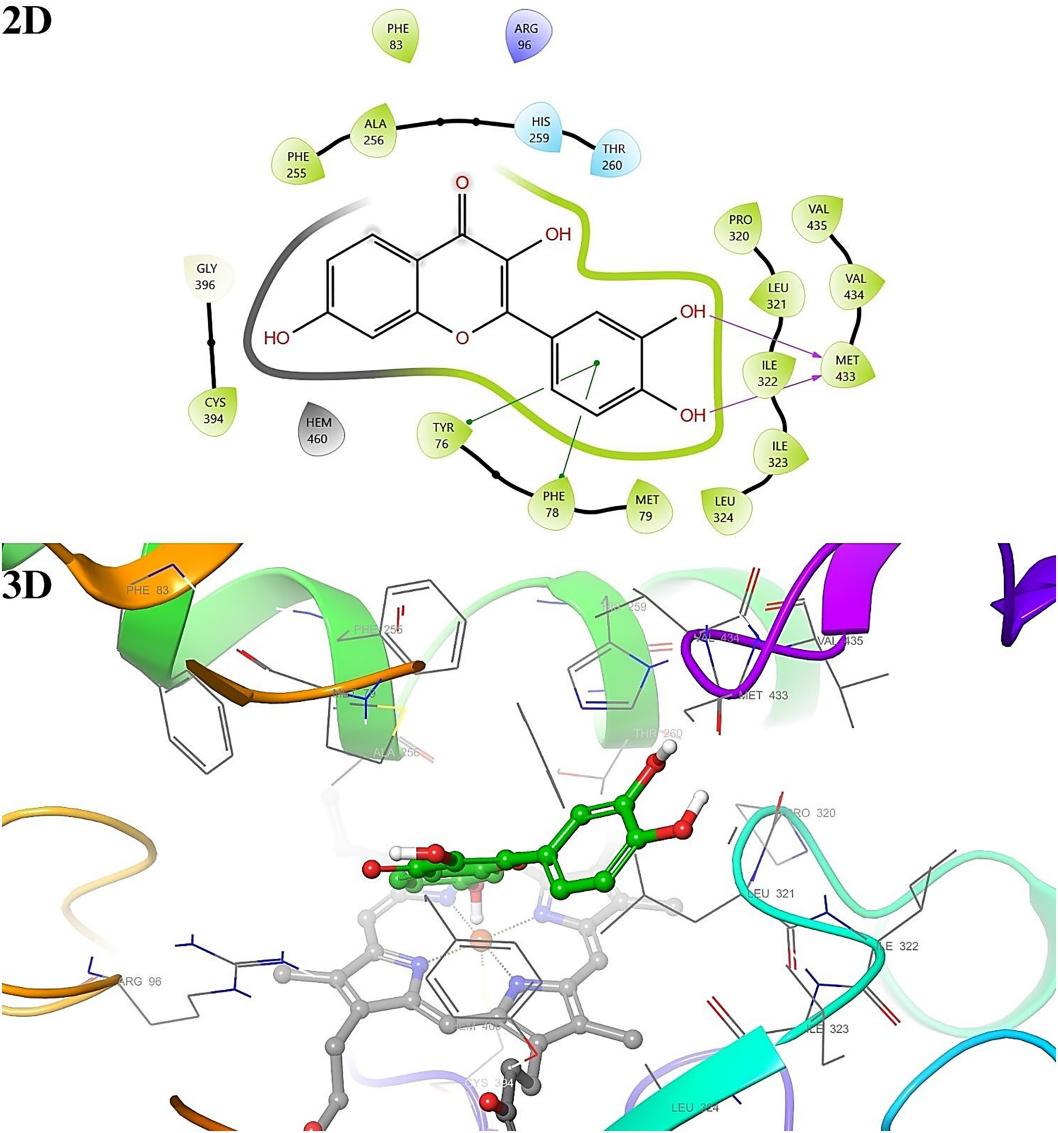
2D and 3D interaction diagram with 1EA1 for fisetin.

**FIGURE 9:**
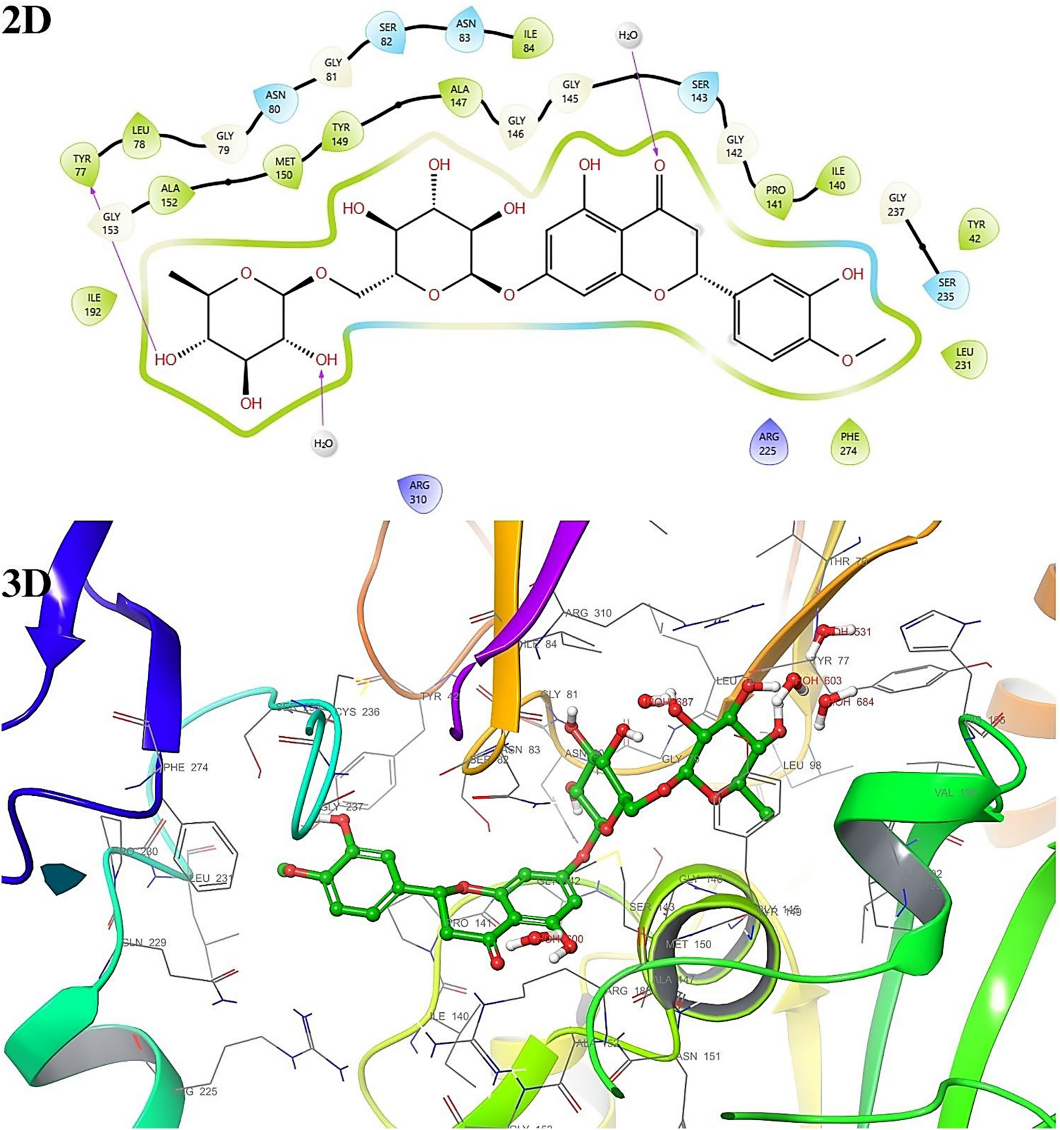
2D and 3D interaction diagram with 1HSK for hesperidin.

Molecular docking studies were performed using an in silico procedure to identify the binding modes of compounds detected in the extracts at the active sites of selected macromolecules to evaluate 1EA1‐antifungal activity (Podust et al., 2001) and 1HSK‐antimicrobial activity (Benson et al., 2001). The crystal structures of these macromolecules were retrieved from the Protein Data Bank server (RCSB PDB, 2023). Macromolecule structures were edited using the Schrödinger Maestro. Energy minimization of the ligands was carried out with the ChemOffice program. Afterward, the docking process was performed using standard settings with both Autodock (Sanner, 1999) and Vina (Trott & Olson, 2010) programs, and the results were presented in a table (Tables 4 and 5). To validate the docking program, some cocrystallized ligands were re‐docked on the target regions of macromolecules, and RMSD values were set to less than two (<2). The results were visualized using the Maestro program (Maestro, Schrödinger, LLC, New York, NY, 2023‐4; Figures 8 and 9 and Figures 1S–64S).

## 3 RESULTS

### 3.1 Chemical analysis of the extracts using LC–MS/MS

Plant extracts prepared with methanol and water were analyzed by LC–MS/MS, and their contents were determined. The compounds detected in the extracts were plotted to get SMILES from PubChem (https://pubchem.ncbi.nlm.nih.gov). Phenolic compounds and their chemical structures determined as a result of the analysis are given below (Table 1).

**TABLE 1:** Chemical structure of phenolic compounds found in methanol and aqueous extracts.

Compound	Chemical structure of compounds
Shikimic acid	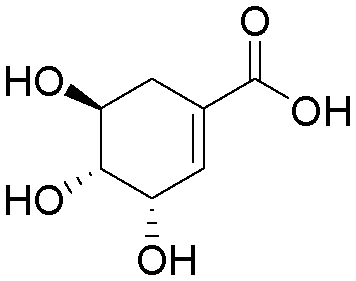
Gallic acid	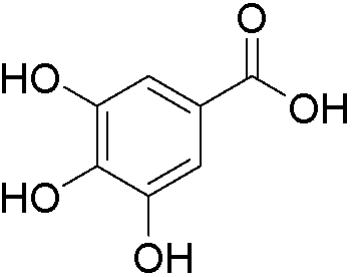
Protocatechuic acid	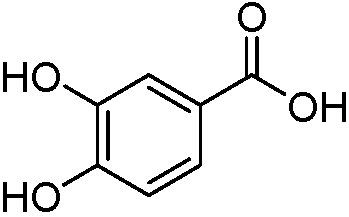
Hydroxybenzaldeyde	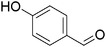
Vanillic acid	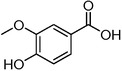
Caffeic acid	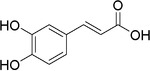
Vanillin	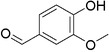
*o*‐Coumaric acid	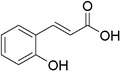
Resveratrol	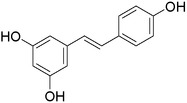
*trans*‐Ferulic acid	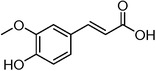
Sinapic acid	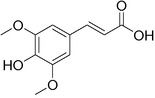
Hesperidin	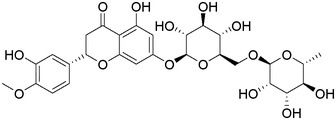
Isoquercitrin	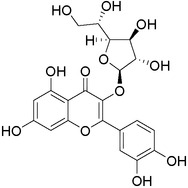
Kaempferol‐3‐glucoside	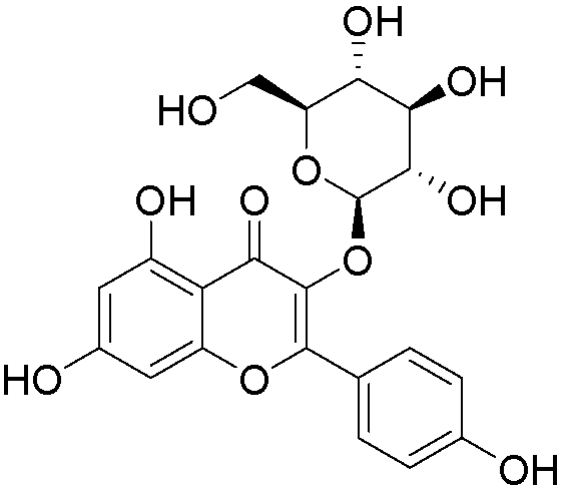
Fisetin	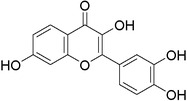
Luteolin	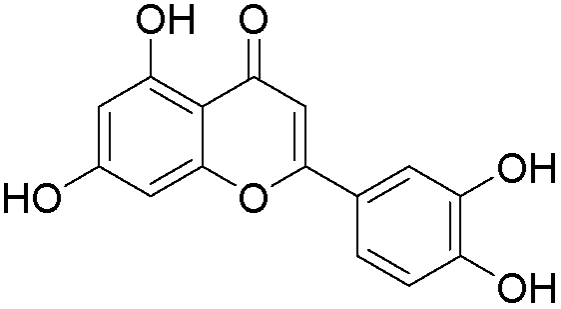

The analysis of the phenolic substances of *I. persica* L. subsp. *persica* was carried out spectrophotometrically with LC–MS/MS. As a result, 16 compounds were identified by determining the retention times (Rt), the amount of each compound, and the molecular ion (*m/z*) of the discovered compounds (Table 2). In *I. persica* L. subsp. *persica*, 1.935 g/kg of individual phenolic compounds were determined in methanol extract, and 2.137 g/kg of unique phenolic compounds were identified in aqueous extract. The most prevalent compound in methanol extract was shikimic acid, which had a mass of 1.324 g/kg, followed by vanillic acid, 0.205 g/kg, and hesperidin, 0.156 g/kg. Additionally, the most prevalent compound in the aqueous extract was shikimic acid, which had a mass of 1.356 g/kg, followed by vanillic acid, 0.429 g/kg, and *trans*‐ferulic acid, 0.206 g/kg.

**TABLE 2:** Quantitative phenolic contents of aqueous and methanol extracts, molecular ions, and retention times of the compounds.

Compounds	Molecular ion (*m/z*)	Ion mode	Rt (min)	Amount of phenolic compound (g/kg)	
				Aqueous extract	Methanol extract
Shikimic acid	173.0	Negative	1.351	1.356	1.324
Gallic acid	169.0	Negative	3.393	0.024	0.022
Protocatechuic acid	153.0	Negative	5.584	0.028	0.036
Hydroxybenzaldeyde	121.0	Negative	7.759	0.005	–
Vanillic acid	167.0	Negative	7.852	0.205	0.429
Caffeic acid	178.9	Negative	7.914	0.002	0.014
Vanillin	153.0	Positive	8.662	0.007	–
*o*‐Coumaric acid	163.0	Negative	9.425	0.019	0.039
Resveratrol	229.0	Positive	9.694	0.008	–
*trans*‐Ferulic acid	193.1	Negative	10.072	0.047	0.206
Sinapic acid	223.1	Negative	10.432	–	0.034
Hesperidin	611.0	Positive	11.624	0.156	–
Isoquercitrin	464.9	Positive	11.993	0.010	–
Kaempferol‐3‐glucoside	448.8	Positive	12.937	0.007	–
Fisetin	287.0	Positive	12.964	0.002	–
Luteolin	285.0	Positive	17.488	0.059	0.033

### 3.2 Antimicrobial activity results of *I. persica* aqueous extracts

In the antibacterial studies, the MIC value of the aqueous extract of *I. persica* against *S. aureus* was 22.5 mg/mL. Unlike this result, the MIC values of the aqueous extract against *E. aerogenes*, *P. aeruginosa*, *E. coli*, and *K. pneumoniae* were determined to be 11.25 mg/mL. Figure 2 shows that *S. aureus* showed slight growth in plate E, where the *I. persica* aqueous extract concentration was 11.25 mg/mL, growing dominantly from plate F onwards. *E. aerogenes*, *P. aeruginosa*, *E. coli*, and *K. pneumoniae* colonies started to appear from plate F, where the *I. persica* aqueous extract concentration was 5.625 mg/mL (Figure 2).

In antifungal studies conducted with the aqueous extract of *I. persica*, the highest inhibitory effect was observed against *C. parapsilosis* (MIC: 11.25 mg/mL). *C. parapsilosis* colonies were observed on the F plate, where the aqueous extract concentration was 5.625 mg/mL. *C. albicans* and *C. tropicalis* colonies started to grow on plate C, and the aqueous extract concentration in this plate was 45 mg/mL. Therefore, the MIC value of *I. persica* aqueous extract against *C. albicans* and *C. tropicalis* was determined to be 90 mg/mL. In the study, no inhibitory effect was observed against *C. krusei*. As seen in Figure 3, dense *C. krusei* colonies were observed from the first plate, where the highest *I. persica* aqueous extract concentration was used, to plate E. Starting from the F plate, *C. krusei* colonies regained their normal appearance as in the control plate (Figure 3).

Antibacterial and antifungal studies performed using the agar dilution method were repeated using the broth microdilution method. The MIC values of plant extracts used against bacteria and fungi were the same as those of studies using the agar dilution method. The pink color in the microplate indicates growth in wells, and the blue color means growth inhibition. In the study conducted by observing the color change, the MIC values of the aqueous extract of *I. persica* against *S. aureus*, *E. aerogenes*, *P. aeruginosa*, *E. coli*, *K. pneumoniae*, *C. albicans*, *C. tropicalis*, and *C. parapsilosis* were determined as 22.5, 11.25, 11.25, 11.25, 11.25, 90, 90, and 11.25 mg/mL, respectively (Figure 4).

### 3.3 Antimicrobial activity results of *I. persica* methanol extracts

The test results to determine the inhibitory effect of the methanol extract of *I. persica* against bacteria revealed different MIC values against each microorganism. Since *S. aureus* grew from the E plate with a methanol extract concentration of 11.25 mg/mL, the MIC value of the methanol extract against *S. aureus* was found to be 22.5 mg/mL. While *E. aerogenes* and *E. coli* grew slightly on the C plate, they showed dominant growth on the D plate. Therefore, the MIC value of the methanol extract against *E. aerogenes* and *E. coli* was found to be 90 mg/mL. Since *P. aeruginosa* started to grow on plate D, the MIC value of the methanol extract was determined to be 45 mg/mL. *K. pneumoniae* colonies were observed on plate F, where the methanol extract concentration of *I. persica* was 5.625 mg/mL, so the extract's MIC value was 11.25 mg/mL (Figure 5).

In antifungal studies conducted with the methanol extract of *I. persica*, as seen in the experiment with the aqueous extract, the highest inhibitory effect was observed against *C. parapsilosis*. *C. parapsilosis* colonies were observed from plate E, where the methanol extract concentration of *I. persica* was 11.25 mg/mL. Therefore, the MIC value against *C. parapsilosis* was 22.5 mg/mL. The MIC value of the methanol extract of *I. persica* against *C. albicans* and *C. tropicalis* was determined to be 180 mg/mL. On the other hand, no inhibitory effect was observed against *C. krusei*. On the contrary, and interestingly, the methanol extract of *I. persica* induced the growth of this species (Figure 6).

When antimicrobial testing was repeated using the broth microdilution method, the results and the MIC values found were the same for each type of microorganism (Table 1). Each well on the microplate with a color change indicates growth. Wells where there is no color change also indicate that growth is not occurring and that the extracts inhibit microorganisms. As a result, the MIC values of *I. persica* methanol extract against bacterial strains *S. aureus*, *E. coli*, *E. aerogenes*, *P. aeruginosa*, and *K. pneumonia* were determined to be 22,5 mg/mL in the 4th, 90 mg/mL in the 2nd, 45 mg/mL in the 3rd, 90 mg/mL in the 2nd, and 11.5 mg/mL in the 5th wells with no color change, respectively. The MIC values of the *I. persica* methanol extract against *C. albicans* and *C. tropicalis* were found to be 180 mg/mL in the 1st well, and they were found to be 22.5 mg/mL against *C. parapsilosis* in the 4th well (Figure 7). Whole experiments were performed three times, and the standard deviation was zero.

### 3.4 Results of molecular docking analysis

Active binding sites for each receptor have been previously calculated or determined based on the X‐ray crystallographic structures of 1EA1 and 1HSK. Docking studies were performed to see the interaction modes of all phenolic compounds with the active sites of macromolecules in light of these data. Binding types and associated residues were generated in detail by Maestro Software (Figures 8 and 9). It has previously been shown that residues TYR76, PHE78, MET79, PHE83, ARG96, LEU100, PHE255, ALA256, and LEU321 of Cytochrome P450 14 alpha‐sterol demethylase and cocrystalline ligand Fluconazole (PDB ID: TPF) are important for the interaction (EMBL‐EBI, 2023a). Here, it has been determined that some of our compounds, similar to Fluconazole, make hydrogen bonds with ARG96 and PHE255, and also show pi–pi interactions with TYR76, PHE78, and HEM460 (Table 4). The interaction modes with 1EA1 for all compounds were visualized in 2D and 3D with the Maestro program (Figures 1S–32S).

It has previously been shown that some of its residues are important for the interaction of *S. aureus* Murb and its cofactor FAD (EMBL‐EBI, 2023b). Some of our compounds made hydrogen bonds with TYR42, TYR77, GLY79, ASN80, SER82, ASN83, PRO141, SER143, GLY146, GLY153, LEU197, VAL199, ARG225, SER235, H2O554, H2O558, H2O600, H2O684, and H2O687, similar to FAD in 1HSK, and also showed pi–pi interaction with TYR149 and PHE274 (Table 5). The interaction modes with 1HSK for all compounds were visualized in 2D and 3D with the Maestro program (Figures 33S–64S).

## 4 DISCUSSION

Bioactive components such as glycosides, alkaloids, flavonoids, tannins, iridals, steroids, phenols, carbohydrates, phytosterols, terpenoids, stilbenes, and saponins found in *Iris* species have demonstrated medically important pharmacological activities as primary and secondary metabolites. Important pharmacotherapeutic properties such as analgesic effect and antibacterial and antifungal activity have been attributed to these compounds detected due to phytochemical analyses in *Iris* species (Bilal et al., 2023; Hussain et al., 2021). In this study, as a result of our extraction study with *I. persica* L. subsp. *persica*, 16 phenolic compounds were found by LC–MS/MS analysis; shikimic acid was the most abundant compound in both aqueous (1.356 g/kg) and methanol (1.324 g/kg) extracts. Shikimic acid has antibacterial activity and causes nucleotide and massive K^+^ leakage from *S. aureus* and a significant change in microbial cell membranes (Bai et al., 2015, 2018). Shikimic acid destroys cell membrane permeability, reduces membrane fluidity, and disrupts the cell membrane and membrane integrity. It has been proven that shikimic acid degrades bacterial cell membrane proteins and lipids and causes cell death by binding to membrane proteins (Bai et al., 2015). Shikimic acid disrupts protein synthesis by altering aminoacyl‐tRNA synthesis and ribosome functions, and it interferes with bacterial pyruvate metabolic pathways (Bai et al., 2022). In this study, shikimic acid, which was dominant in the phenolic content of the extracts, significantly affected the antimicrobial results. Vanillic acid was the second most abundant compound in both aqueous (0.205 g/kg) and methanol (0.429 g/kg) extracts. Vanillic acid inhibits cell growth by causing intracellular ATP concentration, decreased pH and membrane potential, and changes in cell morphology. Vanillic acid suppresses bacterial biofilm formation by destroying bacterial cell membrane integrity and has strong antibacterial properties (Qian et al., 2019; Qian, Liu, et al., 2020a; Qian, Yang, et al., 2020b). In this study, the third most abundant compounds, after shikimic acid and vanillic acid, were hesperidin (0.156 g/kg) in the aqueous extract and *trans*‐ferulic acid (0.206 g/kg) in the methanol extract. Although the number of phenolic compounds in the aqueous extract (15/16 compounds) is higher, the total amount of chemicals in the aqueous extract is lower. The high number of hydroxyl groups in the structure of compounds such as hesperidin, isoquercitrin, and kaempferol‐3‐glucoside has resulted in a decrease in its solubility in methanol, which is slightly more apolar than water, and this resulted in the absence of these chemicals in the methanol extract (9/16 compounds).

So far, various results have been obtained in antimicrobial studies conducted with the essential oils and extracts of different *Iris* species. In the antibacterial study of *Iris hungarica* and *Iris sibirica*, aqueous and lipophilic extracts of the leaves and rhizomes were used. As a result, a weak bacteriostatic effect against bacterial strains was observed with inhibition zone diameters of 15–19 and 14–16 mm in the aqueous and lipophilic extracts, respectively (MICs ranging between 125 and 500 μg/mL) (Kovalev et al., 2017). In the antimicrobial study with the ethanolic extract of *Iris germanica*, the disc diffusion method was used, and only limited antimicrobial activity was observed against *Bacillus subtilis* (Basgedik et al., 2014). In the study conducted with the extract of rhizomes, leaves, and flowers of *Iris nigricans*, antimicrobial activity was studied using the disc diffusion method, and antifungal and antibacterial activities were evaluated according to the inhibition zones formed (Al‐Khateeb et al., 2013). Moreover, concentrations of *Iris unguicularis* extract ranging between 25 and 100 μg/mL have been reported to inhibit bacterial strains using the disc diffusion method (Bensari et al., 2020). In the study conducted on the antibacterial activity of *Iris pseudopumila* methanol extract using the broth dilution method, the MIC value of the plant extract was found to be between 7.8 and 250 μg/mL, and especially *E. coli* and *E. aerogenes* were found to be more sensitive than other bacterial species (Rigano et al., 2006). Ramtin et al.'s study on the antimicrobial activity of *Iris pseudacorus* essential oil against bacterial species reported that the minimum bacteriocidal concentration of essential oils against bacteria was 15–30 μg/mL (Ramtin et al., 2014). In most studies conducted with the *Iris* genus, the disc diffusion method was used, and MIC values were not determined.

Hussain et al. reported that the antibacterial properties of the methanol extract of *I. persica* were investigated and MIC values were determined; however, it was observed that there was no antimicrobial study in the referenced source (Hussain et al., 2021). In that reference, the activity of the plant on human tumor cells was examined and evaluated using the MTT method (Amin et al., 2018). There has been no study on the antimicrobial properties of *I. persica* methanol and aqueous extract. However, in a study conducted using *I. persica* essential oil, moderate antifungal activity was observed, but MIC values were not determined, and the mechanism of activity was not explained (Amin et al., 2017). In this study, *I. persica* extracts had the same inhibitory effect on *S. aureus* and *K. pneumoniae*, but methanol extracts had more effective inhibitory activity on other bacterial species. Therefore, we cannot say that the inhibitory effect of *I. persica* extracts is selectively more substantial on Gram (+) or Gram (−) bacteria. The results show bacterial species were more sensitive to plant extracts than *Candida* species. In the antifungal study, we found that both extracts strongly inhibited the growth of *C. albicans, C. tropicalis*, and *C. parapsilosis* while interestingly supporting the growth of *C. krusei*. Especially in the first plates, where plant extract concentrations were high, *C. krusei* colonies were significantly more extensive and numerous than other species (Figures 3 and 6). *C. krusei* is the most resistant species within the *Candida* genus against antifungal drugs, such as fluconazole, and is an infectious agent with a high mortality rate (40%–58%) (Gómez‐Gaviria & Mora‐Montes, 2020; Rodrigues et al., 2023). The metabolism of *C. krusei* has yet to be adequately studied to date. When the genome of *C. krusei* was examined, it was confirmed that this species did not belong to the CUG clade of the *Candida* genus and was different from *C. albicans* (Gómez‐Gaviria & Mora‐Montes, 2020). It is thought that this unique difference of *C. krusei* is also reflected in the antimicrobial study we conducted. Therefore, due to the supportive effect of plant extracts on *C. krusei* growth, their use as therapeutic agents in fungal diseases requires more attention.

It is seen in molecular docking results (docking scores) that especially hydroxybenzaldeyde, vanillin, resveratrol, isoquercitrin, kaempferol‐3‐glucoside, fisetin, and luteolin are more prone to antifungal activity than antibacterial activity, and none of these compounds (except luteolin) are found in the methanol extract (Table 2). For this reason, in vitro results demonstrated that the antifungal activity in methanol extract was lower than that in aqueous extract, and this was also explained by in silico results. The absence of these compounds, which have higher affinities than others in silico analyses, in the methanol extract caused it to have less biological activity than the aqueous extract (Table 3). If the percentages of these compounds in the extract were higher, it would be expected that they would be more effective in terms of biological activity, especially the antifungal activity. When the in silico analyses of shikimic acid, which has the highest amount in both aqueous and methanol extracts, are examined, it can be said that the affinity for antibacterial activity is higher than the antifungal activity. Therefore, both extracts are prone to antimicrobial activity when viewed with in vitro results (Tables 2, 3, 4, and 5).

**TABLE 3:** MIC values of *I. persica* aqueous and methanol extract against the microorganisms.

Microorganisms	*I. persica* aqueous extract (mg/mL)	*I. persica* methanol extract (mg/mL)
*S. aureus*	22.5	22.5
*E. aerogenes*	11.25	90
*P. aeruginosa*	11.25	45
*E. coli*	11.25	90
*K. pneumoniae*	11.25	11.25
*C. albicans*	90	180
*C. krusei*	ND	ND
*C. tropicalis*	90	180
*C. parapsilosis*	11.25	22.5

Abbreviation: ND, not detected.

**TABLE 4:** Molecular docking scores, binding types, and estimated inhibition constants of all compounds on 1EA1.

Compound	Interacting residues based on visual results		Autodock		Vina
	H bond	pi–pi interaction	Estimated inhibition constant, Ki	Best dock score	Best dock score
Shikimic acid	ARG96	–	57.11 μM	−5.79	−6.3
Gallic acid	PHE255	HEM460	134.55 μM	−5.28	−6.2
Protocatechuic acid	–	–	78.70 μM	−5.60	−6.2
Hydroxybenzaldeyde	–	–	29.41 μM	−6.18	−5.8
Vanillic acid	–	HEM460	346.56 μM	−4.72	−6.5
Caffeic acid	TYR77, ASN80, SER143		10.54 μM	−6.79	−6.9
Vanillin	–	HEM460	68.85 μM	−5.68	−6.0
*o‐*Coumaric acid	–	HEM460	45.01 μM	−5.93	−6.9
Resveratrol	MET433	TYR76	686.01 nM	−8.41	−8.8
*trans‐*Ferulic acid	H_2_O‐HEM460	HEM460	38.28 μM	−6.03	−6.9
Sinapic acid	–	–	116.69 μM	−5.37	−7.0
Hesperidin	GLY84, MET433	–	3.33 μM	−7.46	−8.1
Isoquercitrin	SER252, MET433	–	394.07 nM	−8.74	−7.4
Kaempferol‐3‐glucoside	PHE255, ILE323, H_2_O‐HEM460	TYR76, PHE78	414.39 nM	−8.71	−7.8
Fisetin	MET433	TYR76, PHE78	181.59 nM	−9.27	−9.2
Luteolin	MET433	–	241.22 nM	−9.03	−7.7

Abbreviations: μM, micromolar; nM, nanomolar; Docking Score, Estimated Binding Free Energy (kcal/mol).

**TABLE 5:** Molecular docking scores, binding types, and estimated inhibition constants of all compounds on 1HSK.

Compound	Interacting residues based on visual results		Autodock		Vina
	H bond	Pi‐pi interaction	Estimated inhibition constant, Ki	Best dock score	Best dock score
Shikimic acid	ASN80, SER143, GLY146	–	35.28 μM	−6.07	−7.0
Gallic acid	ASN80, SER143	–	48.34 μM	−5.89	−7.1
Protocatechuic acid	ASN80, PRO141, SER143	–	39.02 μM	−6.01	−7.0
Hydroxybenzaldeyde	ASN80, SER143, GLY146, H_2_O554	–	232.14 μM	−4.96	−6.0
Vanillic acid	ASN80, ASN83, SER143	–	35.56 μM	−6.07	−6.8
Caffeic acid	TYR77, ASN80, SER143, H_2_O687	–	5.60 μM	−7.16	−7.5
Vanillin	SER82, SER143, H_2_O600	–	129.11 μM	−5.31	−6.4
*o*‐Coumaric acid	TYR77, GLY79, ASN80, SER143	–	5.90 μM	−7.13	−7.4
Resveratrol	ASN83, SER143, VAL199	–	1.03 μM	−8.17	−8.1
*trans*‐Ferulic acid	ASN80, SER143, H_2_O687	TYR149	7.90 μM	−6.96	−7.7
Sinapic acid	ASN80, SER82, SER143	–	9.03 μM	−6.88	−7.0
Hesperidin	TYR77, H_2_O600, H_2_O687	–	67.91 nM	−9.78	−9.7
Isoquercitrin	H_2_O558‐TYR42, SER82, GLY153, ARG225, H_2_O600, H_2_O558‐SER235	PHE274	10.03 μM	−6.82	−8.7
Kaempferol‐3‐Glucoside	SER82, ASN83, GLY153, GLY237	PHE274	2.95 μM	−7.54	−8.4
Fisetin	ASN80, SER143, LEU197, VAL199, H_2_O684	–	662.70 nM	−8.43	−9.3
Luteolin	SER82, SER143, VAL199	–	750.23 nM	−8.36	−9.5

Abbreviations: μM, micromolar; nM, nanomolar; Docking Score, Estimated Binding Free Energy (kcal/mol).

When the test results of the plant aqueous and methanol extracts are compared, bacterial and fungal species are more sensitive to the aqueous extract than the methanol extract. The microorganisms used were slightly more resistant to methanol extracts. According to the LS‐MS/MS analysis results, the contents and amounts of both extracts were very close to each other. However, hesperidin, isoquercitrin, resveratrol, kaempferol‐3‐glucoside, vanillin, hydroxybenzaldeyde, and fisetin were compounds found in the aqueous extracts but not in the methanol extract. Although these compounds, except for hesperidin (0.156 g/kg), were found at minimal levels (between 0.010 and 0.002 g/kg), they might affect the difference in antimicrobial activity between the aqueous and methanol extract results. Nevertheless, among the compounds not found in the methanol extract but in the aqueous extract, the dominant compound is hesperidin. Hesperidin belongs to the flavonoid family and is one of the most common polyphenols. Hesperidin consists of an aglycone unit called hesperetin and a disaccharide, rutinose, and its antimicrobial activity has been reported (Abuelsaad et al., 2013; Choi et al., 2022; Iranshahi et al., 2015). The exact mechanisms of its antimicrobial activity have yet to be elucidated. However, it is predicted to act through various mechanisms, such as bacterial membrane disruption, activation of the host immune system, and interference with microbial enzymes (Iranshahi et al., 2015). Additionally, luteolin was almost twice as abundant in the aqueous extracts (0.059 g/kg) as in the methanol extract (0.033 g/kg) in this study. Samples treated with luteolin showed a significant increase in dead microbial cells. Luteolin destroys cell membrane integrity by causing changes in microbial cell morphology and has inhibitory effects on biofilm formation. Luteolin exerts a bacteriocidal effect on biofilm‐forming cells by increasing antibiotic diffusion within biofilms (Guo et al., 2020; Qian, Liu, et al., 2020a; Qian, Yang, et al., 2020b).

Considering the MIC values (Table 3), although the tested microorganisms were observed to be more sensitive to the plant aqueous extract, it cannot be said that the plant extracts have a selective inhibitory effect against Gram (−) or Gram (+) microorganisms. The most potent inhibitory effect of plant methanol and aqueous extracts against the tested bacterial species was determined against *K. pneumoniae* and *S. aureus*, with MIC values of 11.25 and 22.5 mg/mL, respectively. For *Candida* species, the most substantial inhibition was against *C. parapsilosis*, and the MIC values of aqueous and methanol extracts were determined to be 11.25 and 22.25 mg/mL, respectively. Therefore, MIC values determined against the tested bacteria and *Candida* strains are distributed irregularly, regardless of the type of microorganism. In this study, the antimicrobial activity and mechanism of plant extract ingredients found using LC–MS/MS analysis were also supported by a literature review. Accordingly, *I. persica* extracts would enable the development of therapeutic combination drugs for infectious diseases, and they could reduce the severity of side effects and the stress of chemotherapeutic drugs. Thus, it can potentially be used as a natural protectant to control infections.

## 5 CONCLUSION

Consequently, the methanol and aqueous extracts have the same inhibitory effect against *S. aureus* and *K. pneumonia*. In contrast, aqueous extract has a predominant inhibitory effect against other tested microorganisms compared with methanol extract. Interestingly, while plant extracts induced the growth of *C. krusei*, *C. parapsilosis* was found to be more sensitive to both *I. persica* methanol and aqueous extracts than other *Candida* strains, followed by *C. albicans* and *C. tropicalis*. It has been revealed that phenolic compounds may show significant activity for different drug targets in antifungal and antibacterial treatments by forming good interactions with the target binding sites of macromolecules. When the chemical analysis, in vitro, and in silico results were evaluated together, it was seen that hydroxybenzaldeyde, vanillin, resveratrol, isoquercitrin, kaempferol‐3‐glucoside, fisetin, and luteolin were more prone to antifungal activity than antibacterial activity, and none of these compounds were found in the methanol extract (except luteolin). Therefore, in vitro results showed that the antimicrobial activity in methanol extract was lower than in aqueous extract, which was also explained by in silico results.

## AUTHOR CONTRIBUTIONS

**Tuba Unver:** Conceptualization (lead); data curation (equal); formal analysis (equal); investigation (lead); methodology (equal); project administration (lead); supervision (lead); validation (equal); writing – original draft (equal); writing – review and editing (equal). **Harun Uslu:** Data curation (supporting); formal analysis (equal); investigation (supporting); methodology (equal); software (lead); validation (equal); visualization (lead); writing – original draft (supporting); writing – review and editing (supporting). **Ismet Gurhan:** Data curation (equal); formal analysis (equal); investigation (equal); methodology (equal); validation (equal). **Bunyamin Goktas:** Formal analysis (supporting); investigation (supporting); methodology (supporting); software (supporting); validation (supporting); visualization (supporting).

## CONFLICT OF INTEREST STATEMENT

None declared.

## Supporting information


Data S1

## Data Availability

The data that support the findings of this study are available on request from the corresponding author.

## References

[fsn34251-bib-0001] Abhishek, K. , Ashutos, M. , & Sinha, B. N. (2006). Herbal drugs‐present status and efforts to promote and regulate cultivation. Pharmacy Review, 6(1), 73–77.

[fsn34251-bib-0002] Abuelsaad, A. S. , Mohamed, I. , Allam, G. , & Al‐Solumani, A. A. (2013). Antimicrobial and immunomodulating activities of hesperidin and ellagic acid against diarrheic Aeromonas hydrophila in a murine model. Life Sciences, 93(20), 714–722. 10.1016/j.lfs.2013.09.019 24090709

[fsn34251-bib-0003] Al‐Khateeb, E. , Finjan, S. , & Maraqa, A. (2013). Antioxidant and antimicrobial activities of *Iris nigricans* methanolic extracts containing phenolic compounds. European Scientific Journal, 9(3), 83–91.

[fsn34251-bib-0004] Amin, H. I. , Ibrahim, M. F. , Hussain, F. H. , Marcello, M. , & Vidari, G. (2018). Bioactive constituents from the traditional Kurdish plant *Iris persica* . Natural Product Communications, 13(9), 1127–1128. 10.1177/1934578X1801300907

[fsn34251-bib-0005] Amin, H. I. M. , Amin, A. A. , Tosi, S. , Mellerio, G. G. , Hussain, F. H. S. , Picco, A. M. , & Vidari, G. (2017). Chemical composition and antifungal activity of essential oils from flowers, leaves, rhizomes, and bulbs of the wild Iraqi Kurdish plant *Iris persica* . Natural Product Communications, 12(3), 441–444. 30549906

[fsn34251-bib-0006] Bai, J. , Wu, Y. , Bu, Q. , Zhong, K. , & Gao, H. (2022). Comparative study on antibacterial mechanism of shikimic acid and quinic acid against *Staphylococcus aureus* through transcriptomic and metabolomic approaches. LWT, 153, 112441. 10.1016/j.lwt.2021.112441

[fsn34251-bib-0007] Bai, J. , Wu, Y. , Liu, X. , Zhong, K. , Huang, Y. , & Gao, H. (2015). Antibacterial activity of Shikimic acid from pine needles of *Cedrus deodara* against *Staphylococcus aureus* through damage to cell membrane. International Journal of Molecular Sciences, 16(11), 27145–27155. 10.3390/ijms161126015 26580596 PMC4661873

[fsn34251-bib-0008] Bai, J. , Wu, Y. , Zhong, K. , Xiao, K. , Liu, L. , Huang, Y. , Wang, Z. , & Gao, H. (2018). A comparative study on the effects of Quinic acid and Shikimic acid on cellular functions of *Staphylococcus aureus* . Journal of Food Protection, 81(7), 1187–1192. 10.4315/0362-028X.JFP-18-014 29939792

[fsn34251-bib-0009] Basgedik, B. , Ugur, A. , & Sarac, N. (2014). Antimicrobial, antioxidant, antimutagenic activities, and phenolic compounds of Iris germanica. Industrial Crops and Products, 61, 526–530. 10.1016/j.indcrop.2014.07.022

[fsn34251-bib-0010] Basser, K. , Demirci, B. , & Orhan, I. (2011). Composition of volatiles from three iris species of Turkey. Journal of Essential Oil Research, 4(23), 66–71. 10.1080/10412905.2011.9700471

[fsn34251-bib-0011] Bensari, S. , Ouelbani, R. , Yimaz, M. A. , Bensouici, C. , Gokalp, E. , & Khelifi, D. (2020). Phytochemical profiles of Iris unguicularis Poir. With antioxidant, antibacterial, and anti‐Alzheimer activities. Acta Natura et Scientia, 7, 74–87. 10.2478/asn-2020-0021

[fsn34251-bib-0012] Benson, T. E. , Harris, M. S. , Choi, G. H. , Cialdella, J. I. , Herberg, J. T. , Martin, J. P., Jr. , & Baldwin, E. T. (2001). A structural variation for MurB: X‐ray crystal structure of *Staphylococcus aureus* UDP‐N‐acetylenolpyruvylglucosamine reductase (MurB). Biochemistry, 40(8), 2340–2350. 10.1021/bi002162d 11327854

[fsn34251-bib-0013] Berger‐Bächi, B. (2002). Resistance mechanisms of gram‐positive bacteria. International Journal of Medical Microbiology, 292(1), 27–35. 10.1078/1438-4221-00185 12139425

[fsn34251-bib-0014] Bilal, M. , Naz, A. , Khan, A. , Salman Ghaffar, R. , & Abrar, A. (2023). Assessment of *Iris albicans* lange as potential antimicrobial and analgesic agent. PLoS One, 18(1), e0280127. 10.1371/journal.pone.0280127 36607998 PMC9821482

[fsn34251-bib-0015] Centers for Disease, Control and Prevention . (2021). 2021 National and State Healthcare‐Associated Infections Progress Report. 140 p. https://www.cdc.gov/hai/data/portal/progress‐report.html

[fsn34251-bib-0016] Choi, S. S. , Lee, S. H. , & Lee, K. A. (2022). A comparative study of hesperetin, hesperidin and hesperidin glucoside: Antioxidant, anti‐inflammatory, and antibacterial activities in vitro. Antioxidants, 11(8), 1618. 10.3390/antiox11081618 36009336 PMC9405481

[fsn34251-bib-0017] Clinical and Laboratory Standards Institute (Ed.). (2018). Methods for dilution antimicrobial susceptibility tests for bacteria that grow aerobically (11th ed.). Clinical and Laboratory Standards Institute.

[fsn34251-bib-0018] Clinical and Laboratory Standards Institute/National Committee for Clinical Laboratory Standards (CLSI/NCCLS) . (2005). *Performance standards for antimicrobial susceptibility testing*; fifteenth Information supplement. CLSI/NCCLS document M 100–S15. Wayne, PA.

[fsn34251-bib-0019] Costelloe, C. , Metcalfe, C. , Lovering, A. , Mant, D. , & Hay, A. D. (2010). Effect of antibiotic prescribing in primary care on antimicrobial resistance in individual patients: Systematic review and meta‐analysis. British Medical Journal, 340, c2096. 10.1136/bmj.c2096 20483949

[fsn34251-bib-0020] De Smet, P. A. (1997). The role of plant‐derived drugs and herbal medicines in healthcare. Drugs, 54(6), 801–840. 10.2165/00003495-199754060-00003 9421691

[fsn34251-bib-0021] Desam, N. R. , & Al‐Rajab, A. J. (2021). The importance of natural products in cosmetics. In Bioactive Natural Products for Pharmaceutical Applications (Vol. 140, pp. 643–685). Advanced Structured Materials book series, Springer, Cham. 10.1007/978-3-030-54027-2_19

[fsn34251-bib-0022] Dong, Y. H. , Wang, L. Y. , & Zhang, L. H. (2007). Quorum‐quenching microbial infections: Mechanisms and implications. Philosophical Transactions of the Royal Society of London. Series B, Biological Sciences, 362(1483), 1201–1211. 10.1098/rstb.2007.2045 17360274 PMC2435583

[fsn34251-bib-0023] EMBL‐EBI . (2023a). Protein Data Bank in Europe, Bringing Structure to Biology, TPF. https://www.ebi.ac.uk/pdbe/entry/pdb/1ea1/bound/TPF#470A 10.1107/S090744491004117X PMC3069747 21460450

[fsn34251-bib-0024] EMBL‐EBI . (2023b). Protein Data Bank in Europe, Bringing Structure to Biology, FAD. https://www.ebi.ac.uk/pdbe/entry/pdb/1hsk/bound/FAD#401A 10.1107/S090744491004117X PMC3069747 21460450

[fsn34251-bib-0025] Gómez‐Gaviria, M. , & Mora‐Montes, H. M. (2020). Current aspects in the biology, pathogeny, and treatment of *Candida krusei*, a neglected fungal pathogen. Infection and Drug Resistance, 13, 1673–1689. 10.2147/IDR.S247944 32606818 PMC7293913

[fsn34251-bib-0026] Gossell‐Williams, M. , Simon, O. R. , & West, M. E. (2006). The past and present use of plants for medicines. The West Indian Medical Journal, 55(4), 217–218. 10.1590/S0043-31442006000400002 17249308

[fsn34251-bib-0027] Güner, A. (2021). The Illustrated Flora of Turkey—*Iris* L./Süsen. 10.30796/ANGV.2021.9

[fsn34251-bib-0028] Guo, Y. , Liu, Y. , Zhang, Z. , Chen, M. , Zhang, D. , Tian, C. , Liu, M. , & Jiang, G. (2020). The antibacterial activity and mechanism of action of Luteolin against Trueperella pyogenes. Infection and Drug Resistance, 13, 1697–1711. 10.2147/IDR.S253363 32606820 PMC7293968

[fsn34251-bib-0029] Hussain, F. H. , Amin, H. I. M. , Patel, D. K. , & Porwal, O. (2021). An overview of the therapeutic potential of *Iris persica* . Current Traditional Medicine, 7(2), 152–160. 10.2174/2215083806666200117111320

[fsn34251-bib-0030] Ibrahim, S. R. , Mohamed, G. A. , & Al‐Musayeib, N. M. (2012). New constituents from the rhizomes of Egyptian *Iris germanica* L. Molecules, 17(3), 2587–2598. 10.3390/molecules17032587 22388969 PMC6268570

[fsn34251-bib-0031] Iranshahi, M. , Rezaee, R. , Parhiz, H. , Roohbakhsh, A. , & Soltani, F. (2015). Protective effects of flavonoids against microbes and toxins: The cases of hesperidin and hesperetin. Life Sciences, 137, 125–132. 10.1016/j.lfs.2015.07.014 26188593

[fsn34251-bib-0032] Kaatz, G. W. (2005). Bacterial efflux pump inhibition. Current Opinion in Investigational Drugs, 6(2), 191–198. 15751743

[fsn34251-bib-0033] Kaššák, P. (2012). Secondary metabolites of the chosen genus iris species. Acta Universitatis Agriculturae et Silviculturae Mendelianae Brunensis, 60, 269–280. 10.11118/actaun201260080269

[fsn34251-bib-0034] Kovalev, V. M. , Mykhailenko, O. O. , Krechun, A. V. , & Osolodchenko, T. P. (2017). Antimicrobial activity of extracts of Iris hungarica and *Iris sibirica* . Annals of Mechnikov Institute, 2, 57–64.

[fsn34251-bib-0035] Kukuła‐Koch, W. , Sienawska, E. , Widelski, J. , Urjin, O. , Głowniak, P. , & Skalicka‐Woźniak, K. (2015). Major secondary metabolites of iris spp. Phytochemistry Reviews, 14, 51–80. 10.1007/s11101-013-9333-1

[fsn34251-bib-0036] Levy, S. B. (2001). Antibiotic resistance: Consequences of inaction. Clinical Infectious Diseases, 33(3), S124–S129. 10.1086/321837 11524708

[fsn34251-bib-0037] Livermore, D. (2004b). Can better prescribing turn the tide of resistance? Nature Reviews. Microbiology, 2(1), 73–78. 10.1038/nrmicro798 15035011

[fsn34251-bib-0038] Livermore, D. M. (2004a). The need for new antibiotics. Clinical Microbiology and Infection, 10(4), 1–9. 10.1111/j.1465-0691.2004.1004.x 15522034

[fsn34251-bib-0039] Naja, F. , Alameddine, M. , Itani, L. , Shoaib, H. , Hariri, D. , & Talhouk, S. (2015). The use of complementary and alternative medicine among Lebanese adults: Results from a National Survey. Evidence‐based Complementary and Alternative Medicine, 2015, 682397. 10.1155/2015/682397 26106436 PMC4461758

[fsn34251-bib-0040] Nigam, A. , Gupta, D. , & Sharma, A. (2014). Treatment of infectious disease: Beyond antibiotics. Microbiological Research, 169(9–10), 643–651. 10.1016/j.micres.2014.02.009 24661689

[fsn34251-bib-0041] Podust, L. M. , Poulos, T. L. , & Waterman, M. R. (2001). Crystal structure of cytochrome P450 14alpha‐sterol demethylase (CYP51) from mycobacterium tuberculosis in complex with azole inhibitors. Proceedings of the National Academy of Sciences of the United States of America, 98(6), 3068–3073. 10.1073/pnas.061562898 11248033 PMC30608

[fsn34251-bib-0042] Qian, W. , Fu, Y. , Liu, M. , Wang, T. , Zhang, J. , Yang, M. , Sun, Z. , Li, X. , & Li, Y. (2019). In vitro antibacterial activity and mechanism of Vanillic acid against Carbapenem‐resistant *Enterobacter cloacae* . Antibiotics, 8(4), 220. 10.3390/antibiotics8040220 31766130 PMC6963763

[fsn34251-bib-0043] Qian, W. , Liu, M. , Fu, Y. , Zhang, J. , Liu, W. , Li, J. , Li, X. , Li, Y. , & Wang, T. (2020a). Antimicrobial mechanism of luteolin against *Staphylococcus aureus* and listeria monocytogenes and its antibiofilm properties. Microbial Pathogenesis, 142, 104056. 10.1016/j.micpath.2020.104056 32058023

[fsn34251-bib-0044] Qian, W. , Yang, M. , Wang, T. , Sun, Z. , Liu, M. , Zhang, J. , Zeng, Q. , Cai, C. , & Li, Y. (2020b). Antibacterial mechanism of Vanillic acid on physiological, morphological, and biofilm properties of Carbapenem‐resistant Enterobacter hormaechei. Journal of Food Protection, 83(4), 576–583. 10.4315/JFP-19-469 31855457

[fsn34251-bib-0045] Radulović, N. , Stankov‐Jovanović, V. , Stojanović, G. , Šmelcerović, A. , Spiteller, M. , & Asakawa, Y. (2007). Screening of in vitro antimicrobial and antioxidant activity of nine Hypericum species from the Balkans. Food Chemistry, 103(1), 15–21. 10.1016/j.foodchem.2006.05.062

[fsn34251-bib-0046] Ramtin, M. , Massiha, A. , Khoshkholgh‐Pahlaviani, M. R. M. , Issazadeh, K. , Assmar, M. , & Zarrabi, S. (2014). In vitro antimicrobial activity of *Iris pseudacorus* and *Urtica dioica* . Zahedan Journal of Research in Medical Sciences, 16(3), 35–39.

[fsn34251-bib-0047] RCSB PDB, RCSB Protein Data Bank . (2023). https://www.rcsb.org/

[fsn34251-bib-0048] Reygaert, W. C. (2018). An overview of the antimicrobial resistance mechanisms of bacteria. AIMS Microbiology, 4(3), 482–501. 10.3934/microbiol.2018.3.482 31294229 PMC6604941

[fsn34251-bib-0049] Rigano, D. , Conforti, F. , Formisano, C. , Menichini, F. , & Senatore, F. (2009). Comparative free radical scavenging potential and cytotoxicity of different extracts from iris pseudopumila Tineo flowers and rhizomes. Natural Product Research, 23(1), 17–25. 10.1080/14786410701740237 19140069

[fsn34251-bib-0050] Rigano, D. , Grassia, A. , Formisano, C. , Basile, A. , Sorbo, S. , & Sorbo, F. (2006). Antibacterial and allelopathic activity of methanolic extract from iris pseudopumila rhizomes. Fitoterapia, 77, 460–462. 10.1016/j.fitote.2006.05.009 16814956

[fsn34251-bib-0051] Rodrigues, A. B. F. , Passos, J. C. D. S. , & Costa, M. S. (2023). Effect of antimicrobial photodynamic therapy using toluidine blue on dual‐species biofilms of *Candida albicans* and *Candida krusei* . Photodiagnosis and Photodynamic Therapy, 42, 103600. 10.1016/j.pdpdt.2023.103600 37150491

[fsn34251-bib-0052] Sanner, M. F. (1999). Python: A programming language for software integration and development. Journal of Molecular Graphics & Modelling, 17(1), 57–61. 10660911

[fsn34251-bib-0053] Trott, O. , & Olson, A. J. (2010). AutoDock Vina: Improving the speed and accuracy of docking with a new scoring function, efficient optimization, and multithreading. Journal of Computational Chemistry, 31(2), 455–461. 10.1002/jcc.21334 19499576 PMC3041641

[fsn34251-bib-0054] Unver, T. , Erenler, A. S. , Bingul, M. , & Boga, M. (2023). Comparative analysis of antioxidant, anticholinesterase, and antibacterial activity of microbial chondroitin sulfate and commercial chondroitin sulfate. Chemistry & Biodiversity, 20(10), e202300924. 10.1002/cbdv.202300924 37615364

[fsn34251-bib-0055] Unver, T. , & Gurhan, I. (2024). Chemical composition and antimicrobial activity of an apolar extract from *Lactuca serriola* L. leaves. Biochemical Systematics and Ecology, 114, 104832. 10.1016/j.bse.2024.104832

[fsn34251-bib-0056] Venditti, A. , Frezza, C. , Rai, R. , Sciubba, F. , Di Cecco, M. , Ciaschetti, G. , Serafini, M. , & Bianco, A. (2017). Isoflavones and other compounds from the roots of *Iris marsica* I. Ricci E colas. Collected from Majella National Park, Italy. Medicinal Chemistry, 7(2), 787–794. 10.4172/2161-0444.1000430

[fsn34251-bib-0057] WHO . (1996). Technical report series. Guidelines for the Assessment of Herbal Medicines, 863(1), 178–184.

[fsn34251-bib-0058] WHO . (2002). Traditional medicine ‐ growing needs and potential. WHO Policy Perspective Medicine, 2(1), 1–6.

[fsn34251-bib-0059] Yousefsani, B. S. , Boozari, M. , Shirani, K. , Jamshidi, A. , & Dadmehr, M. (2021). A review on phytochemical and therapeutic potential of Iris germanica. The Journal of Pharmacy and Pharmacology, 73(5), 611–625. 10.1093/jpp/rgab008 33772287

