# Supplementary material for: Screening of phenolic components and antimicrobial properties of Iris persica L. subsp. persica extracts by in vitro and in silico methods
Source: Food Sci Nutr. 2024 Jun 24;12(9):6578–94. doi: 10.1002/fsn3.4251 (PMC11561777; doi:10.1002/fsn3.4251)
Supplement: Supplementary file 1 — Data S1 [file FSN3-12-6578-s001.docx]

**
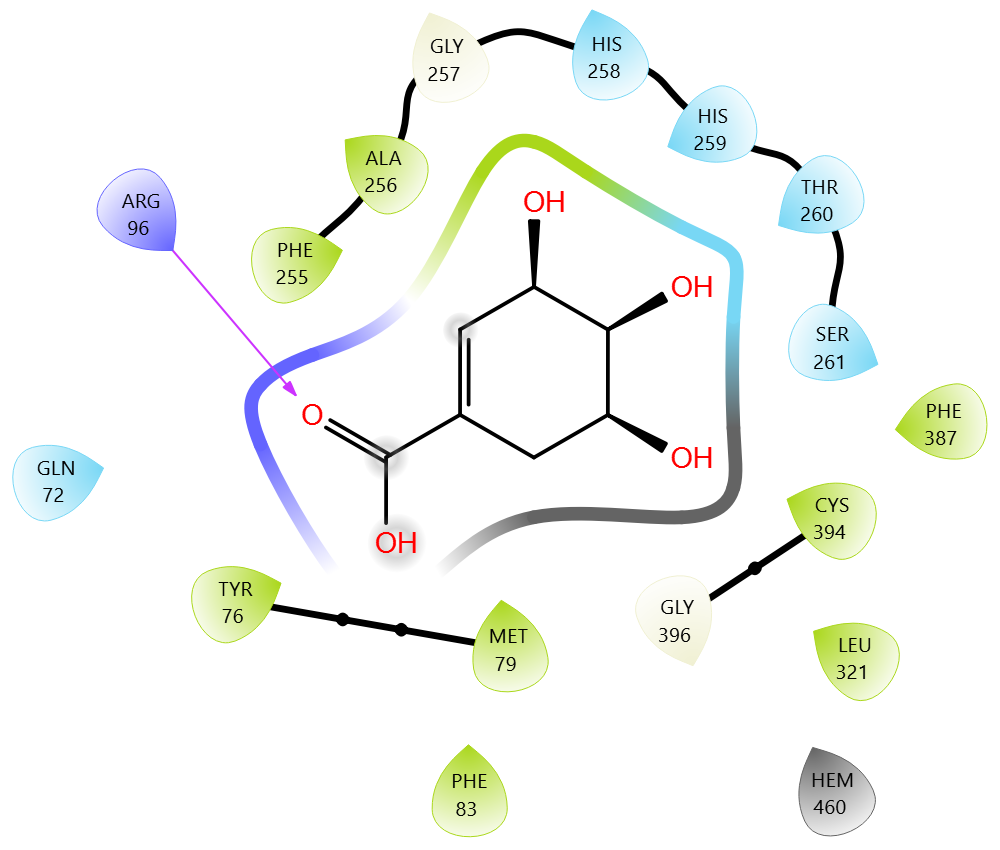
**

**Figure 1S.** 2D interaction diagram with 1EA1 for shikimic acid.


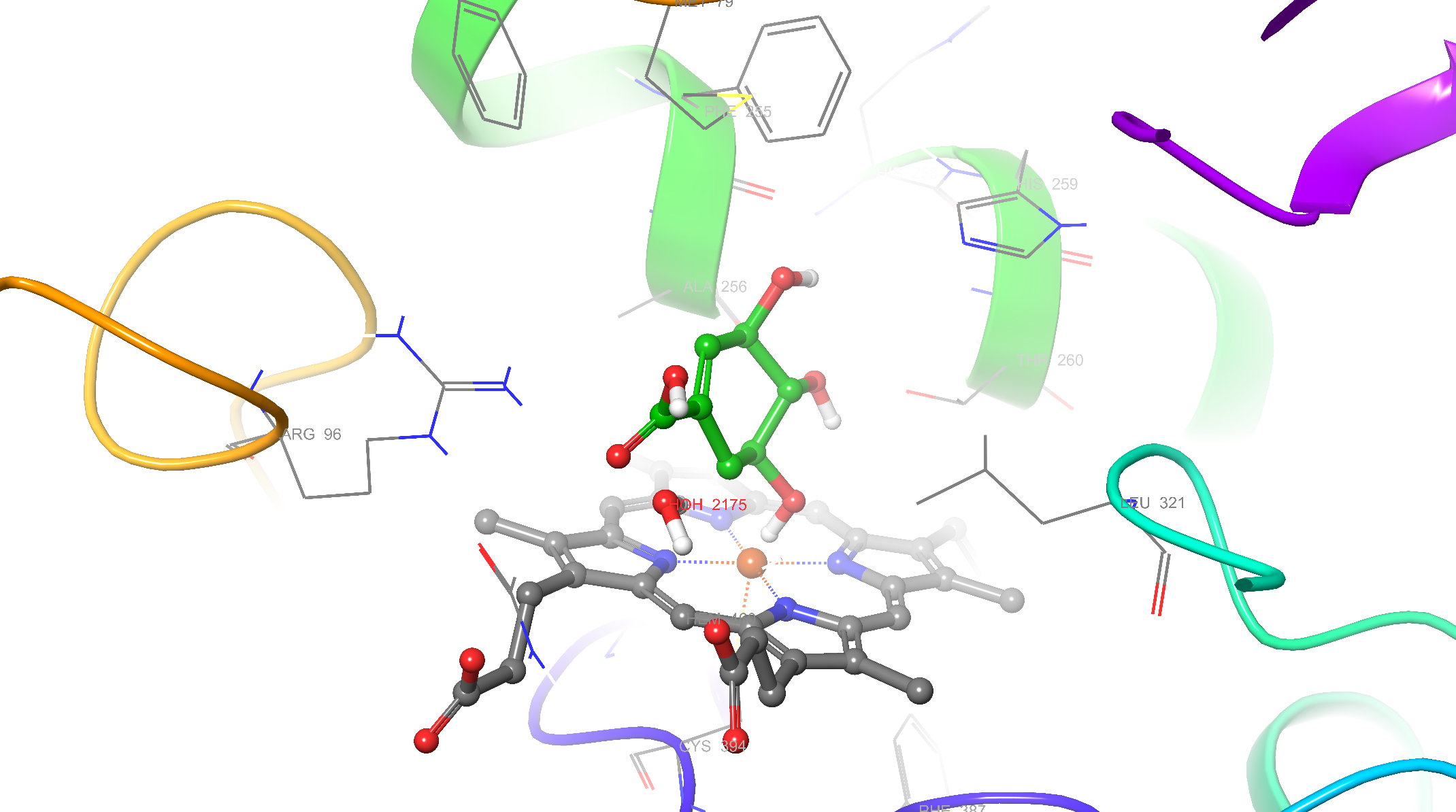


**Figure 2S.** 3D interaction diagram with 1EA1 for shikimic acid.

**
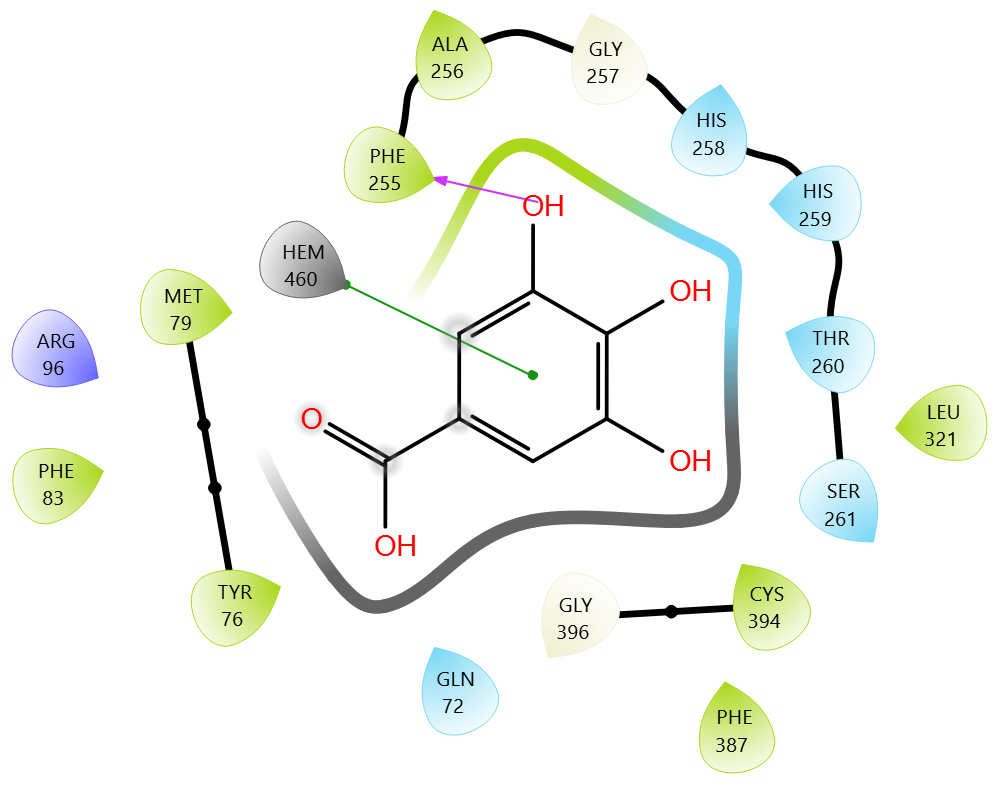
**

**Figure 3S.** 2D interaction diagram with 1EA1 for gallic acid.


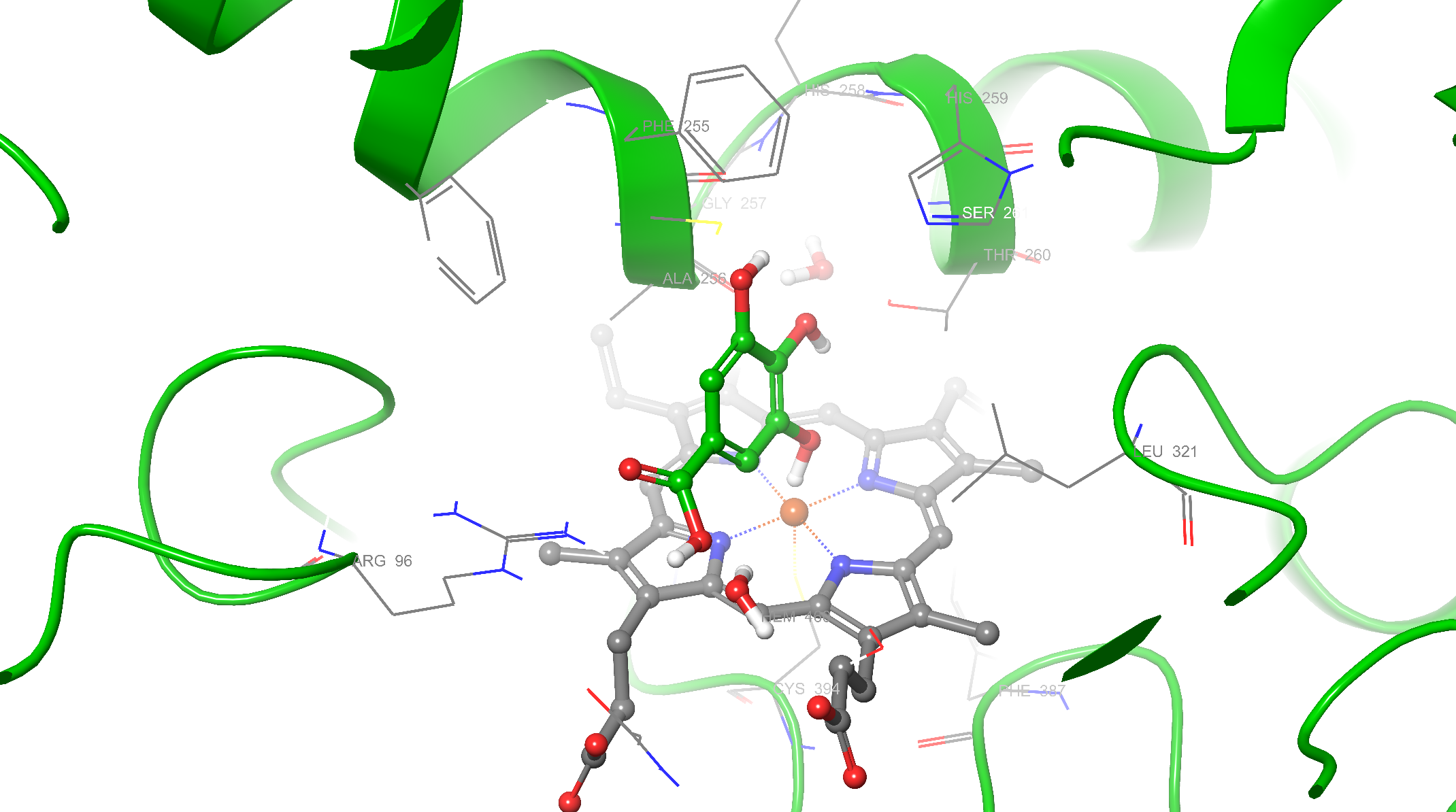


**Figure 4S.** 3D interaction diagram with 1EA1 for gallic acid.

**
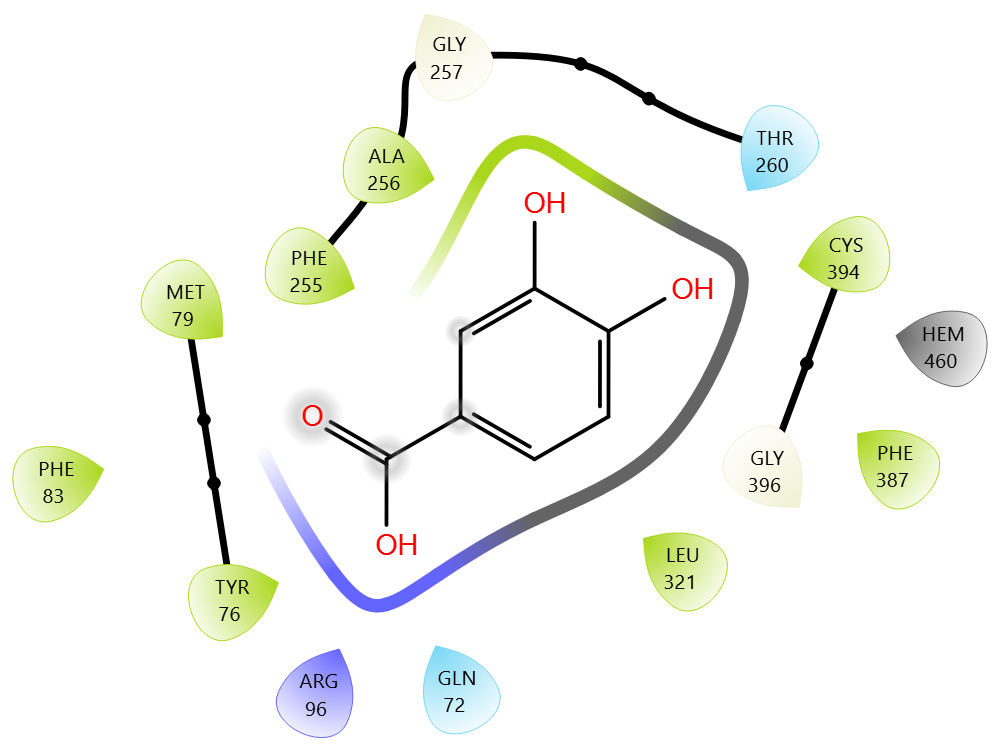
**

**Figure 5S.** 2D interaction diagram with 1EA1 for protocatechuic acid.


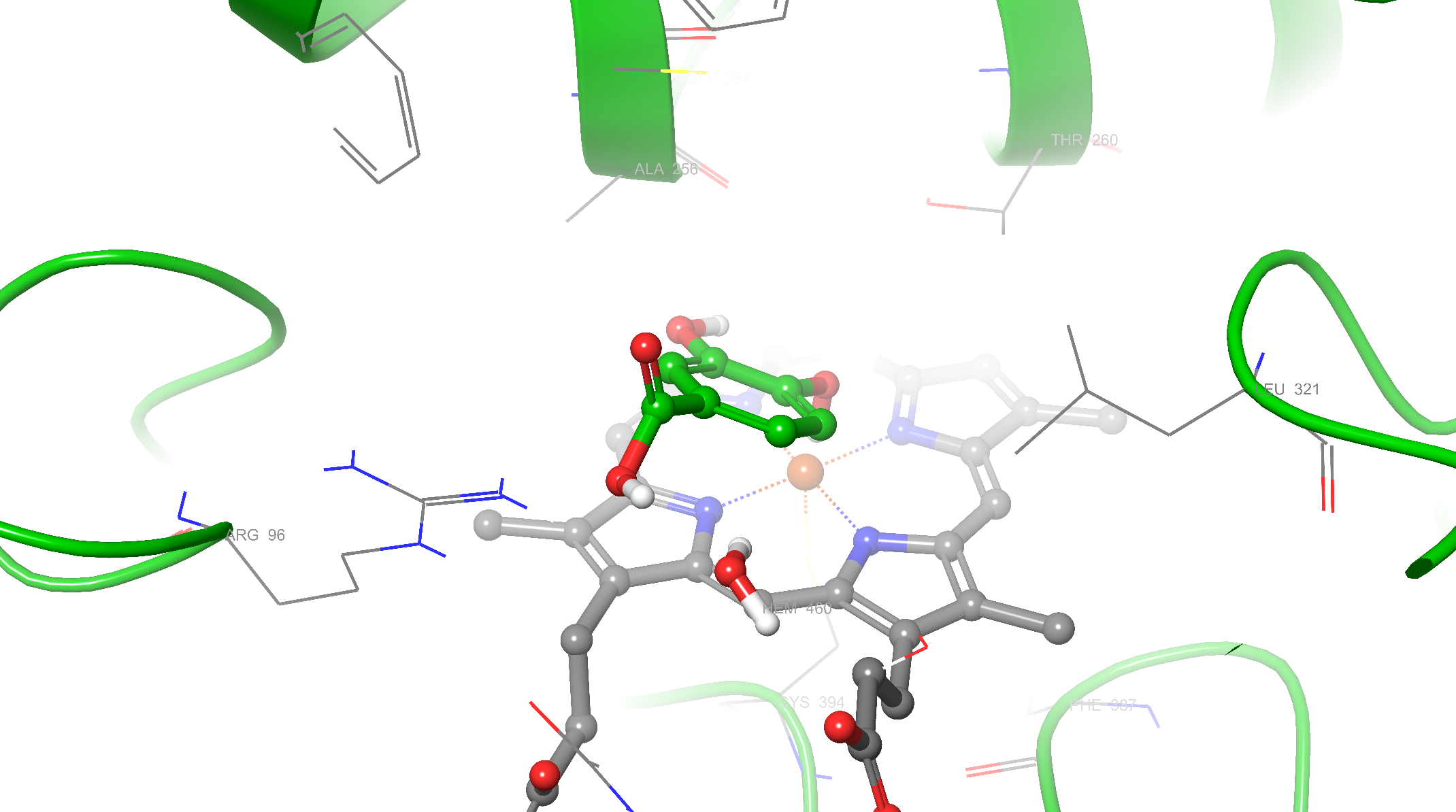


**Figure 6S.** 3D interaction diagram with 1EA1 for protocatechuic acid.

**
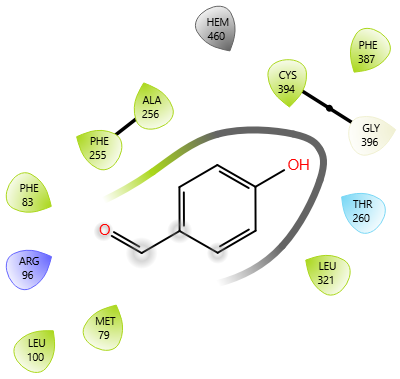
**

**Figure 7S.** 2D interaction diagram with 1EA1 for hydroxybenzaldeyde.


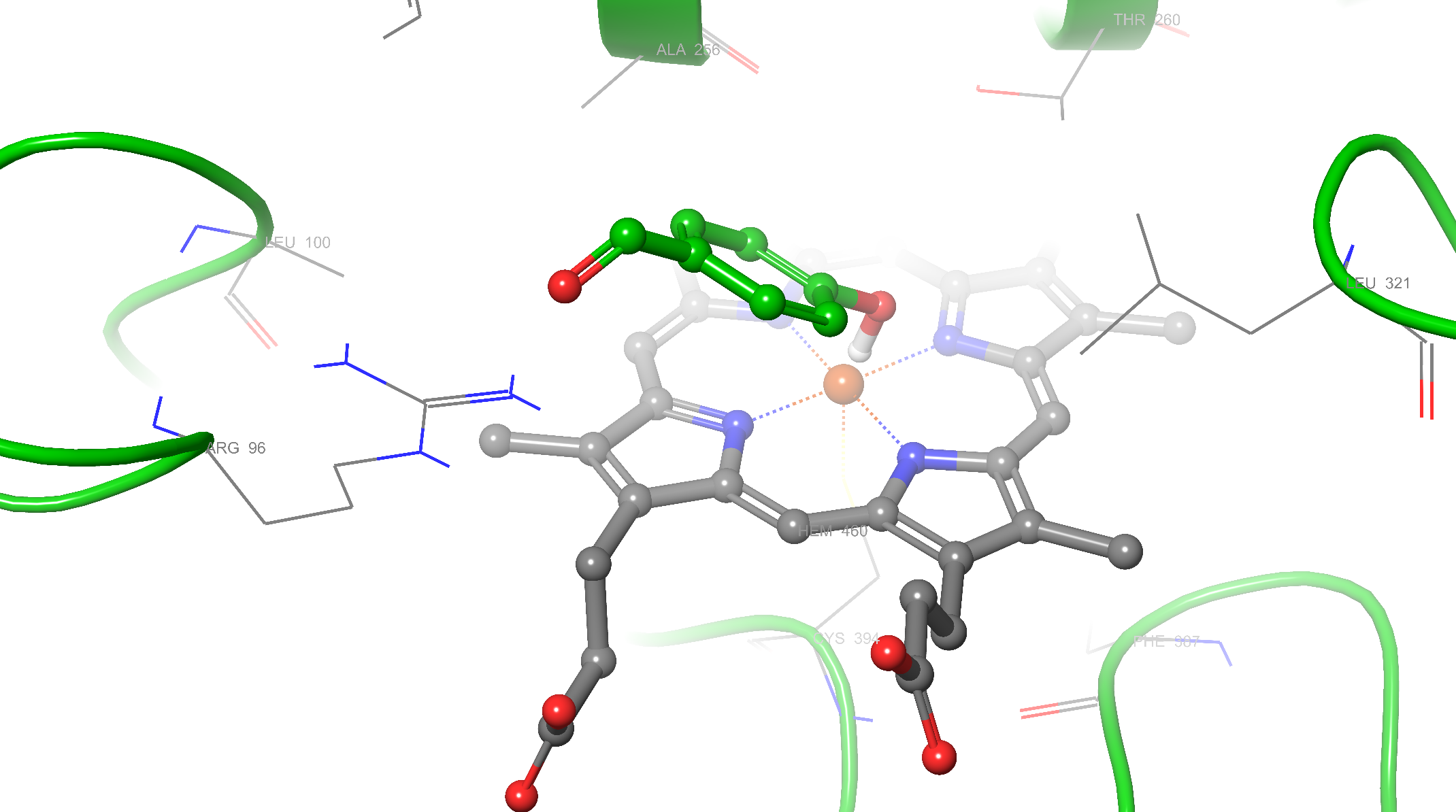


**Figure 8S.** 3D interaction diagram with 1EA1 for hydroxybenzaldeyde.

**
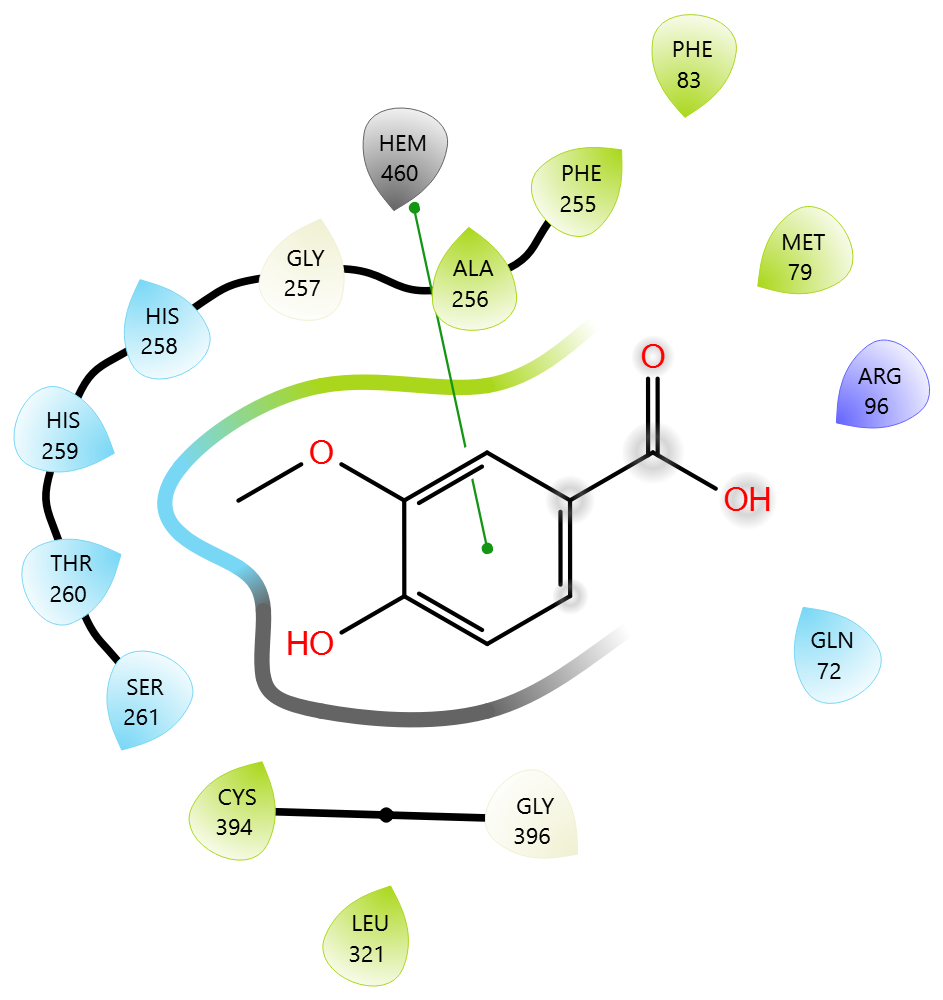
**

**Figure 9S.** 2D interaction diagram with 1EA1 for vanillic acid.


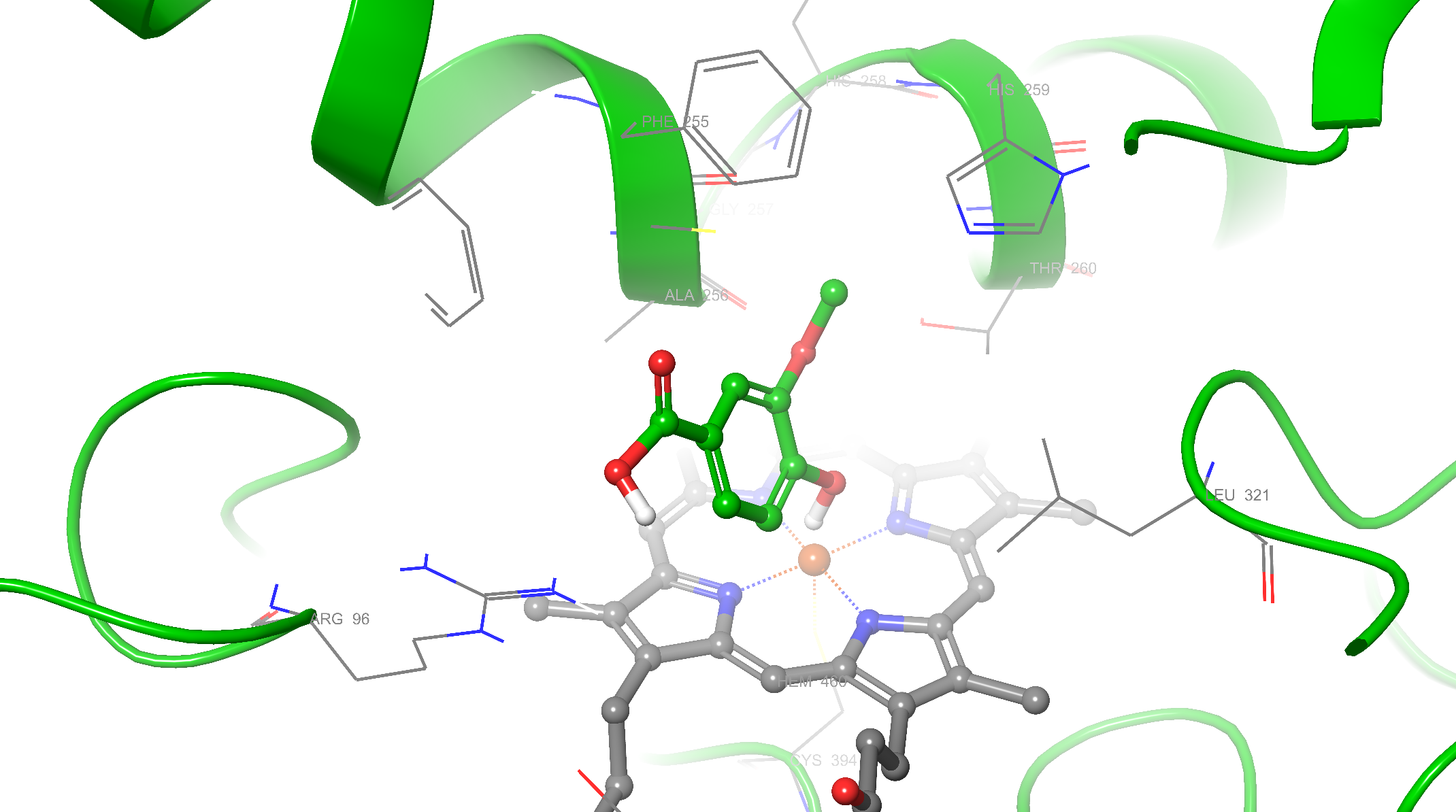


**Figure 10S.** 3D interaction diagram with 1EA1 for vanillic acid.

**
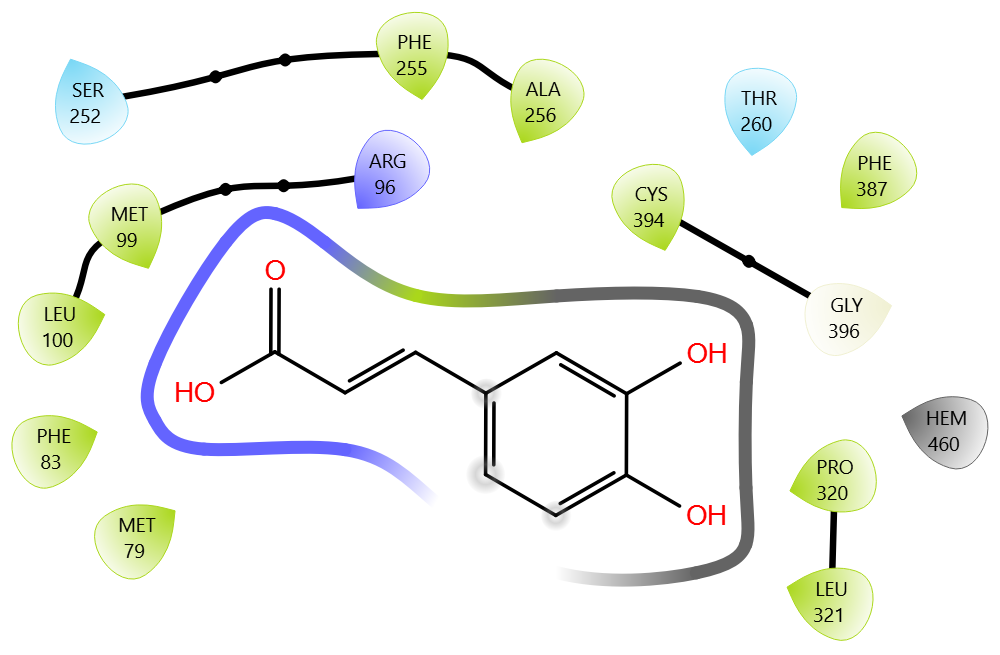
**

**Figure 11S.** 2D interaction diagram with 1EA1 for caffeic acid.


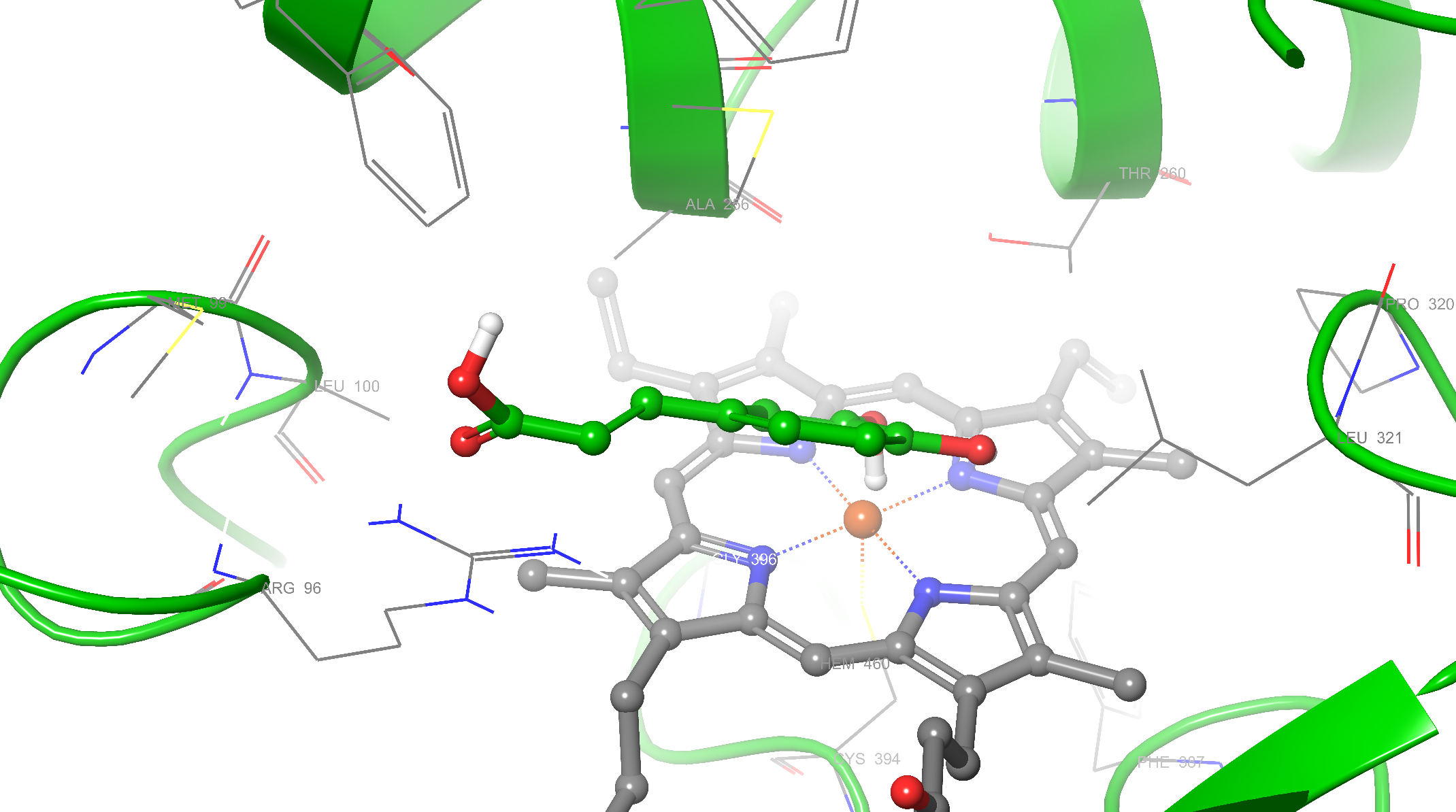


**Figure 12S.** 3D interaction diagram with 1EA1 for caffeic acid.

**
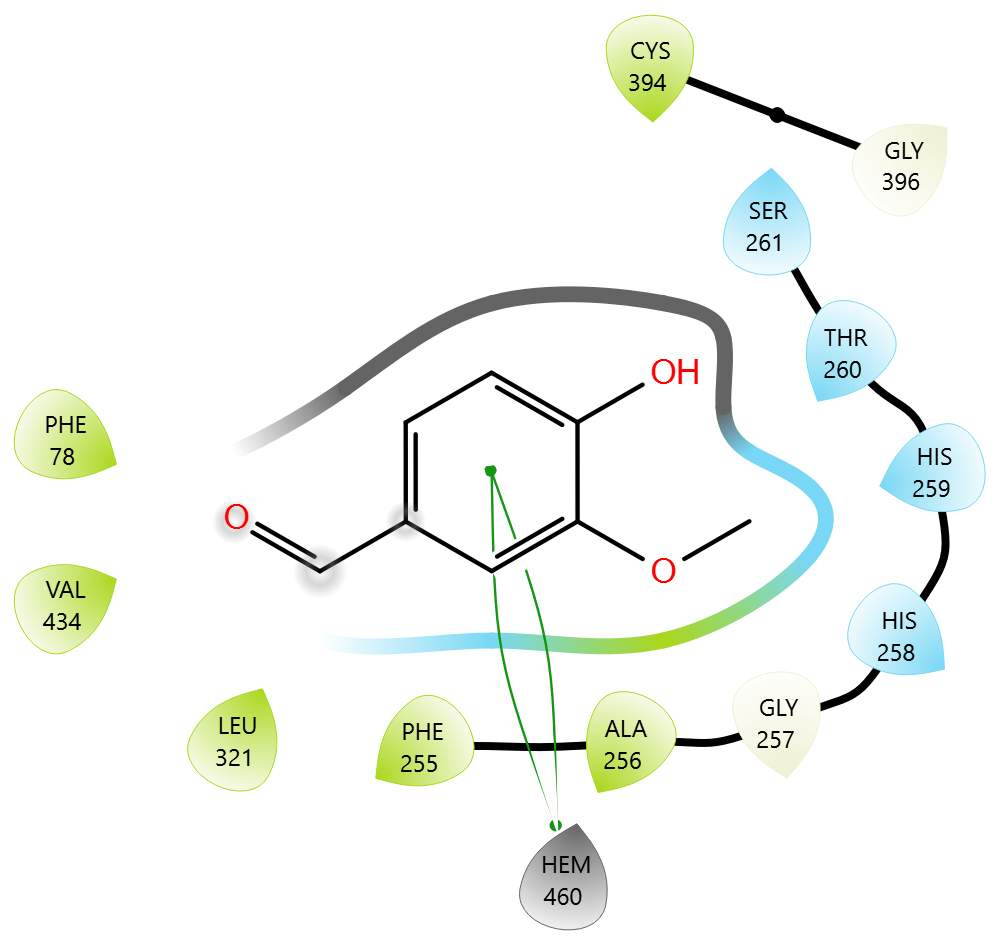
**

**Figure 13S.** 2D interaction diagram with 1EA1 for vanillin.


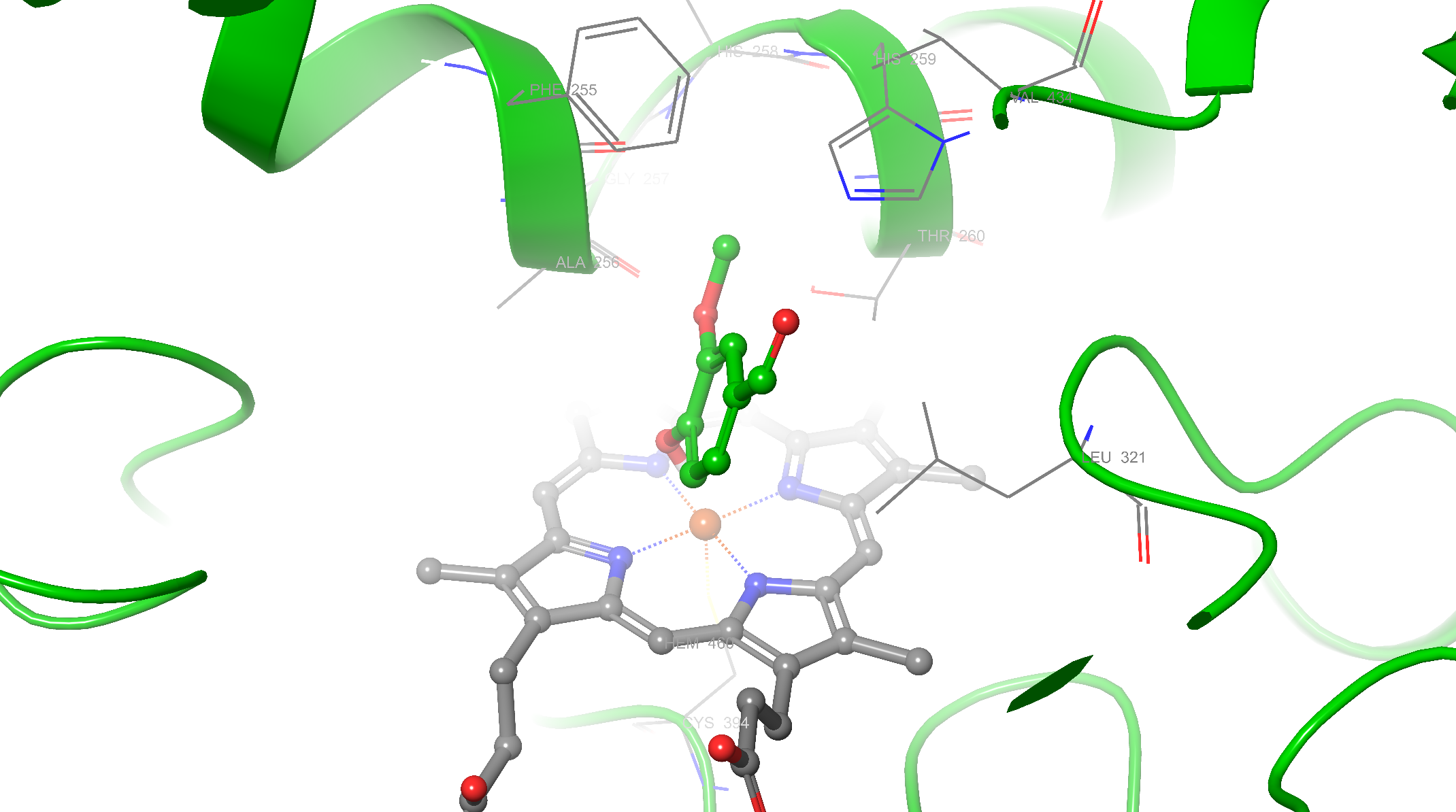


**Figure 14S.** 3D interaction diagram with 1EA1 for vanillin.

**
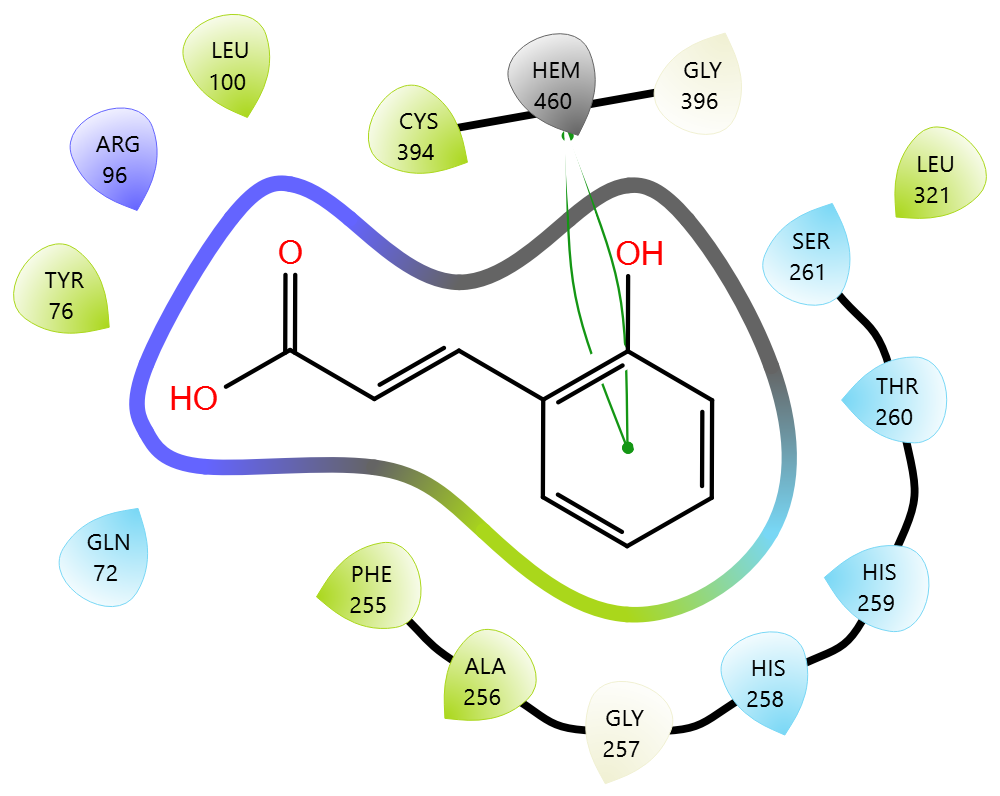
**

**Figure 15S.** 2D interaction diagram with 1EA1 for *o*-coumaric acid.


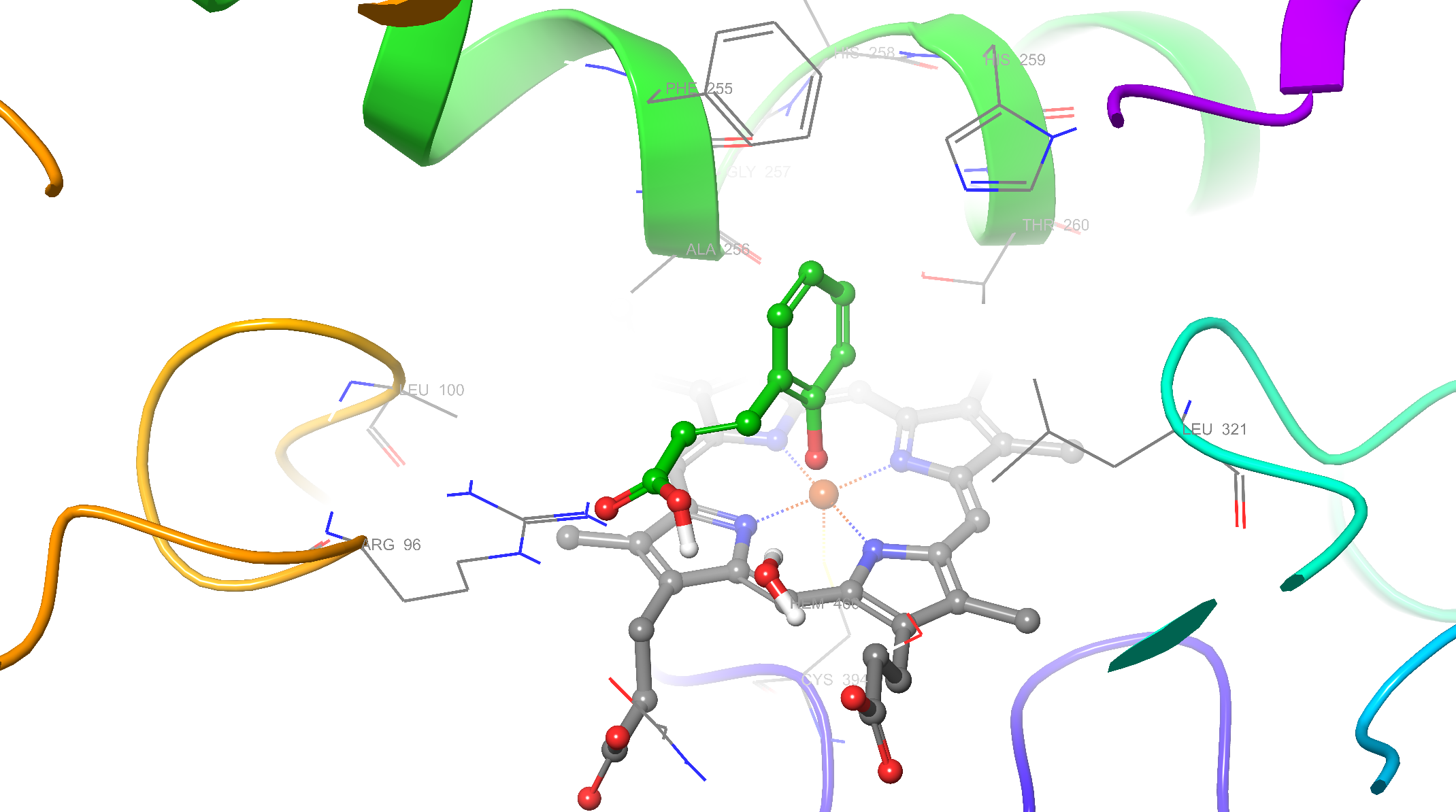


**Figure 16S.** 3D interaction diagram with 1EA1 for *o*-coumaric acid.

**
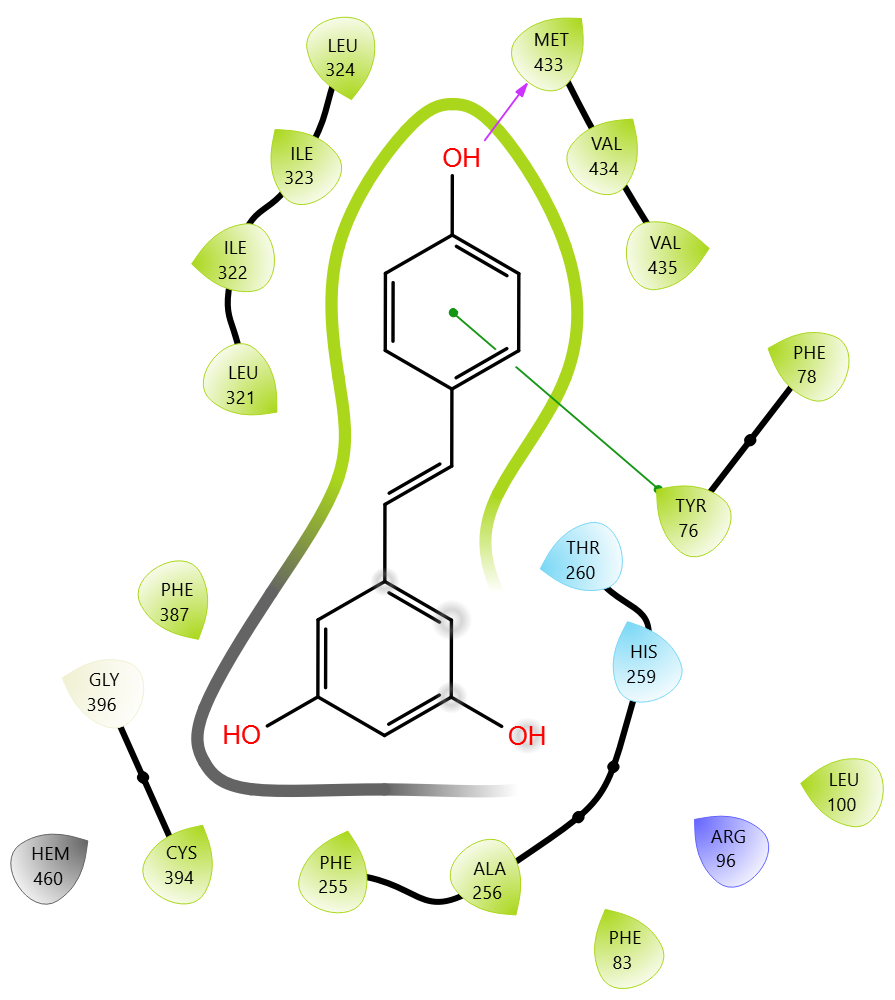
**

**Figure 17S.** 2D interaction diagram with 1EA1 for resveratrol.


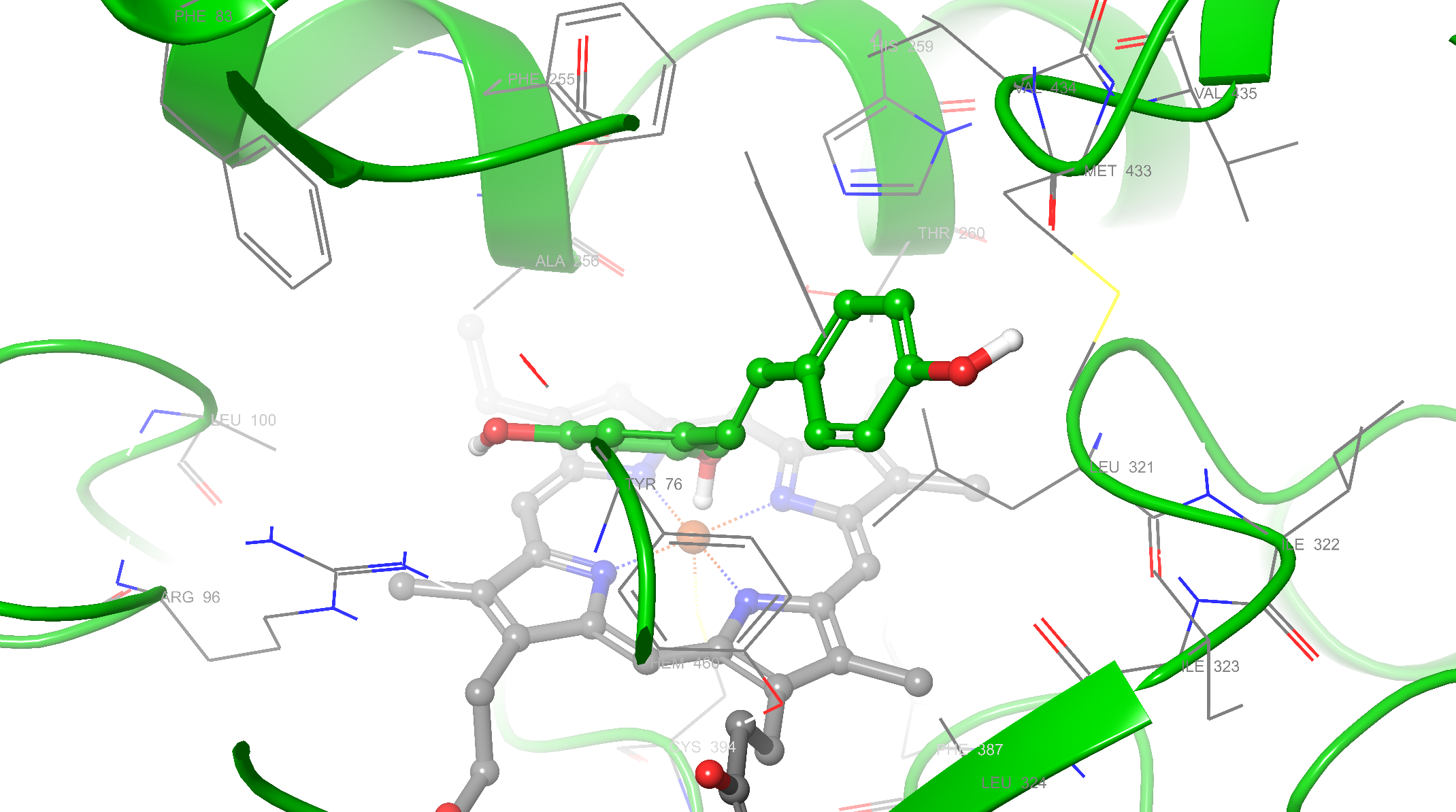


**Figure 18S.** 3D interaction diagram with 1EA1 for resveratrol.

**
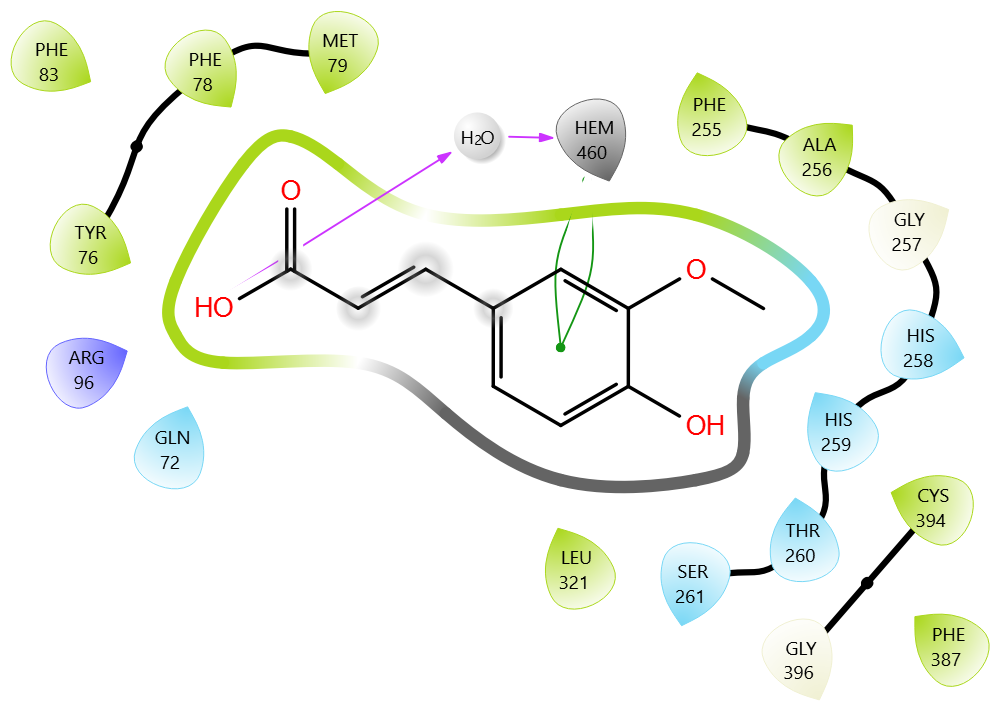
**

**Figure 19S.** 2D interaction diagram with 1EA1 for *trans*-ferulic acid.


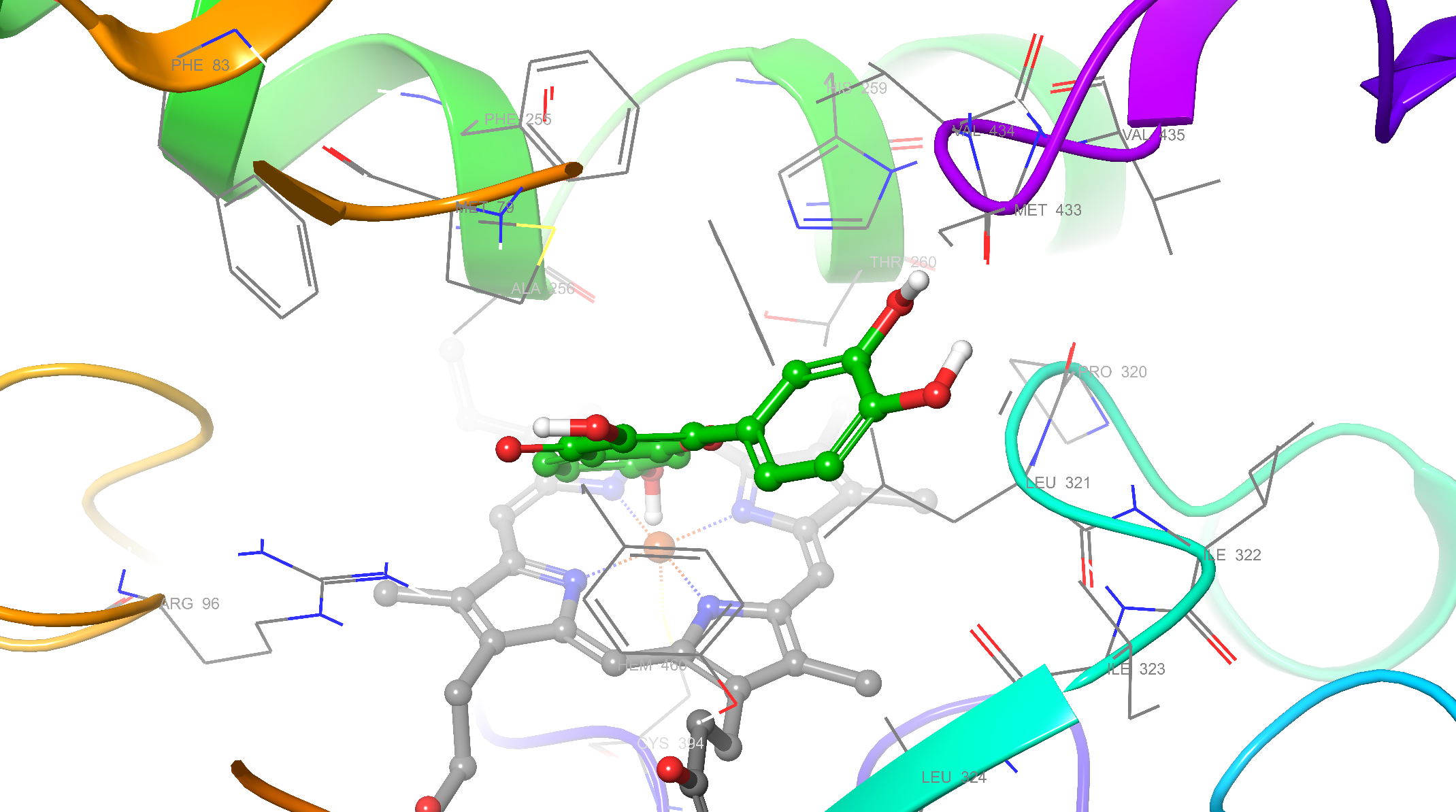


**Figure 20S.** 3D interaction diagram with 1EA1 for *trans*-ferulic acid.

**
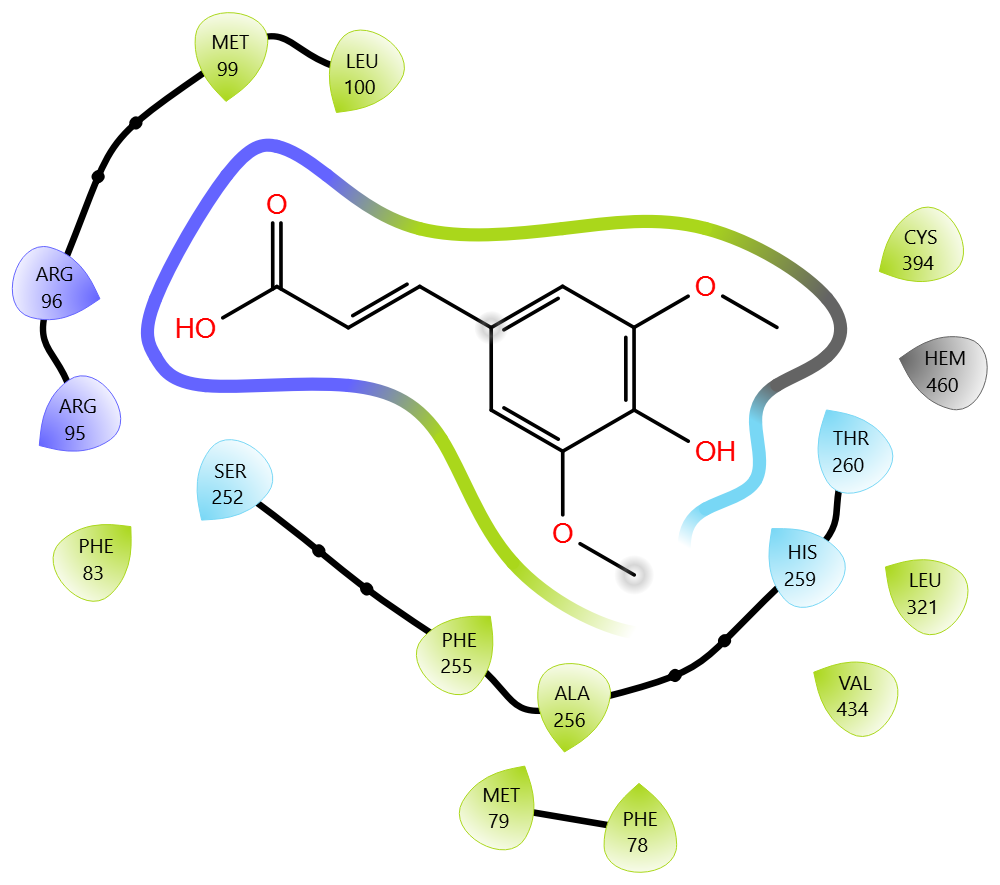
**

**Figure 21S.** 2D interaction diagram with 1EA1 for sinapic acid.

**
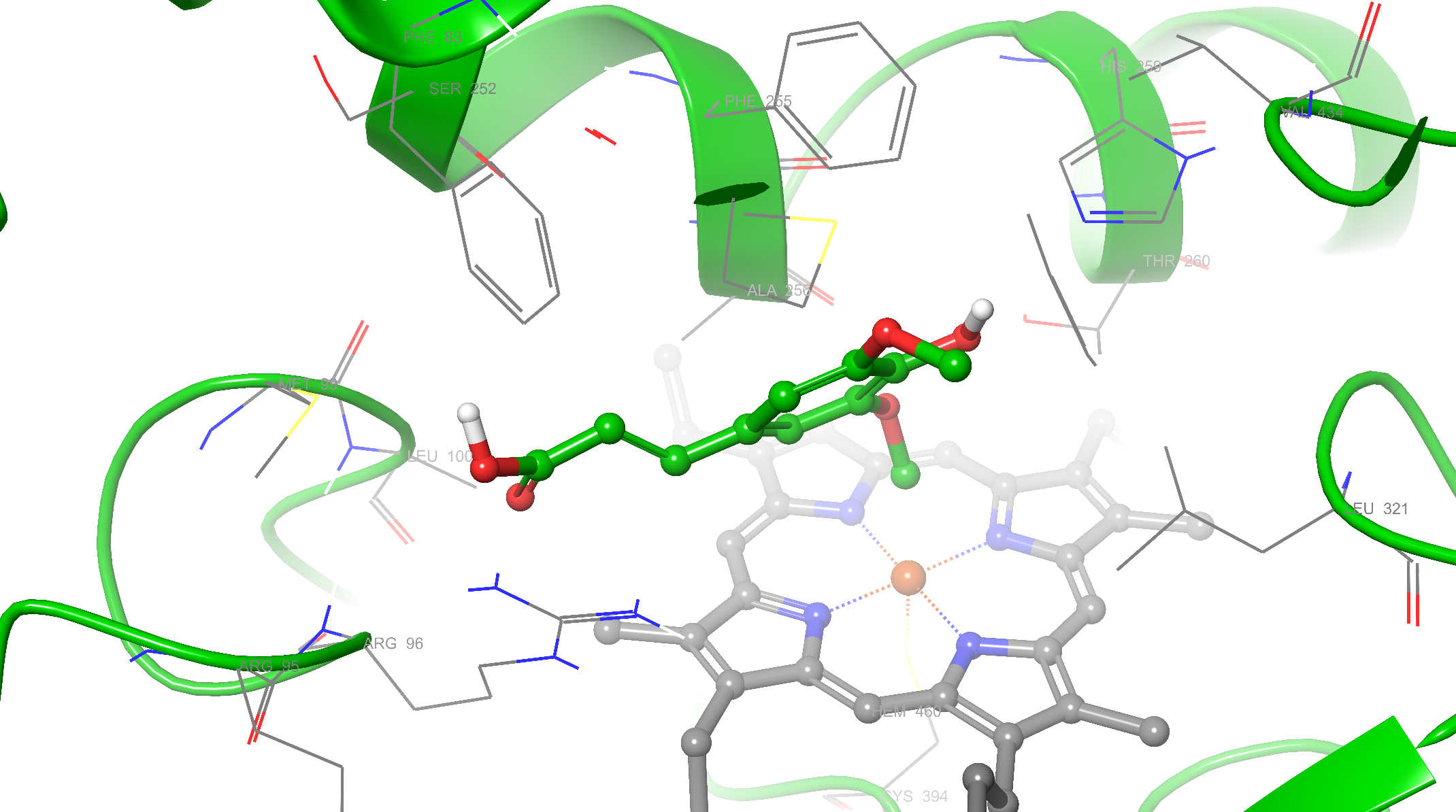
**

**Figure 22S.** 3D interaction diagram with 1EA1 for sinapic acid.

**
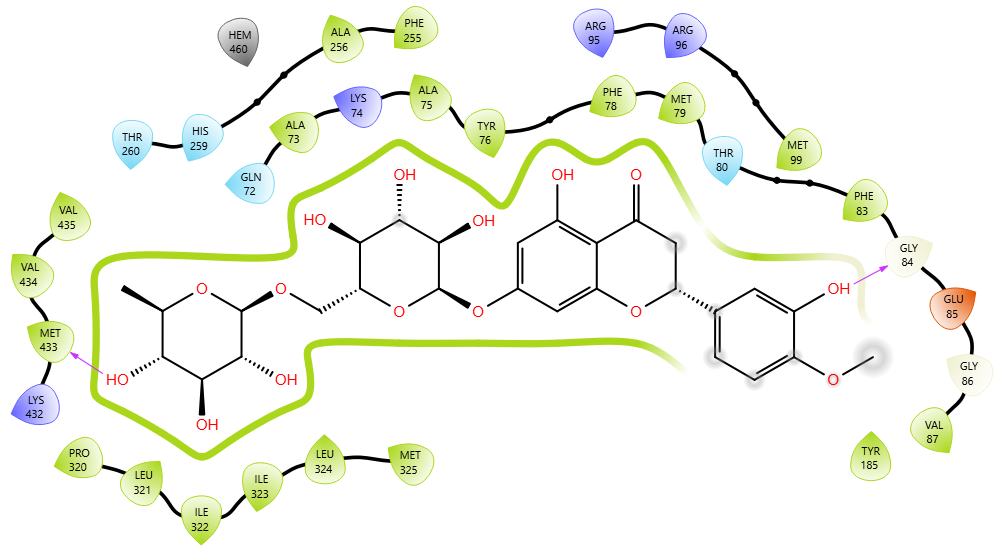
**

**Figure 23S.** 2D interaction diagram with 1EA1 for hesperidin.


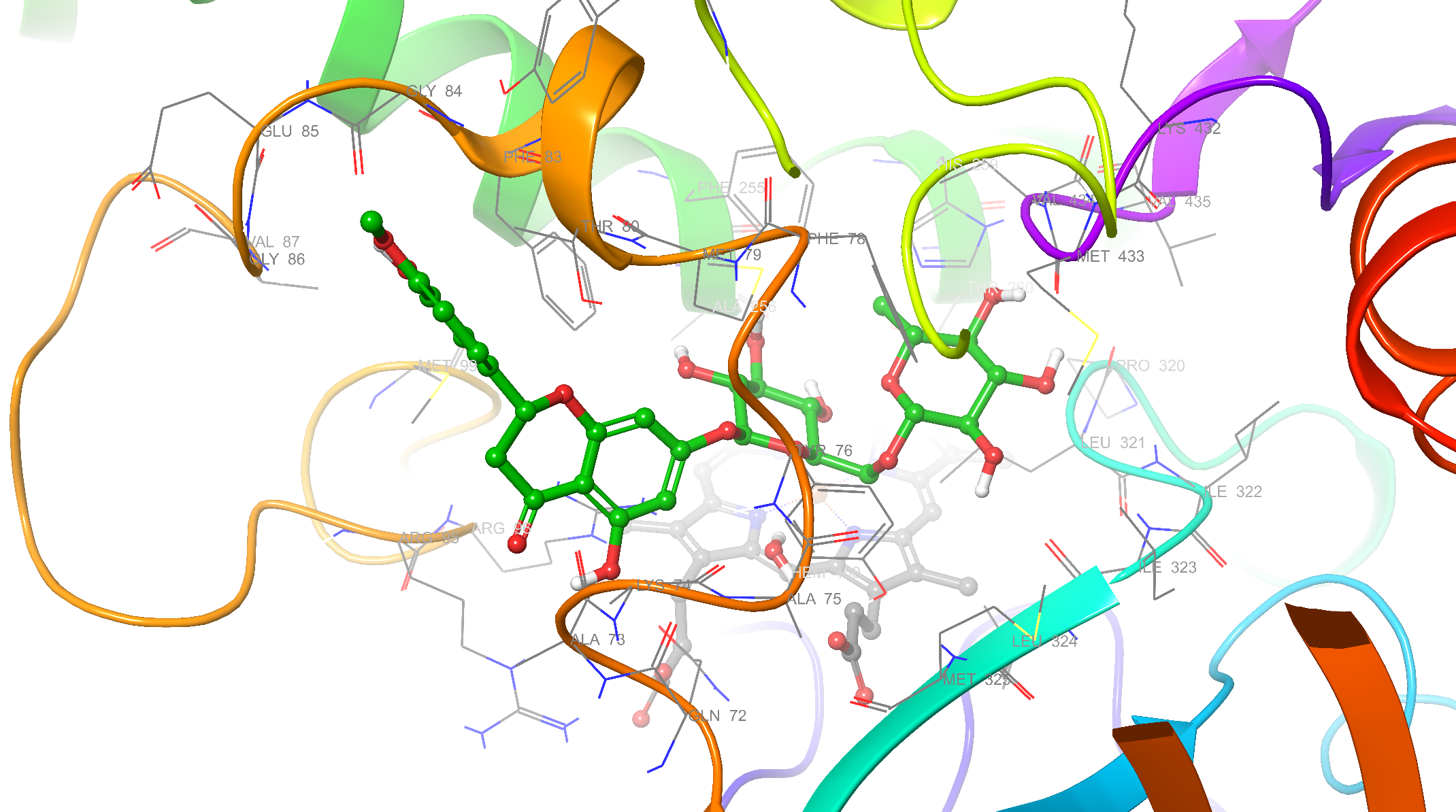


**Figure 24S.** 3D interaction diagram with 1EA1 for hesperidin.

**
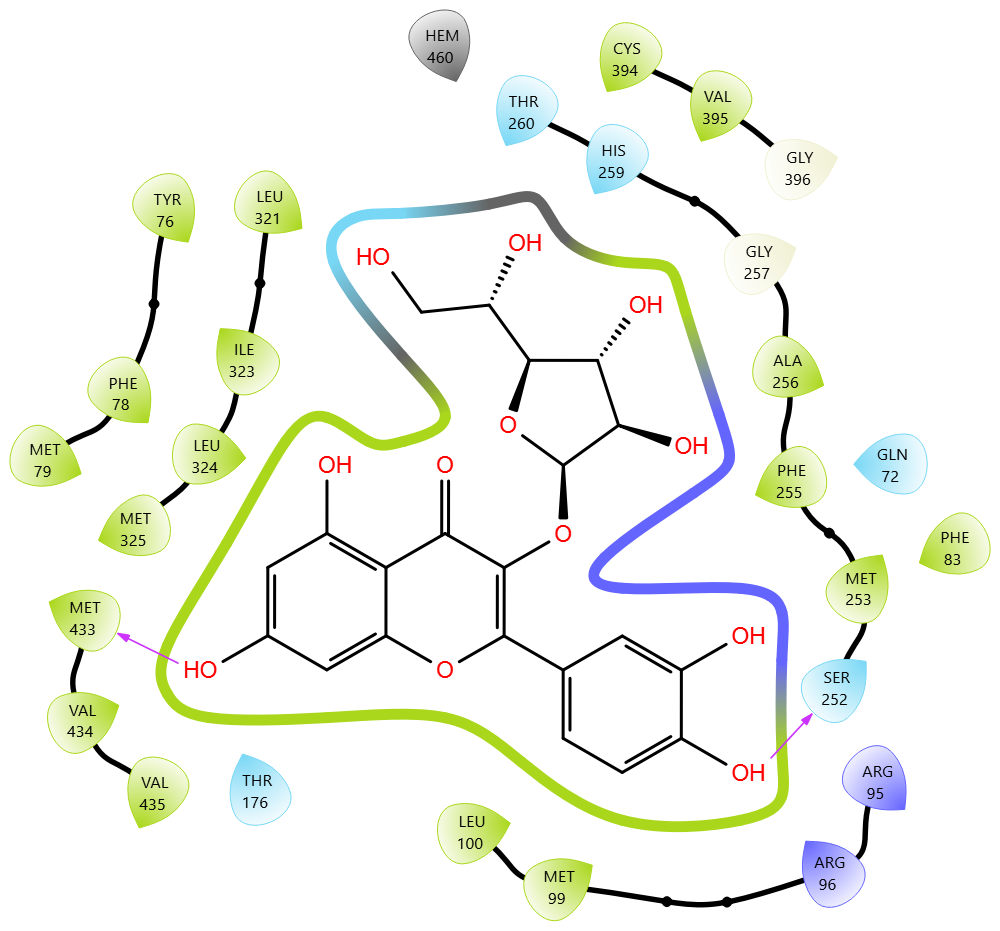
**

**Figure 25S.** 2D interaction diagram with 1EA1 for isoquercitrin.


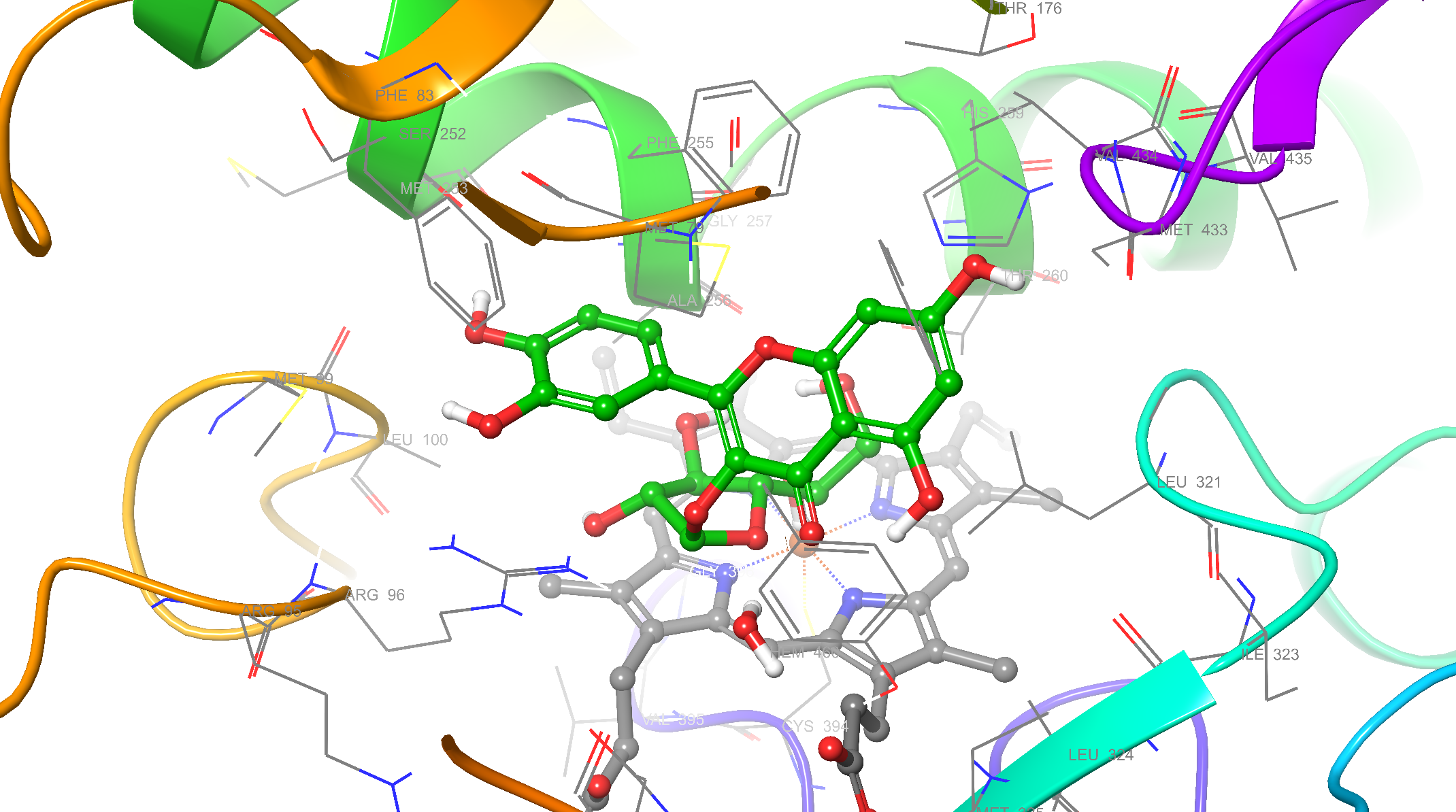


**Figure 26S.** 3D interaction diagram with 1EA1 for isoquercitrin.

**
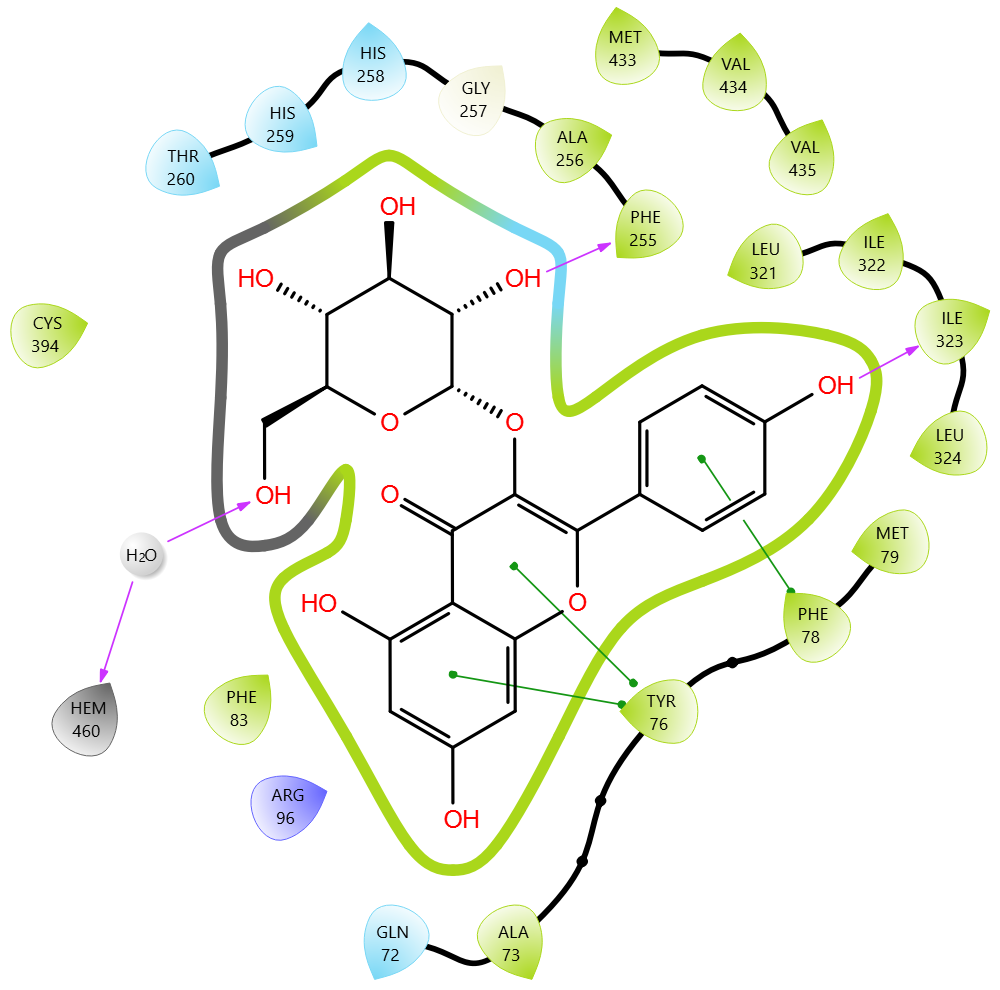
**

**Figure 27S.** 2D interaction diagram with 1EA1 for kaempferol-3-glucoside.


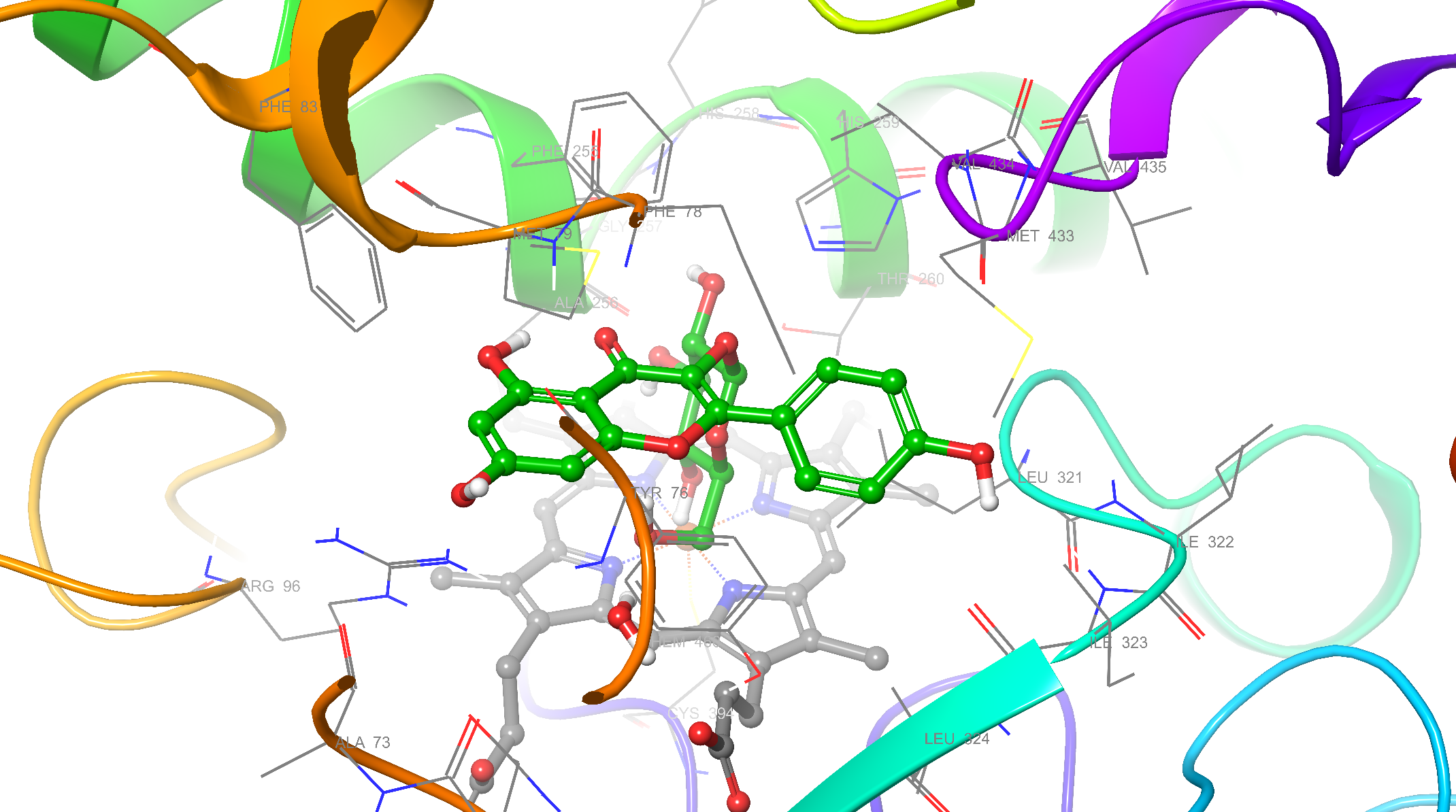


**Figure 28S.** 3D interaction diagram with 1EA1 for kaempferol-3-glucoside.

**
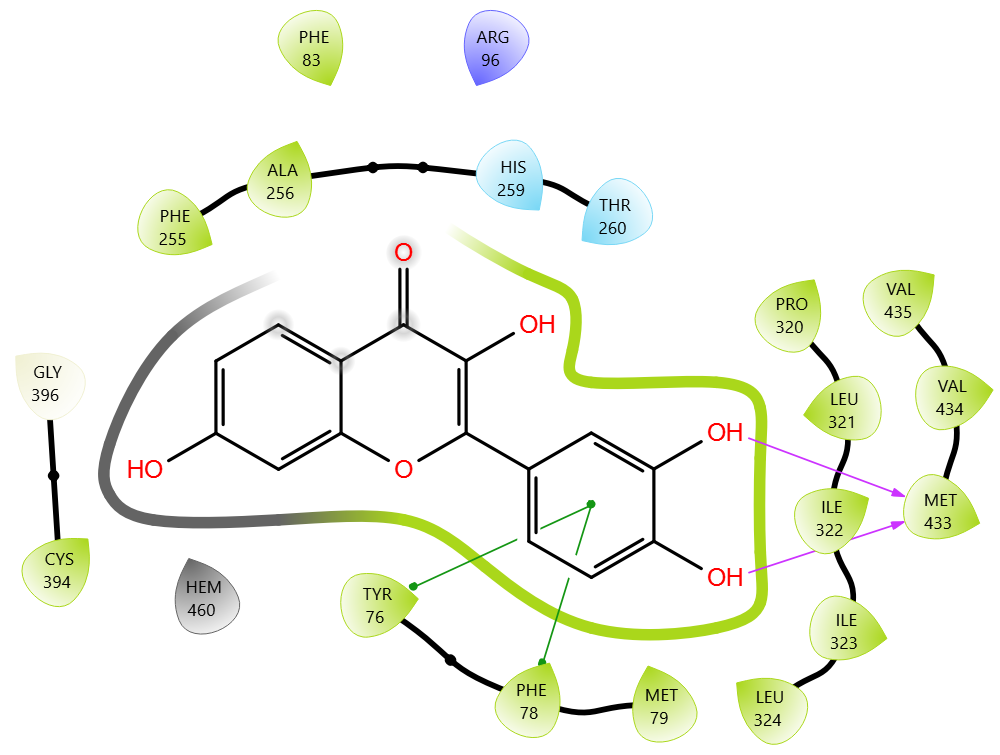
**

**Figure 29S.** 2D interaction diagram with 1EA1 for fisetin.


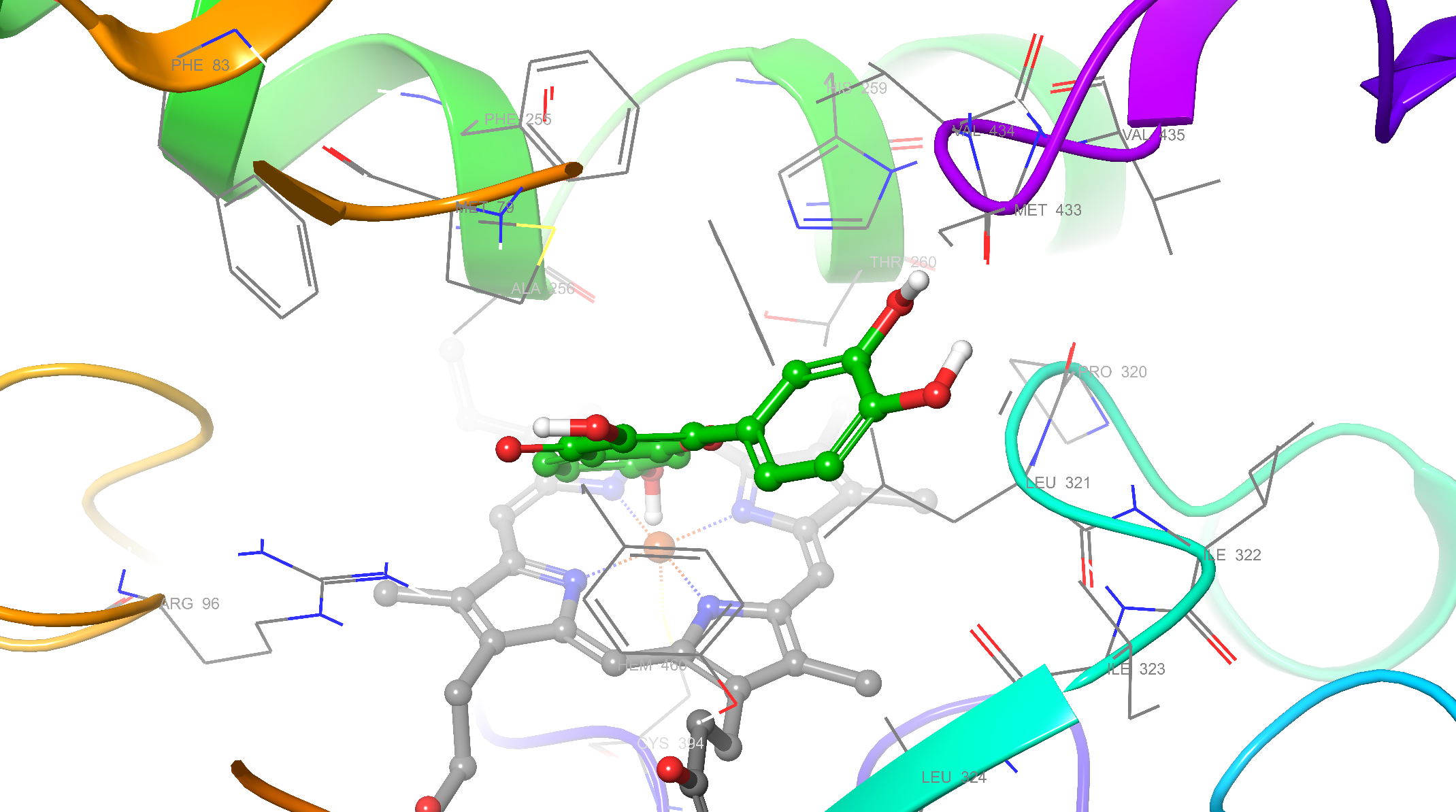


**Figure 30S.** 3D interaction diagram with 1EA1 for fisetin.

**
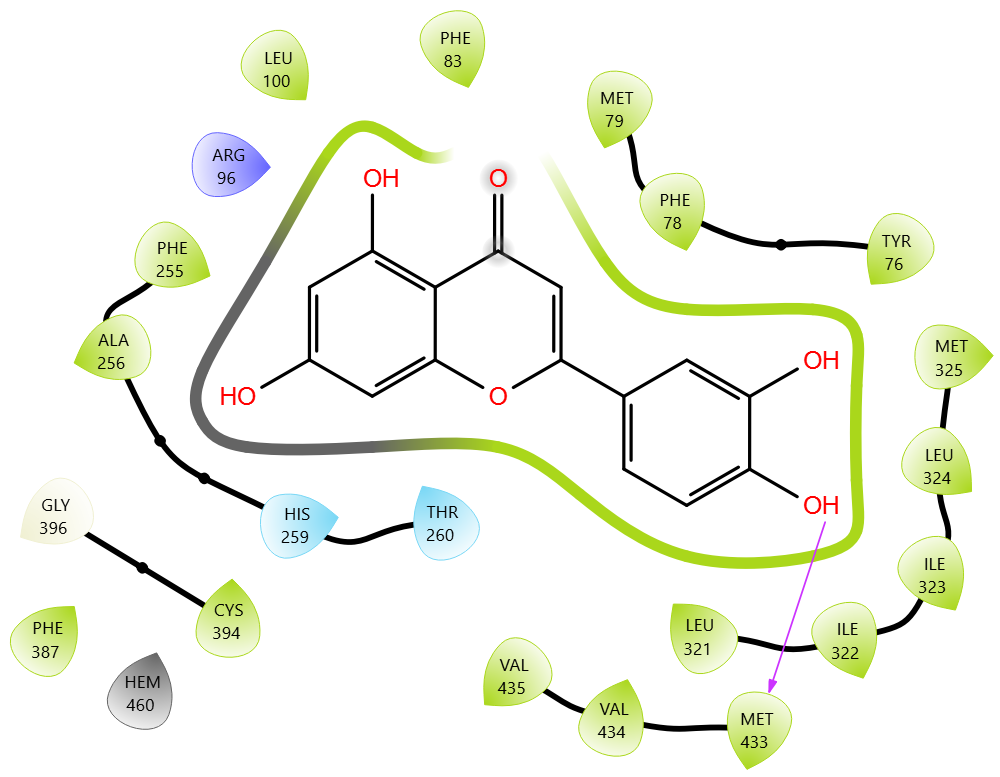
**

**Figure 31S.** 2D interaction diagram with 1EA1 for luteolin.


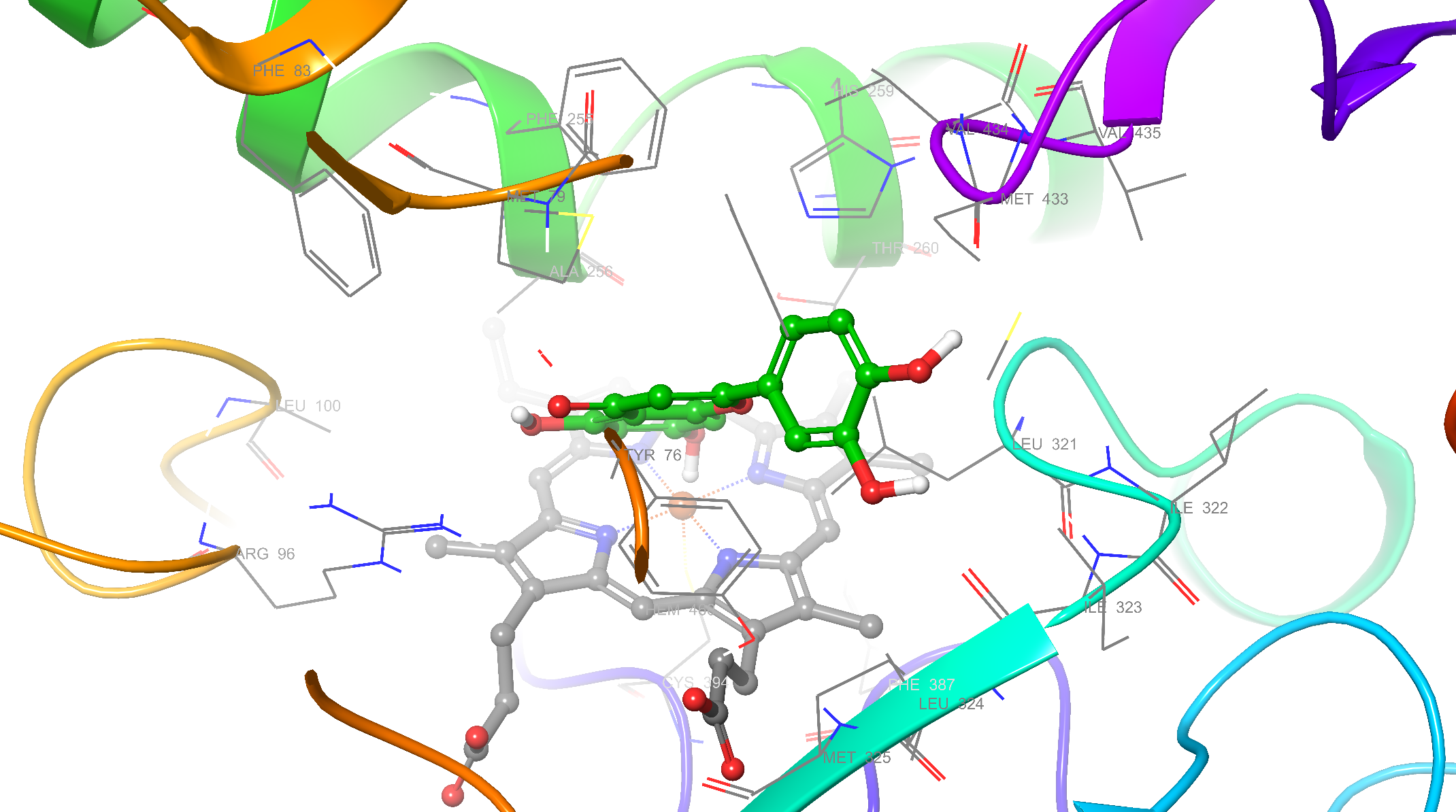


**Figure 32S.** 3D interaction diagram with 1EA1 for luteolin.

**
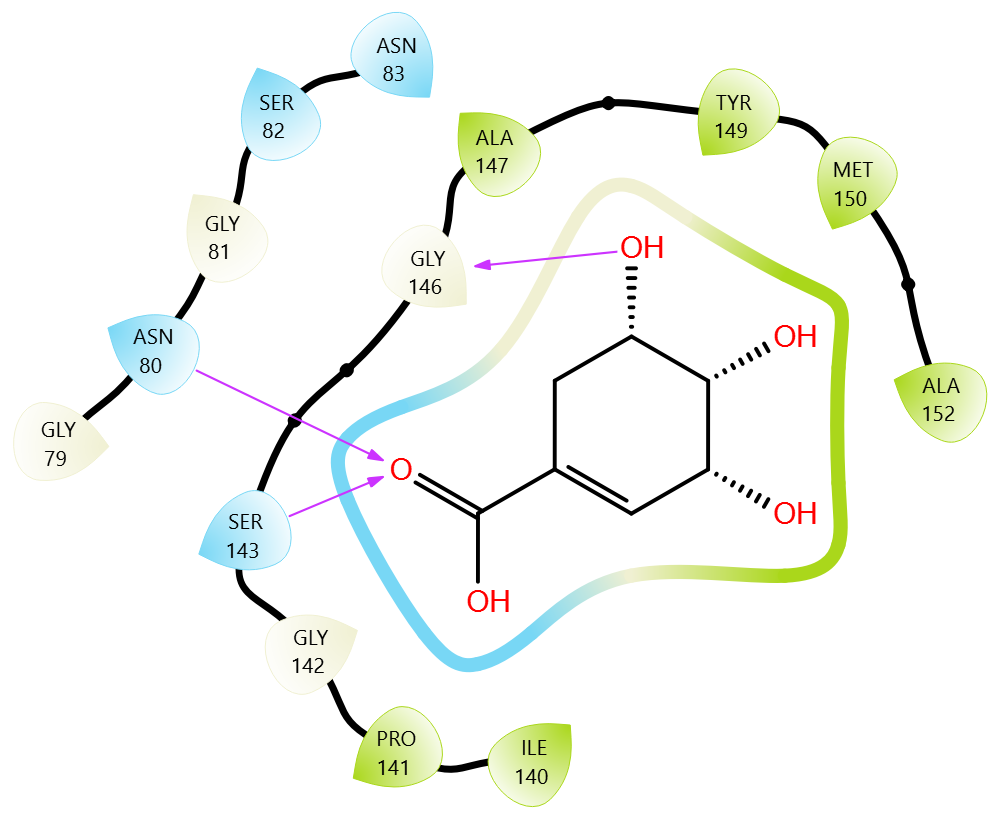
**

**Figure 33S.** 2D interaction diagram with 1HSK for shikimic acid.


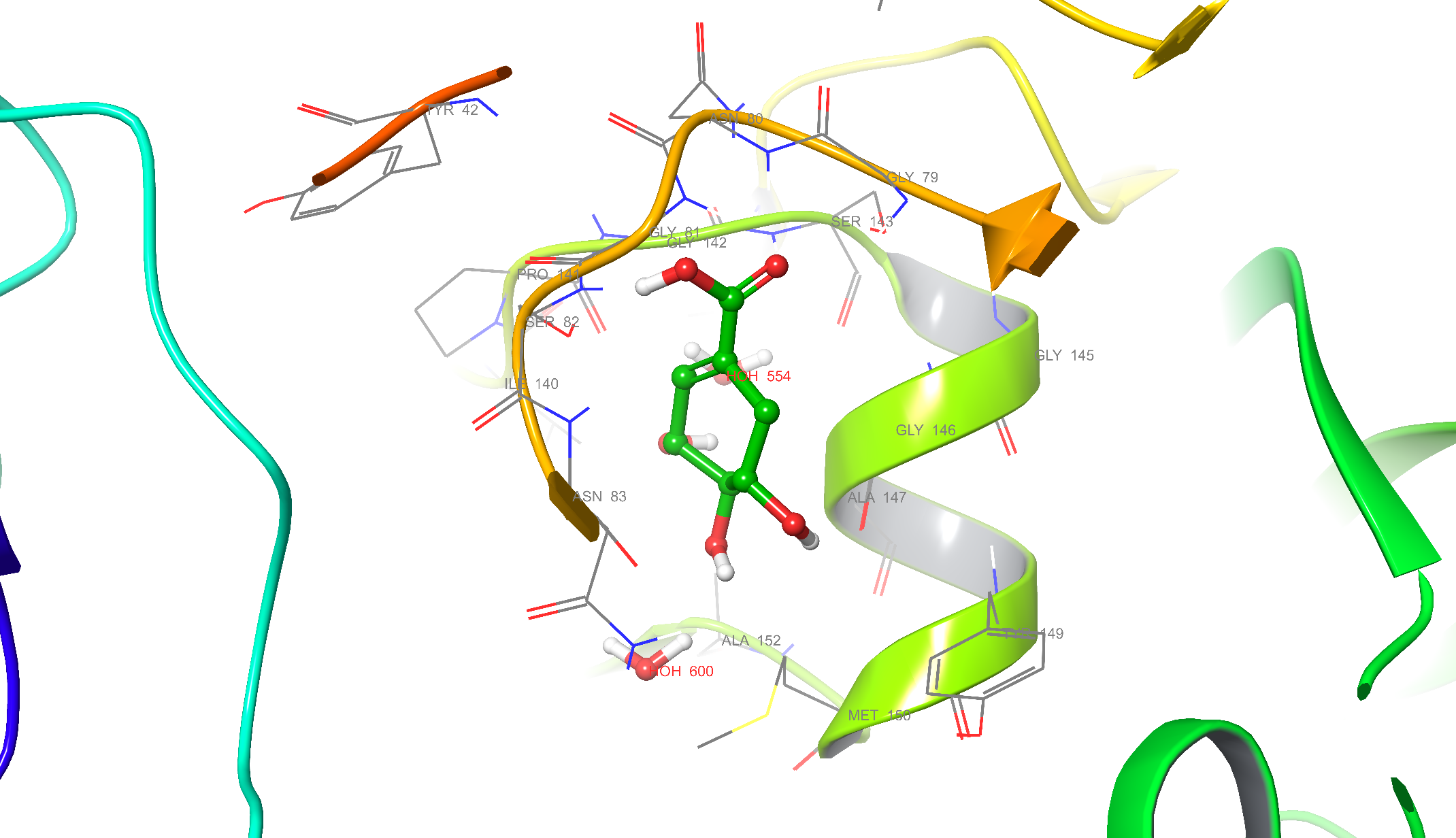


**Figure 34S.** 3D interaction diagram with 1HSK for shikimic acid.

**
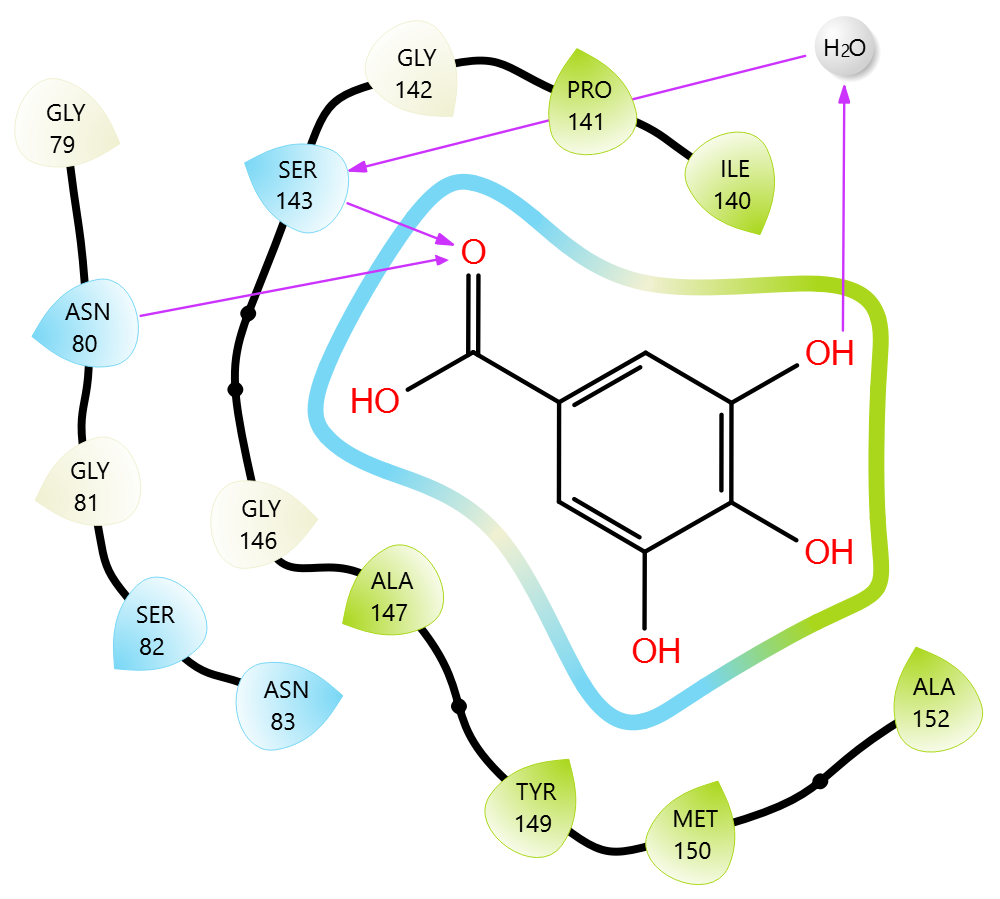
**

**Figure 3S5.** 2D interaction diagram with 1HSK for gallic acid.


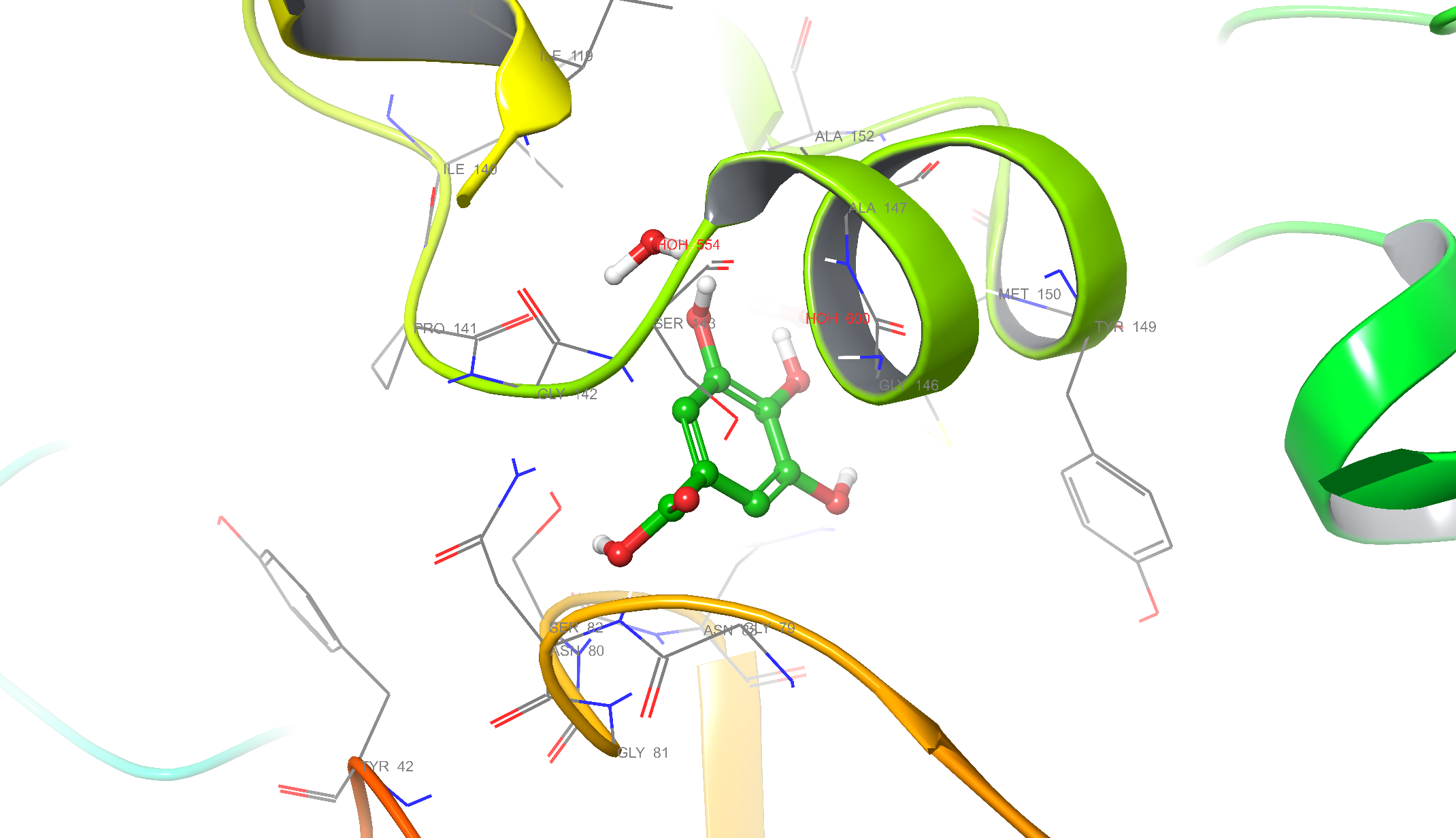


**Figure 36S.** 3D interaction diagram with 1HSK for gallic acid.

**
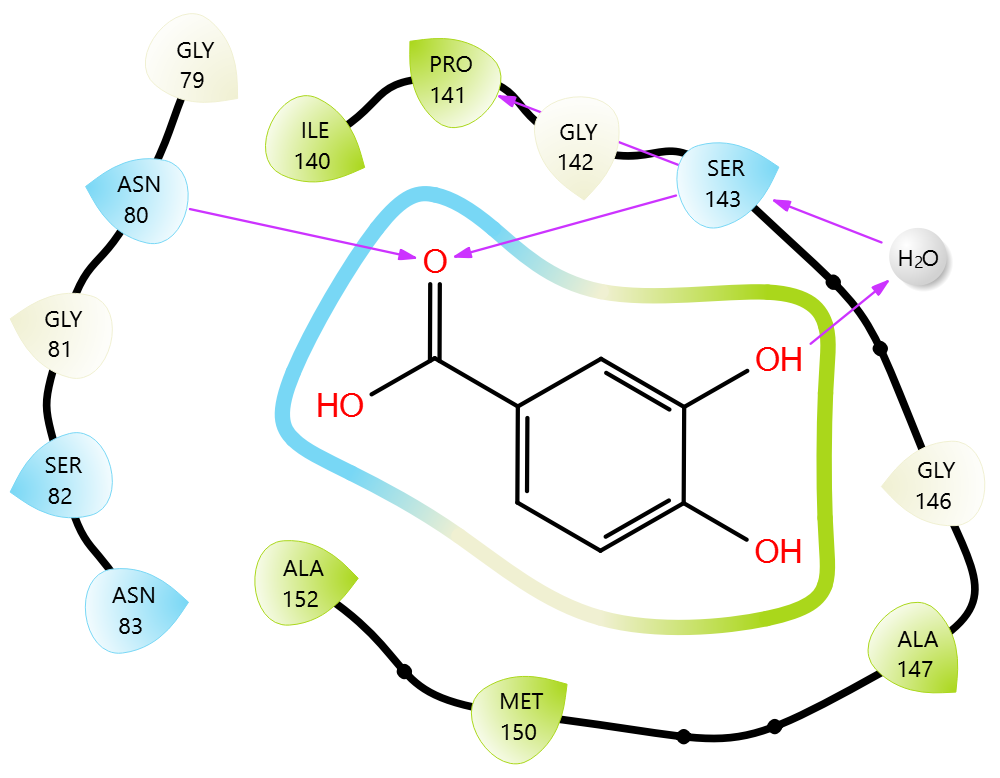
**

**Figure 37S.** 2D interaction diagram with 1HSK for protocatechuic acid.


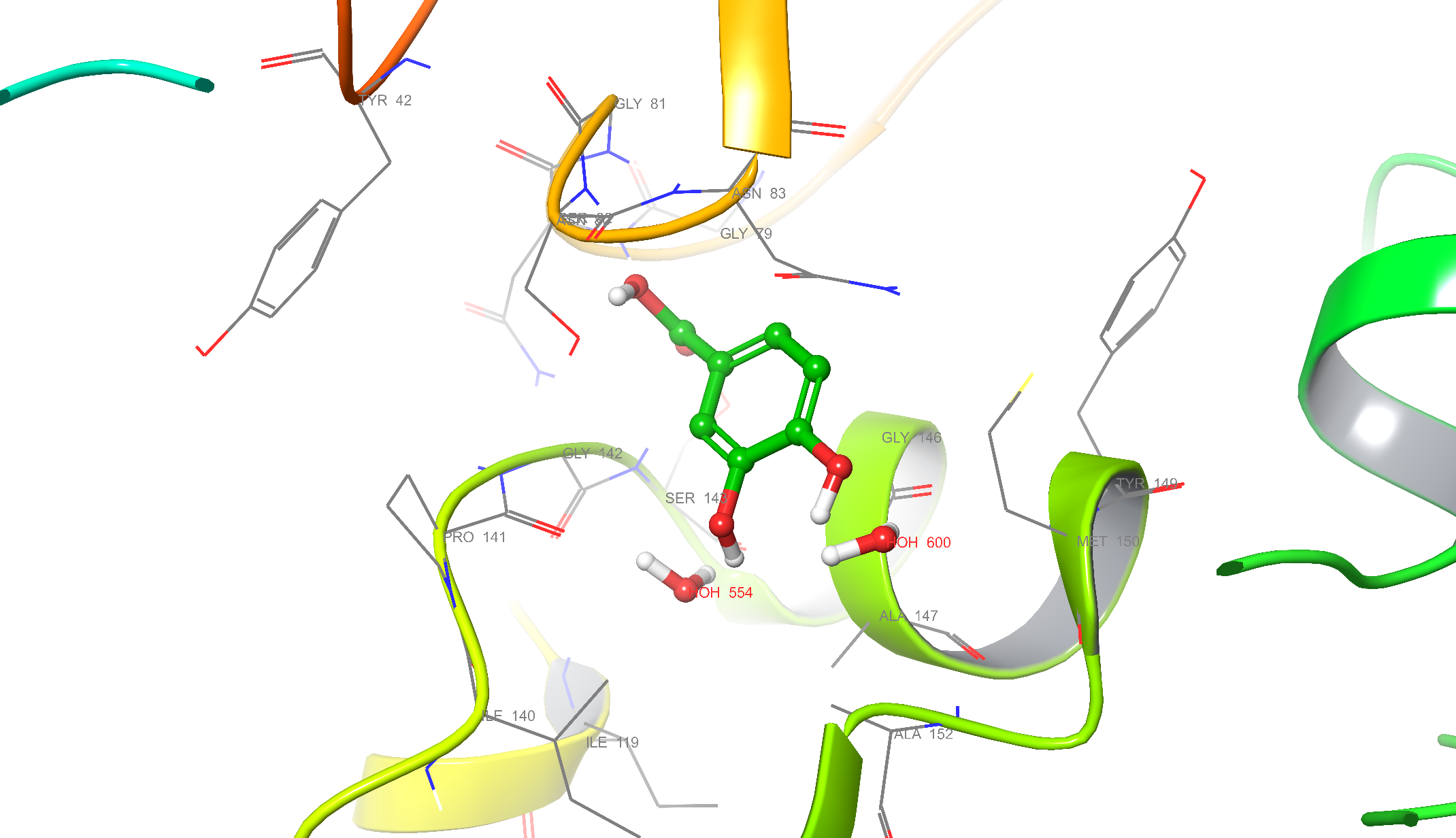


**Figure 38S.** 3D interaction diagram with 1HSK for protocatechuic acid.

**
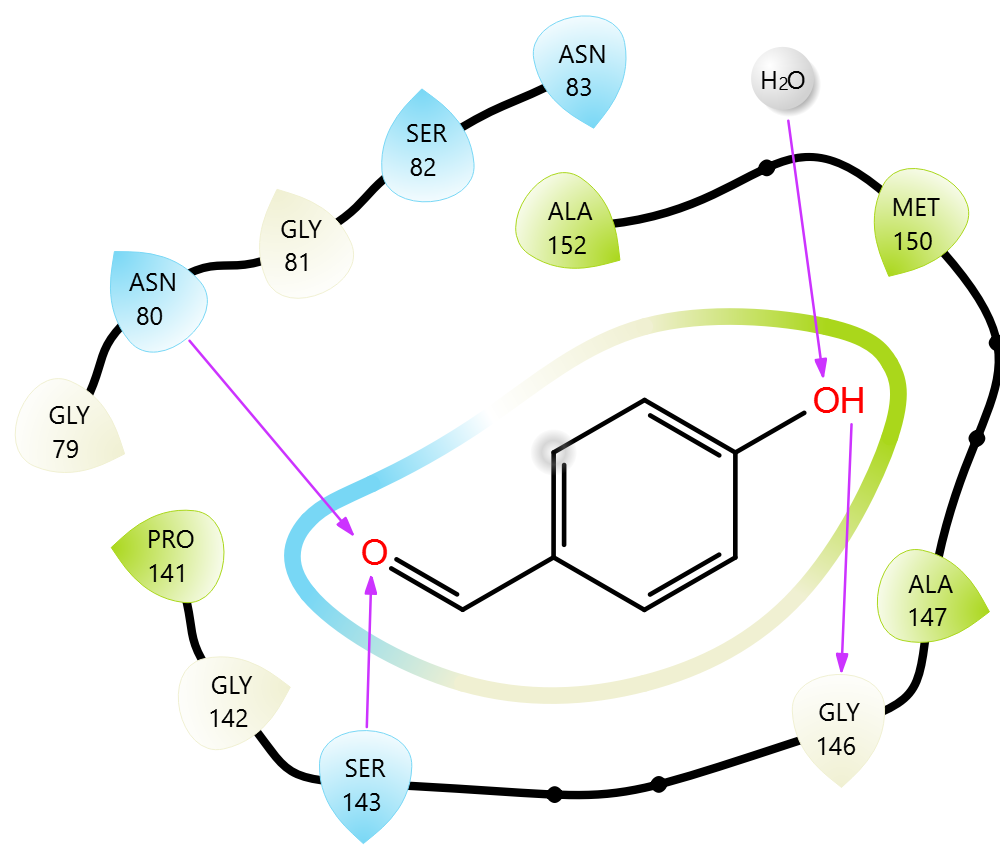
**

**Figure 39S.** 2D interaction diagram with 1HSK for hydroxybenzaldeyde.


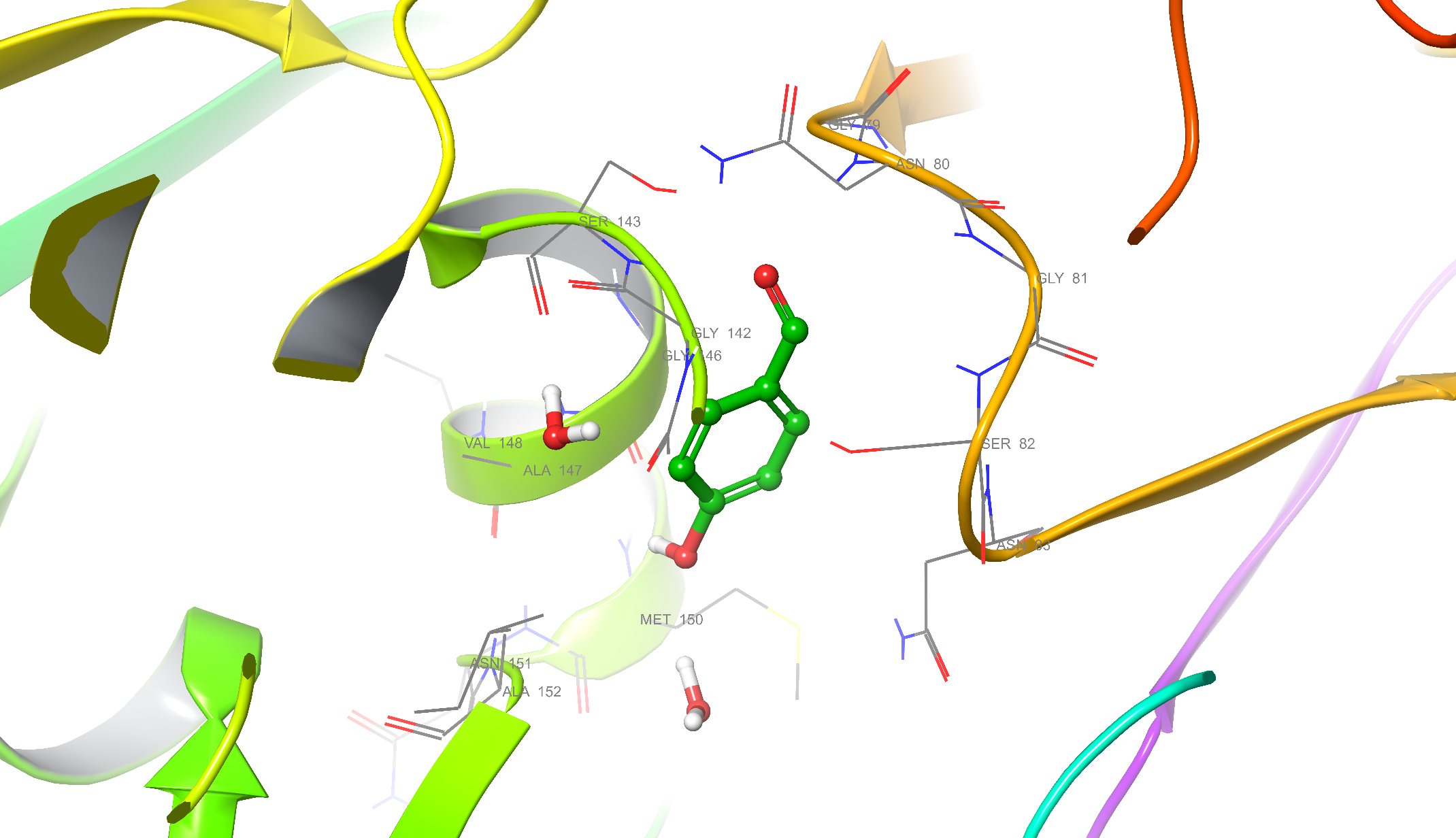


**Figure 40S.** 3D interaction diagram with 1HSK for hydroxybenzaldeyde.

**
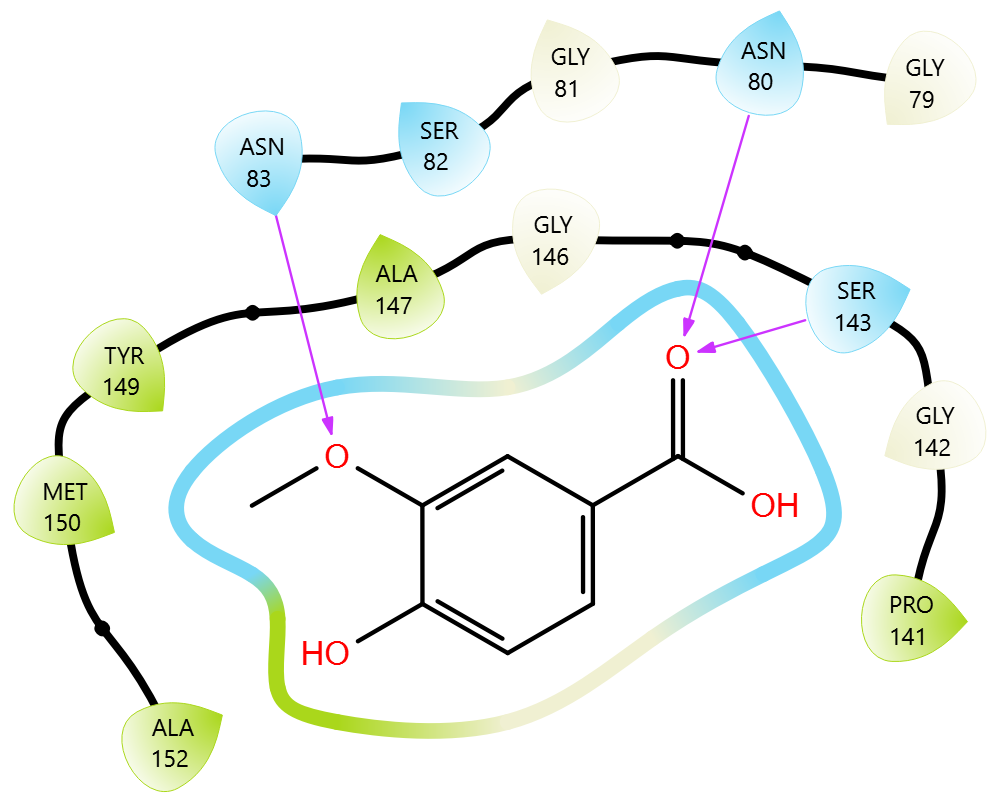
**

**Figure 41S.** 2D interaction diagram with 1HSK for vanillic acid.


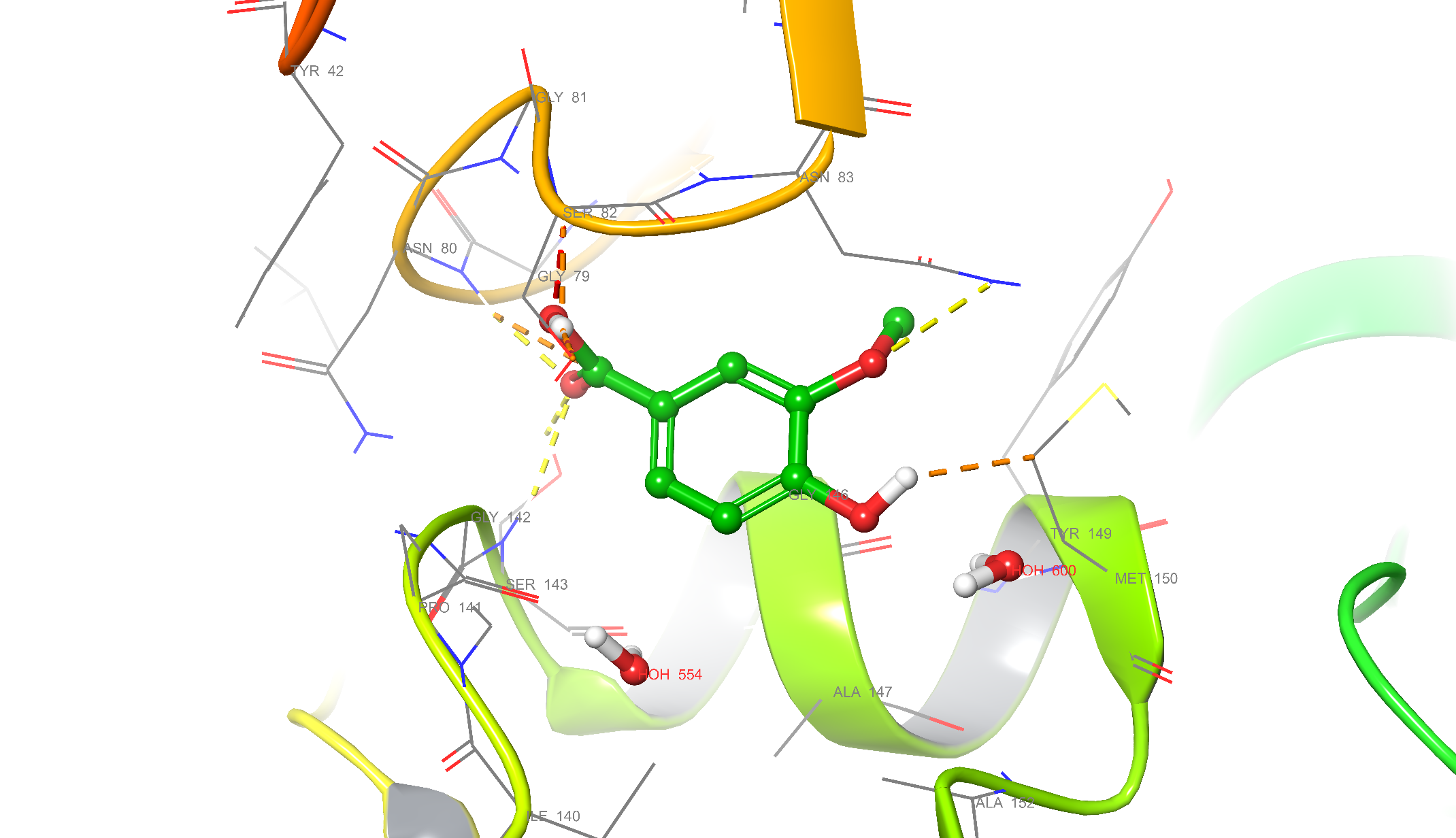


**Figure 42S.** 3D interaction diagram with 1HSK for vanillic acid.

**
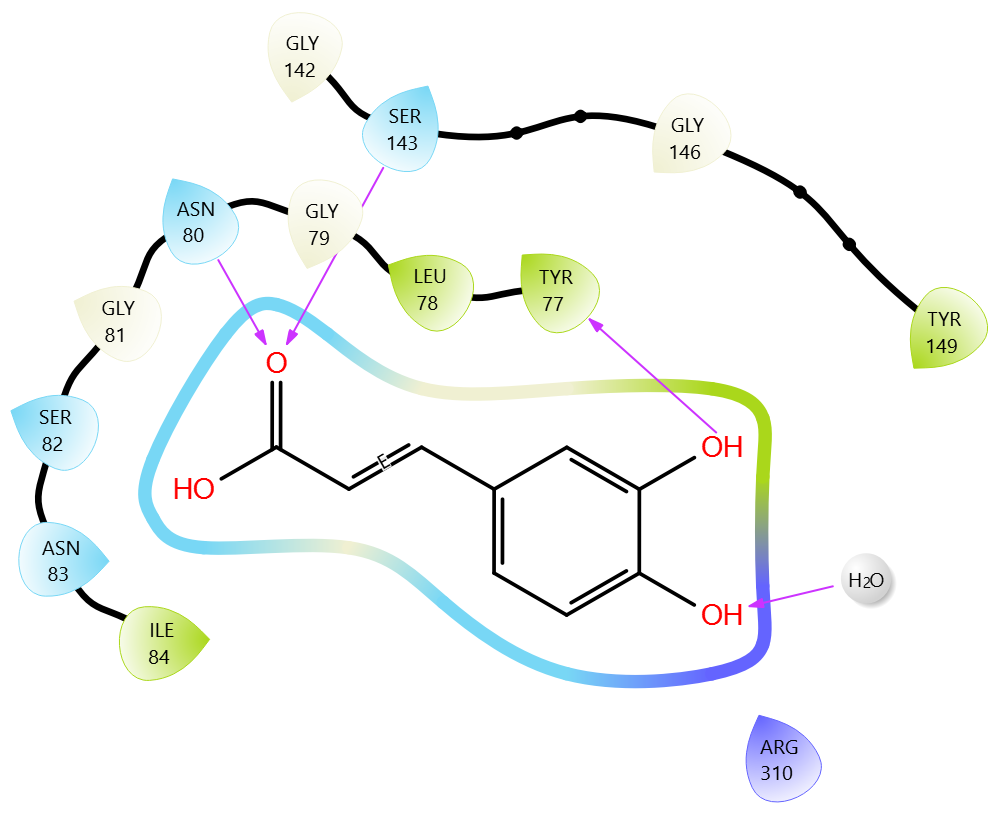
**

**Figure 43S.** 2D interaction diagram with 1HSK for caffeic acid.


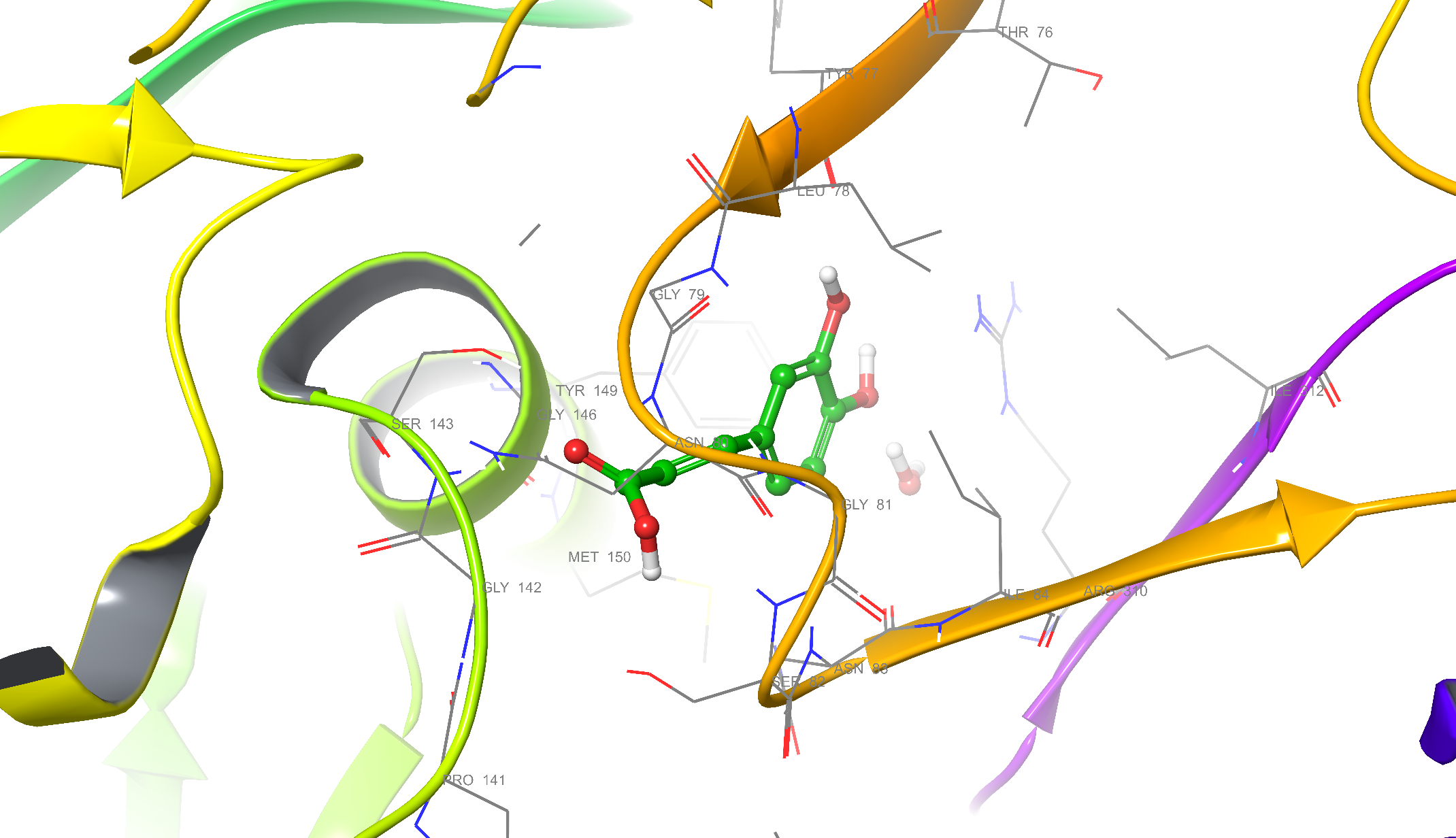


**Figure 44S.** 3D interaction diagram with 1HSK for caffeic acid.

**
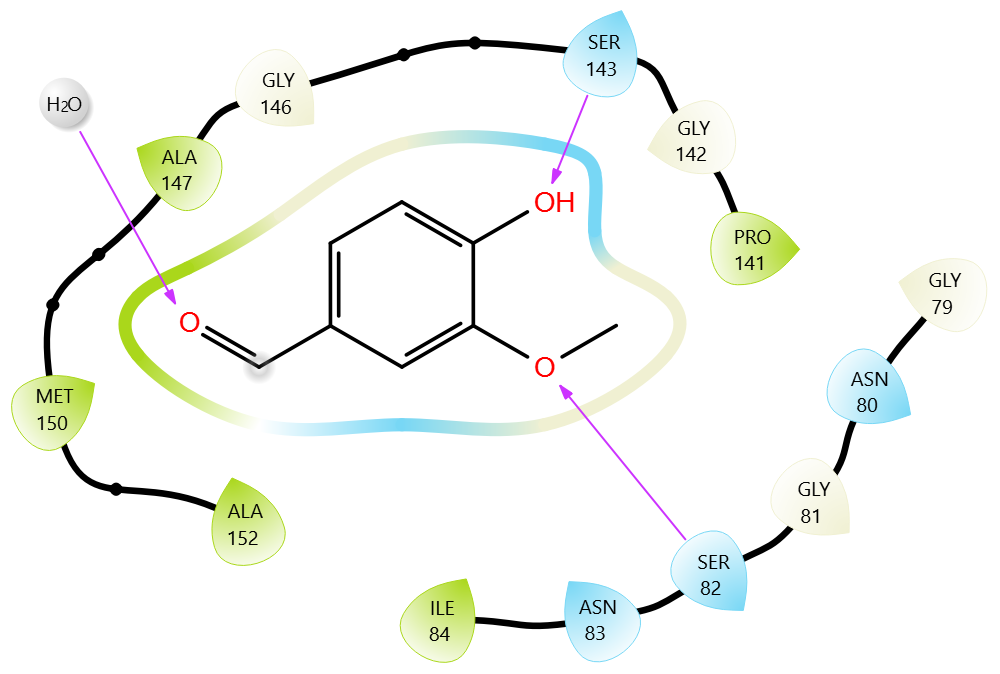
**

**Figure 45S.** 2D interaction diagram with 1HSK for vanillin.


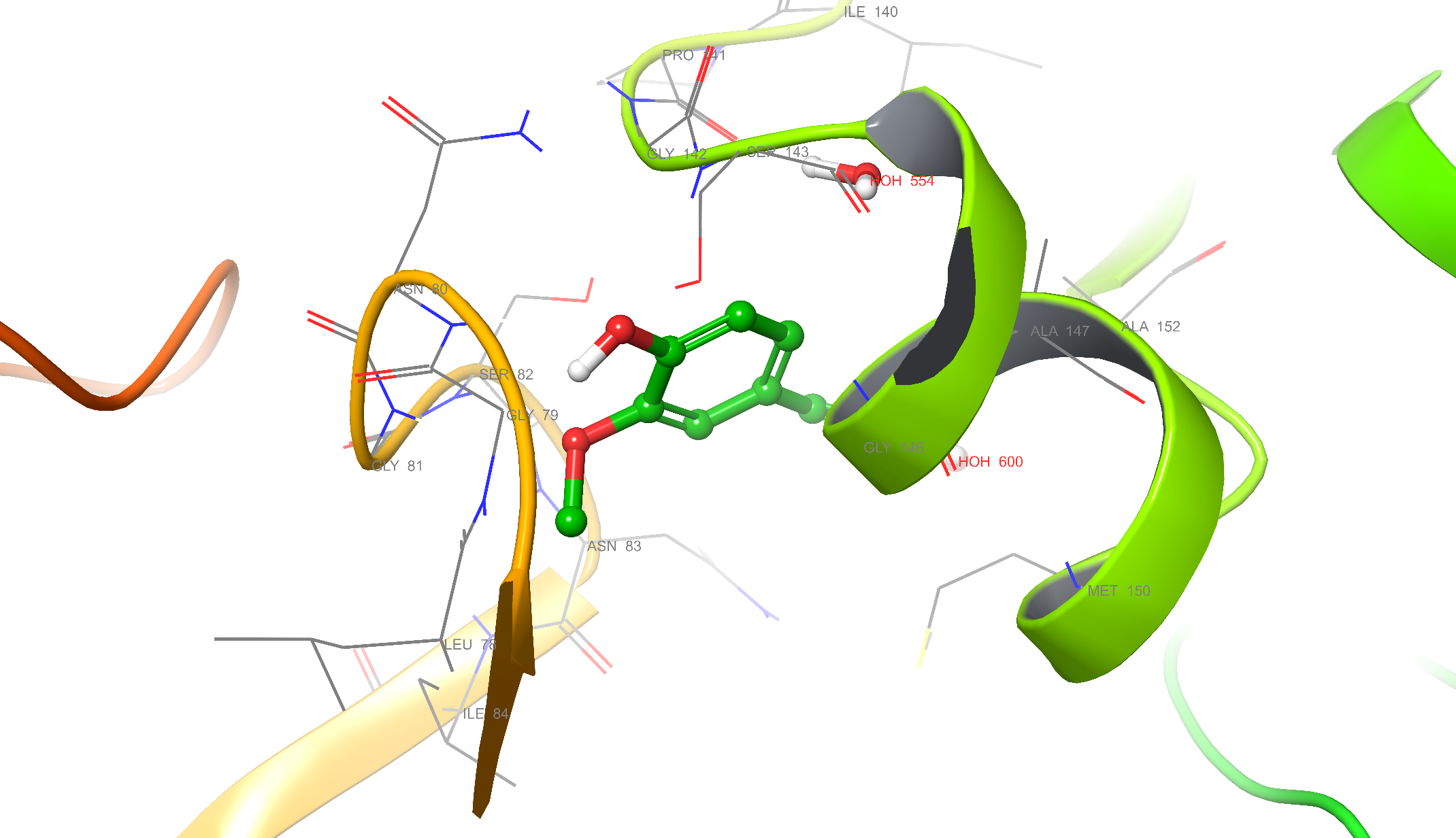


**Figure 46S.** 3D interaction diagram with 1HSK for vanillin.

**
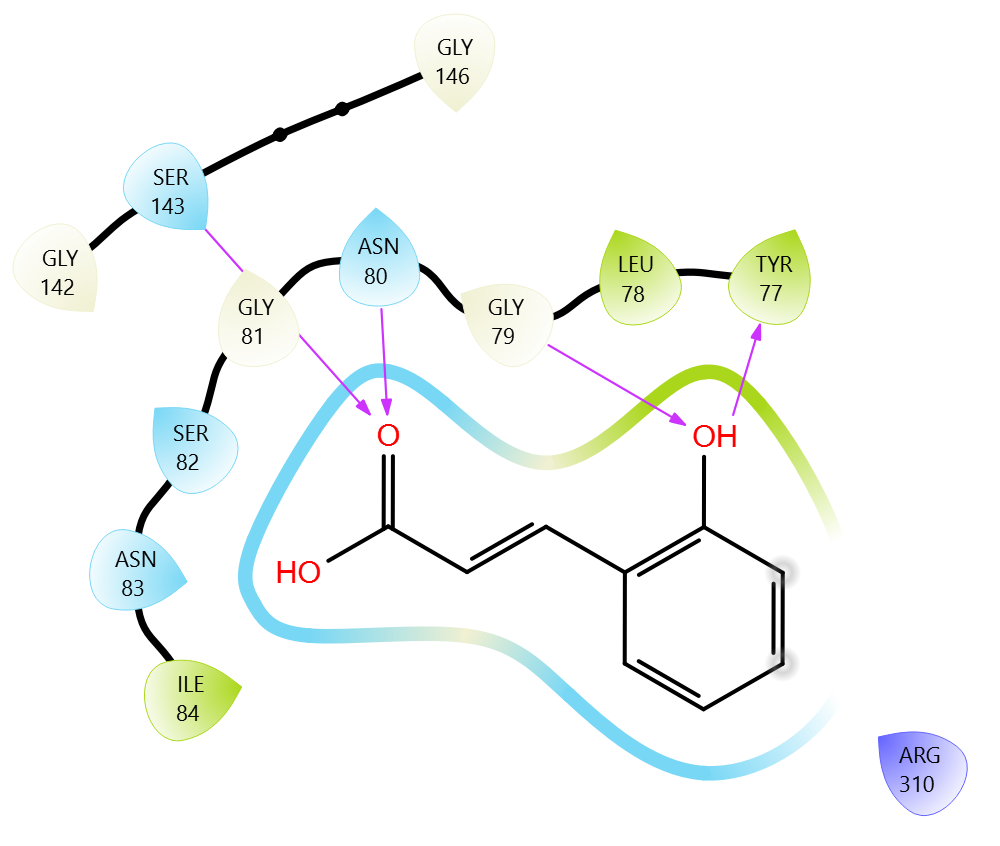
**

**Figure 47S.** 2D interaction diagram with 1HSK for *o*-coumaric acid.


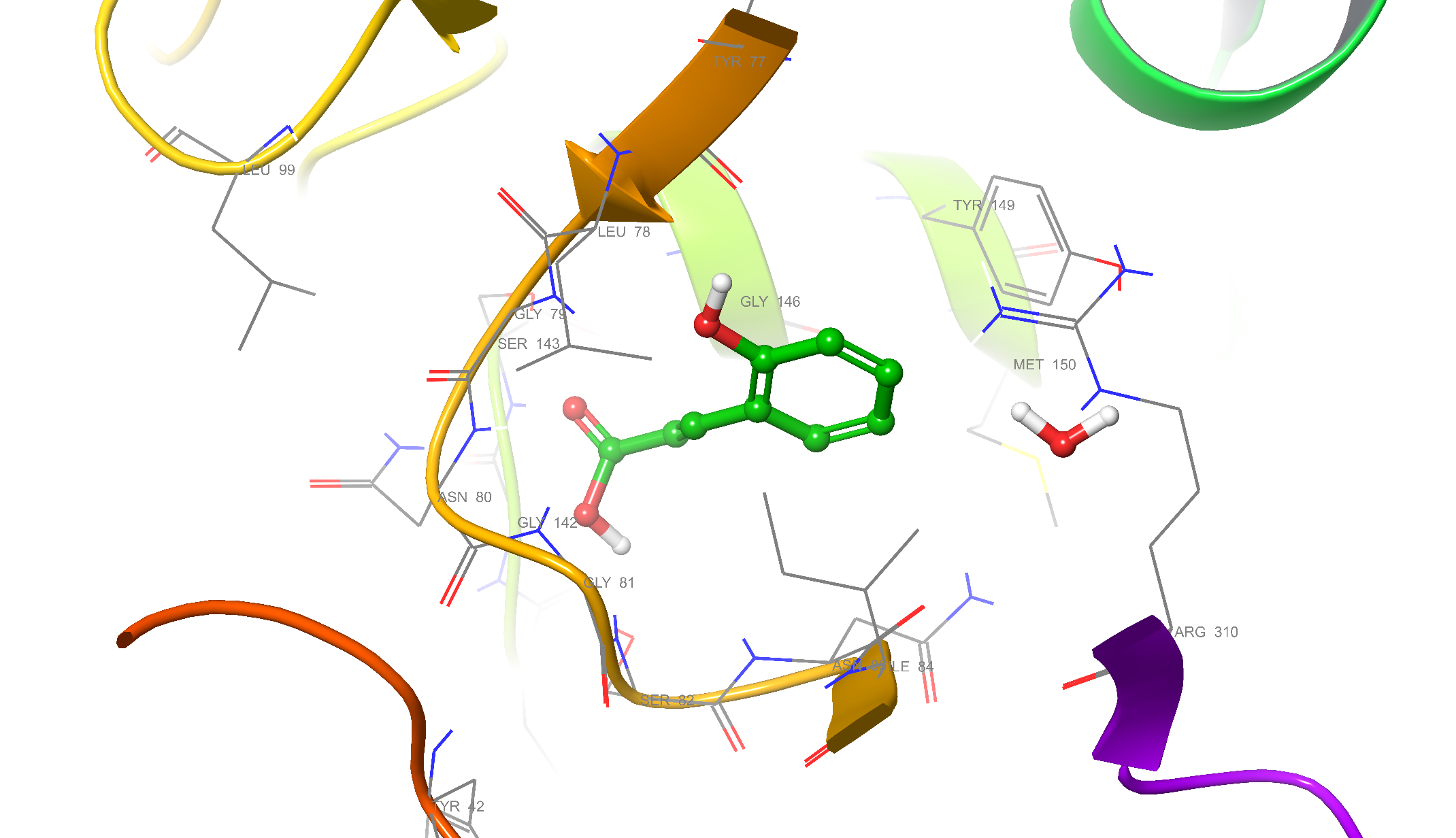


**Figure 48S.** 3D interaction diagram with 1HSK for *o*-coumaric acid.

**
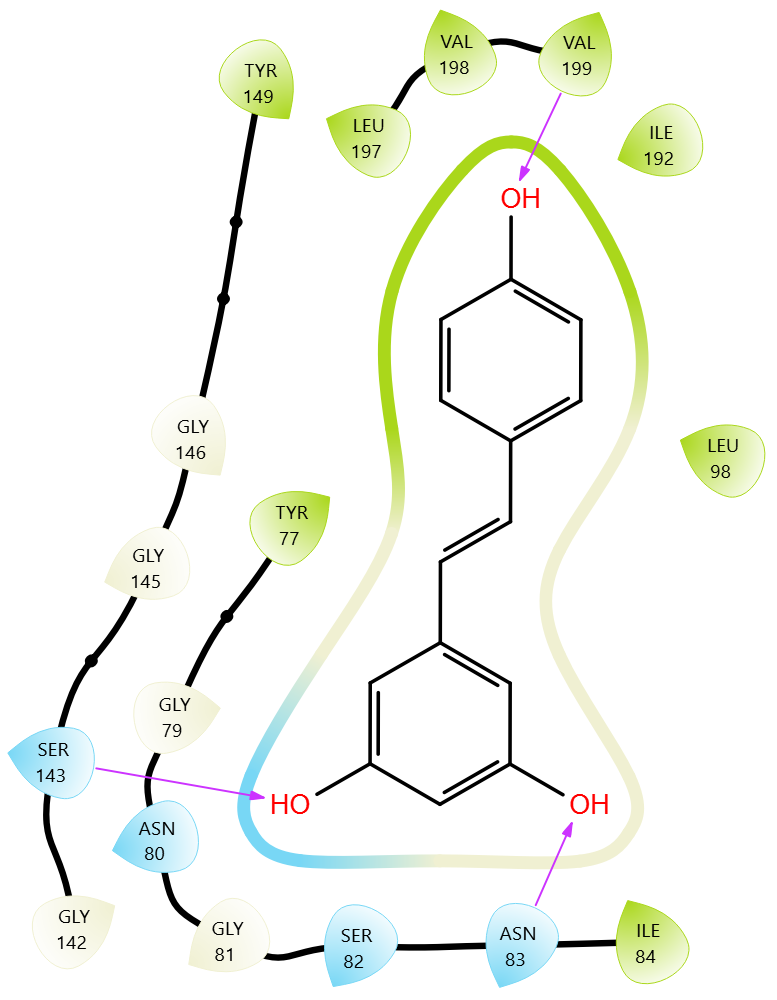
**

**Figure 49S.** 2D interaction diagram with 1HSK for resveratrol.


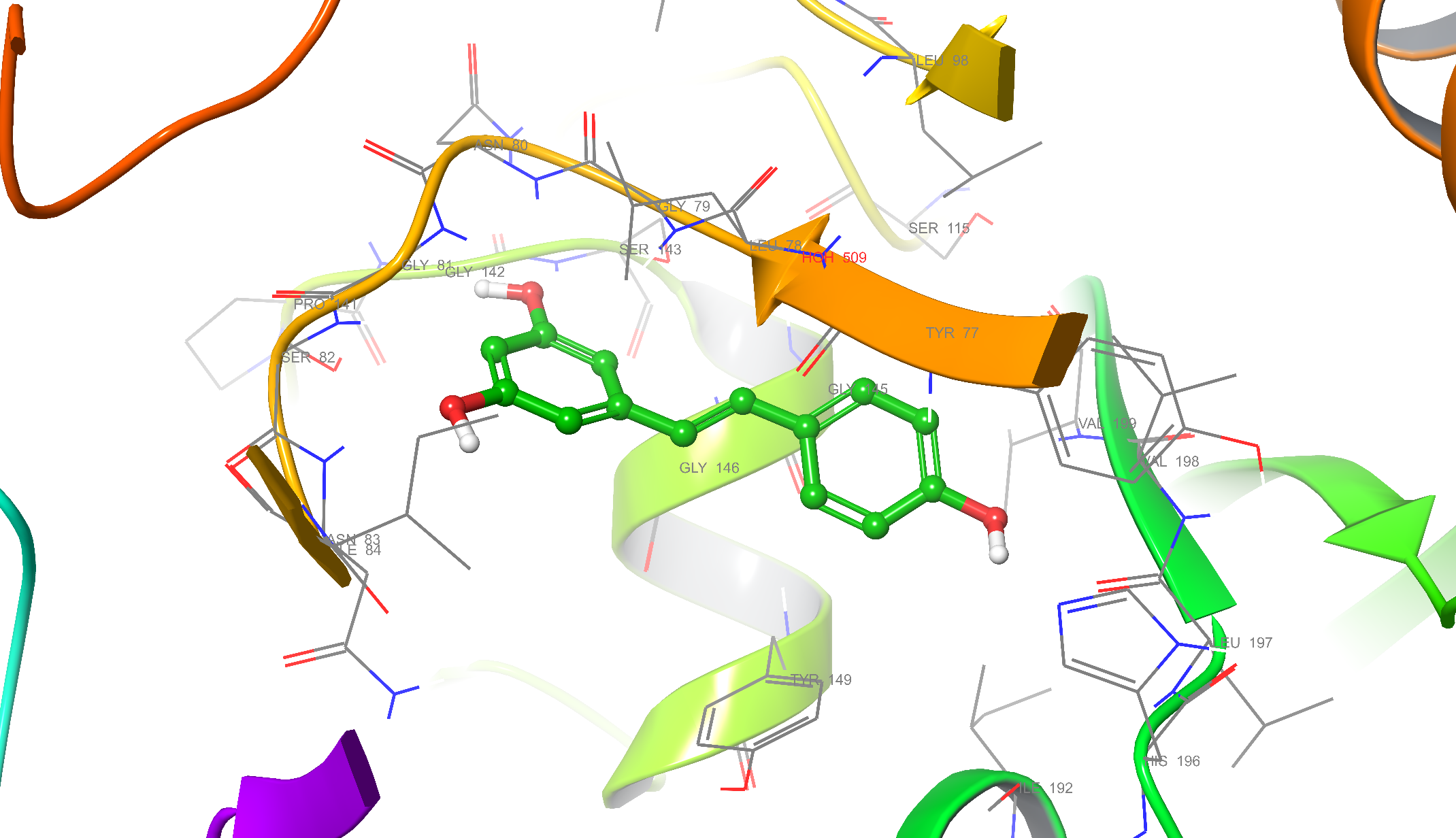


**Figure 50S.** 3D interaction diagram with 1HSK for resveratrol.

**
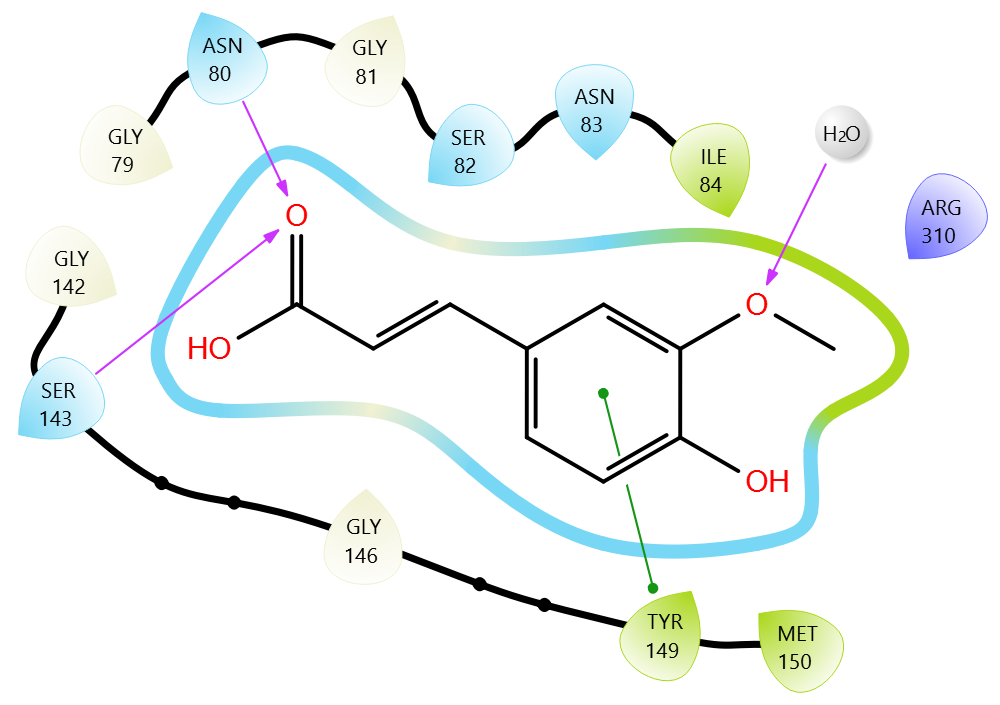
**

**Figure 51S.** 2D interaction diagram with 1HSK for *trans*-ferulic acid.


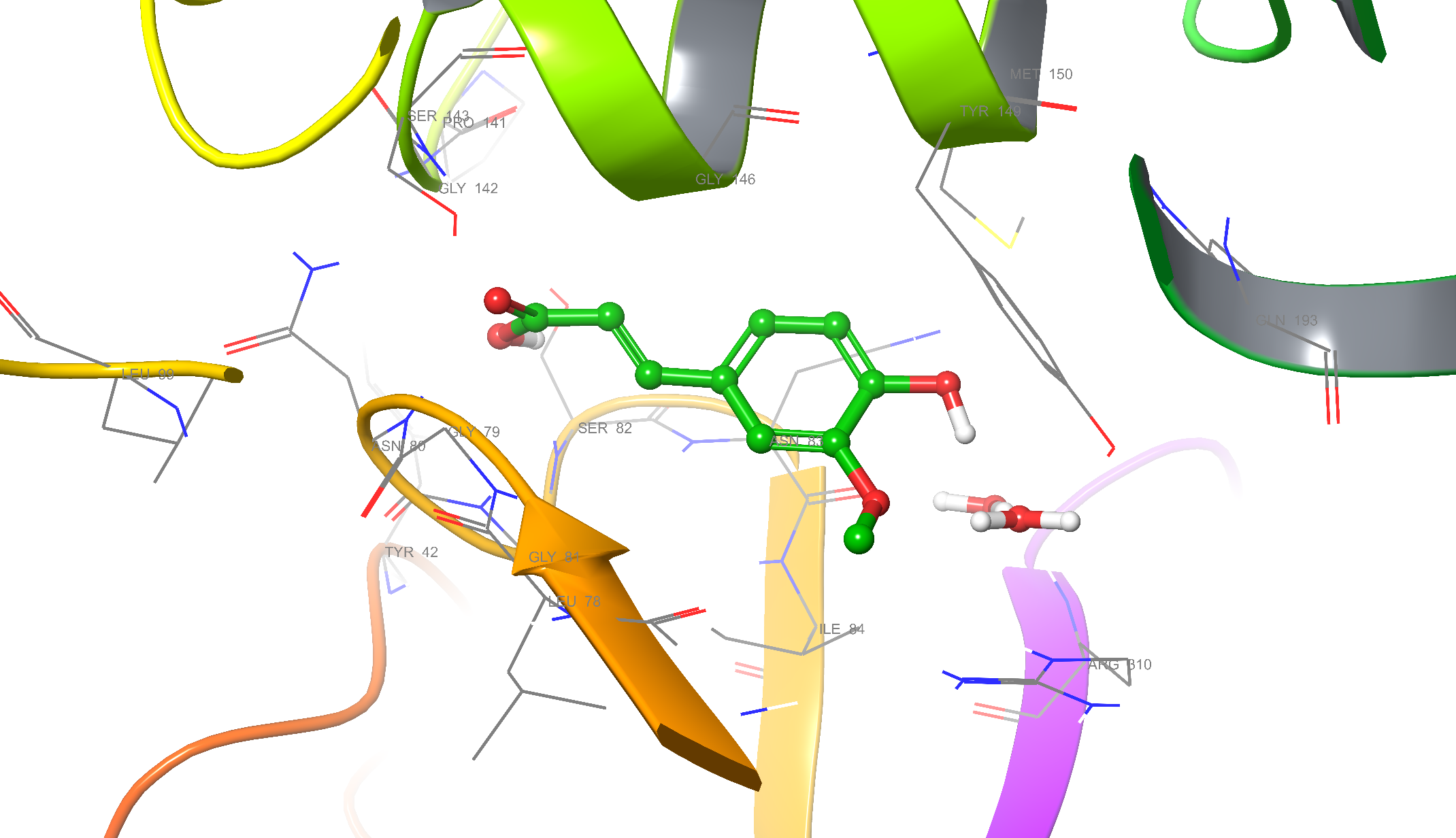


**Figure 52S.** 3D interaction diagram with 1HSK for *trans*-ferulic acid.

**
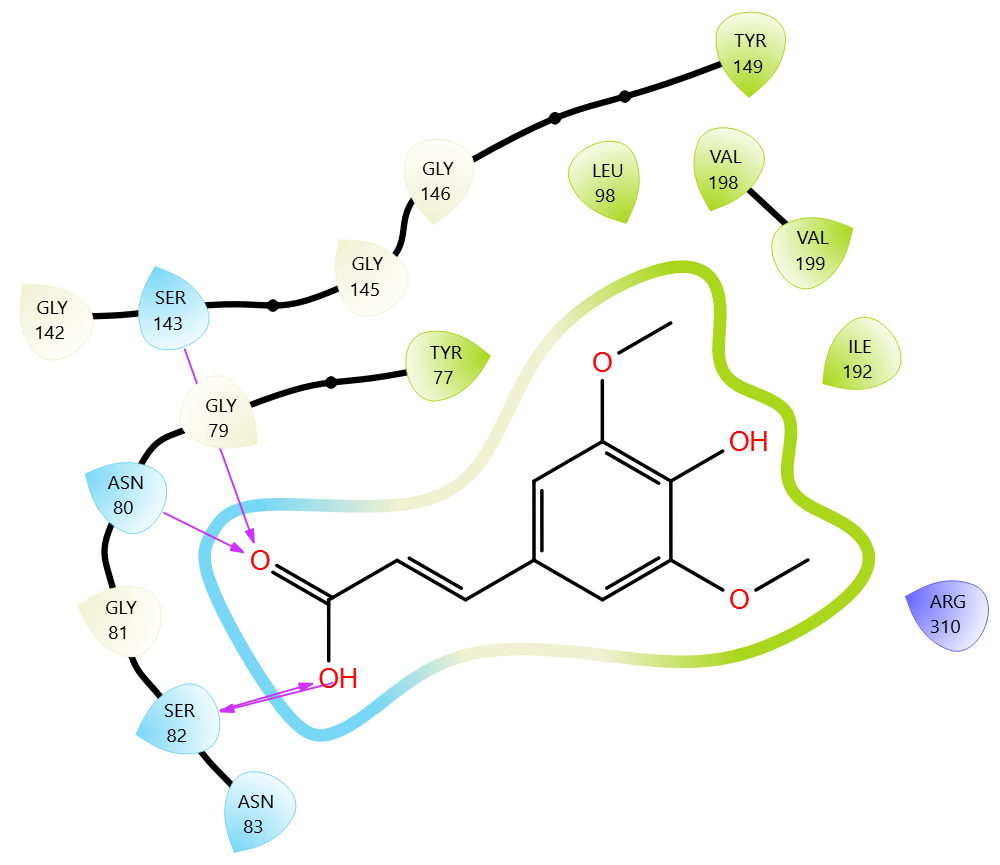
**

**Figure 53S.** 2D interaction diagram with 1HSK for sinapic acid.

**
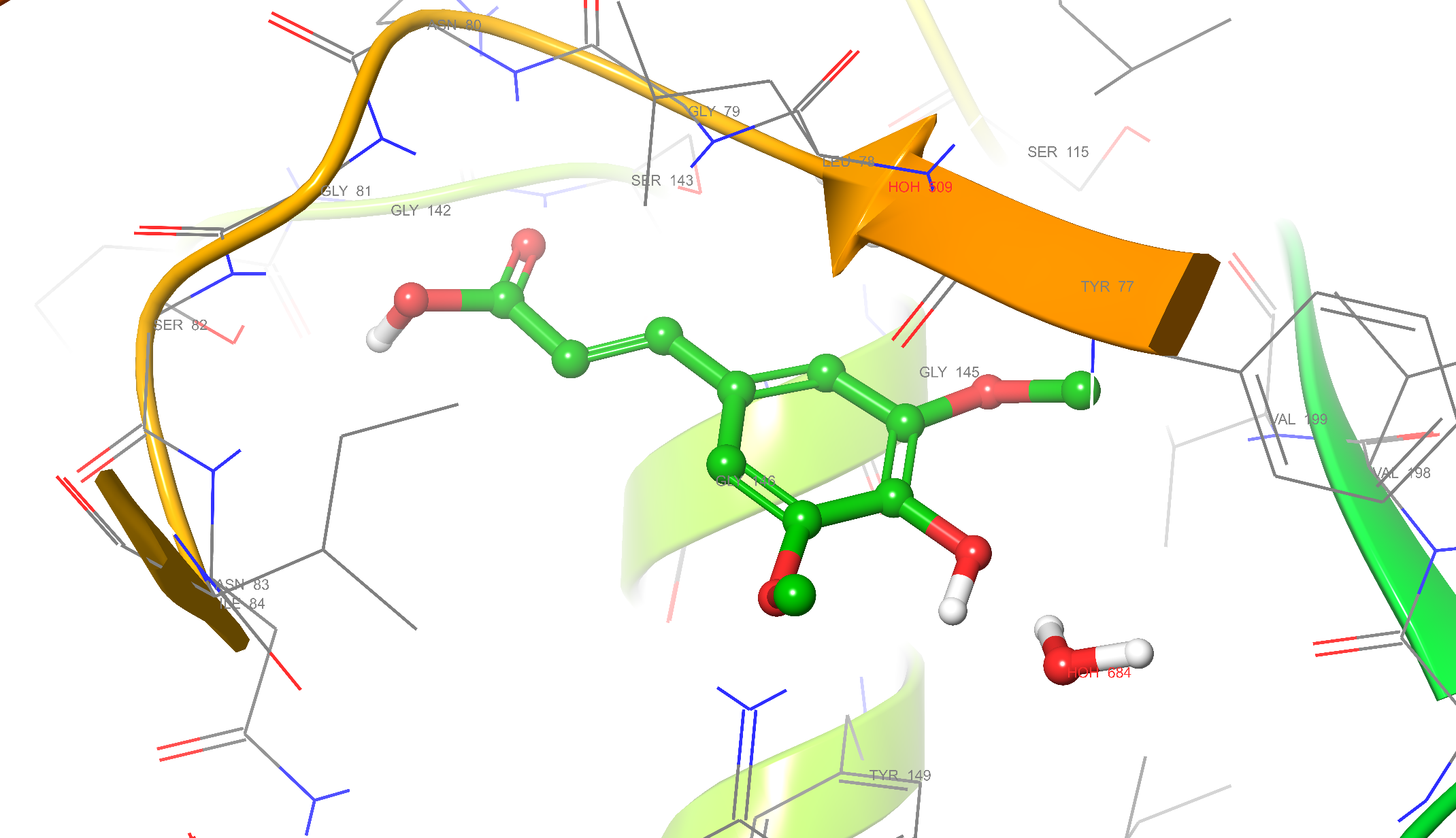
**

**Figure 54S.** 3D interaction diagram with 1HSK for sinapic acid.

**
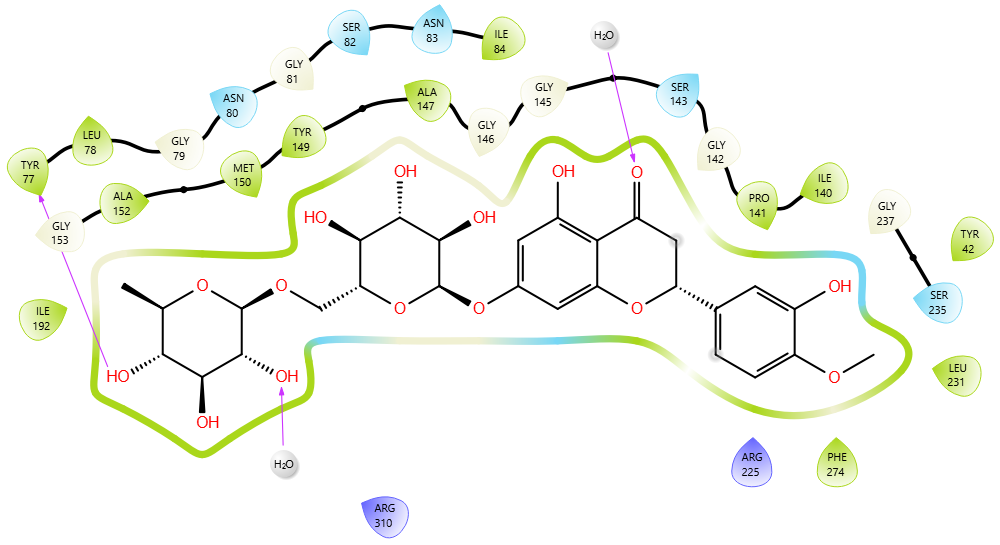
**

**Figure 55S.** 2D interaction diagram with 1HSK for hesperidin.

**
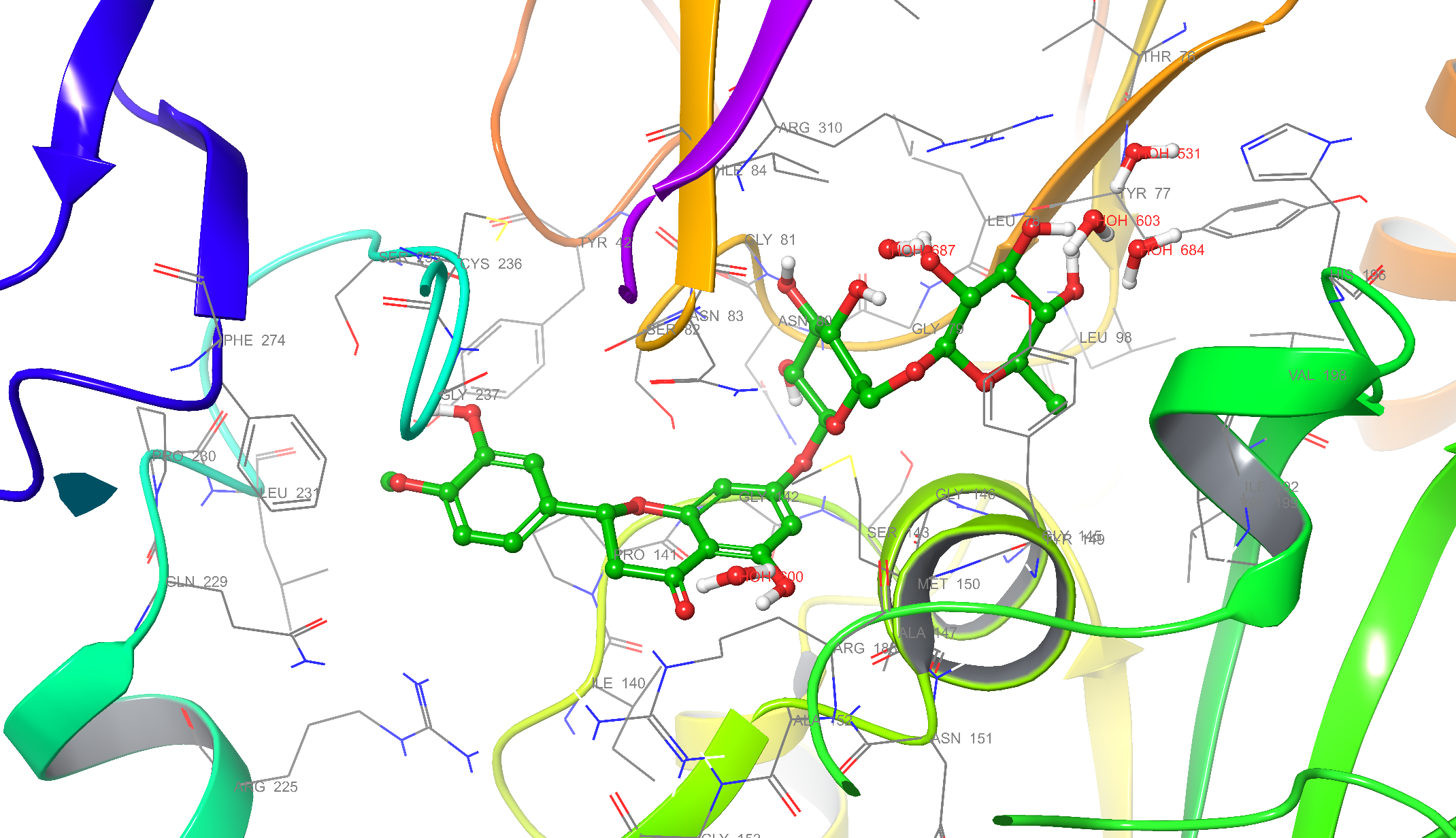
**

**Figure 56S.** 3D interaction diagram with 1HSK for hesperidin.

**
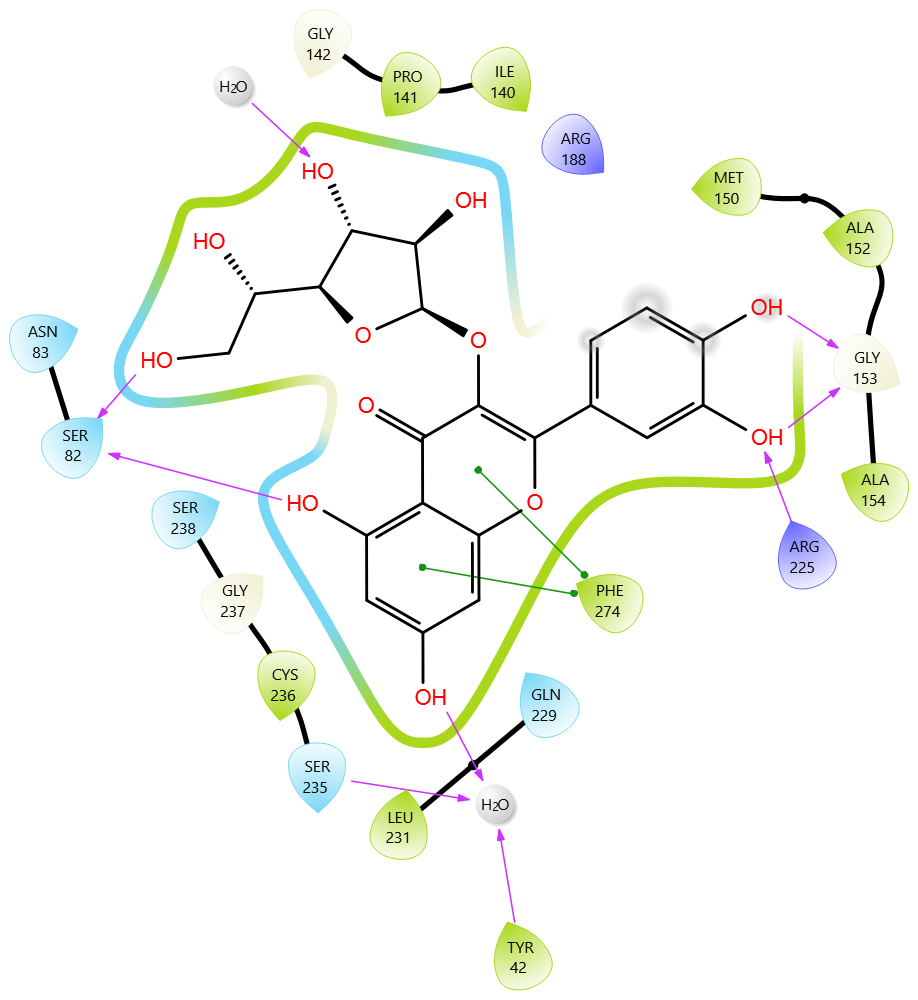
**

**Figure 57S.** 2D interaction diagram with 1HSK for isoquercitrin.


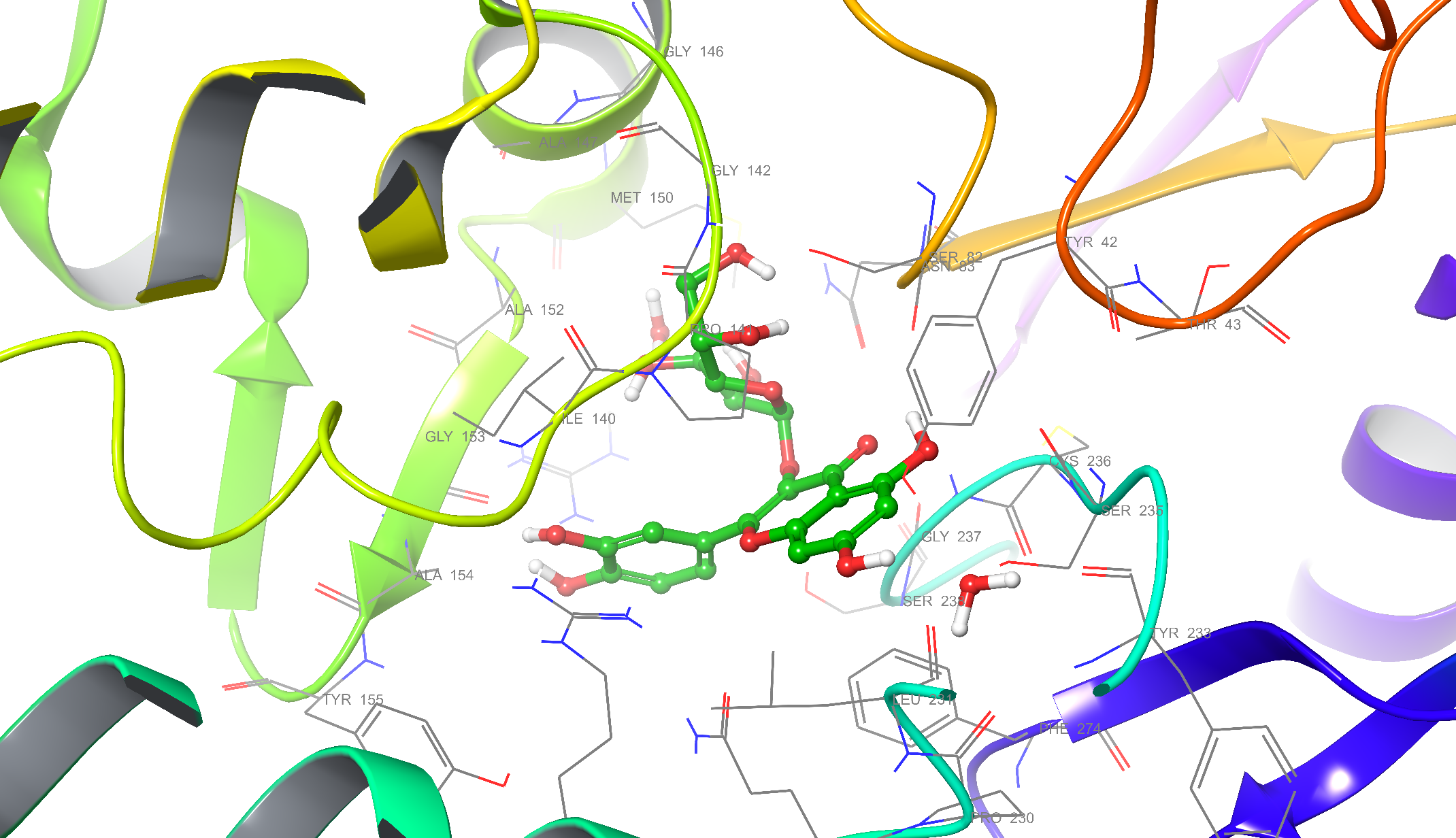


**Figure 58S.** 3D interaction diagram with 1HSK for isoquercitrin.

**
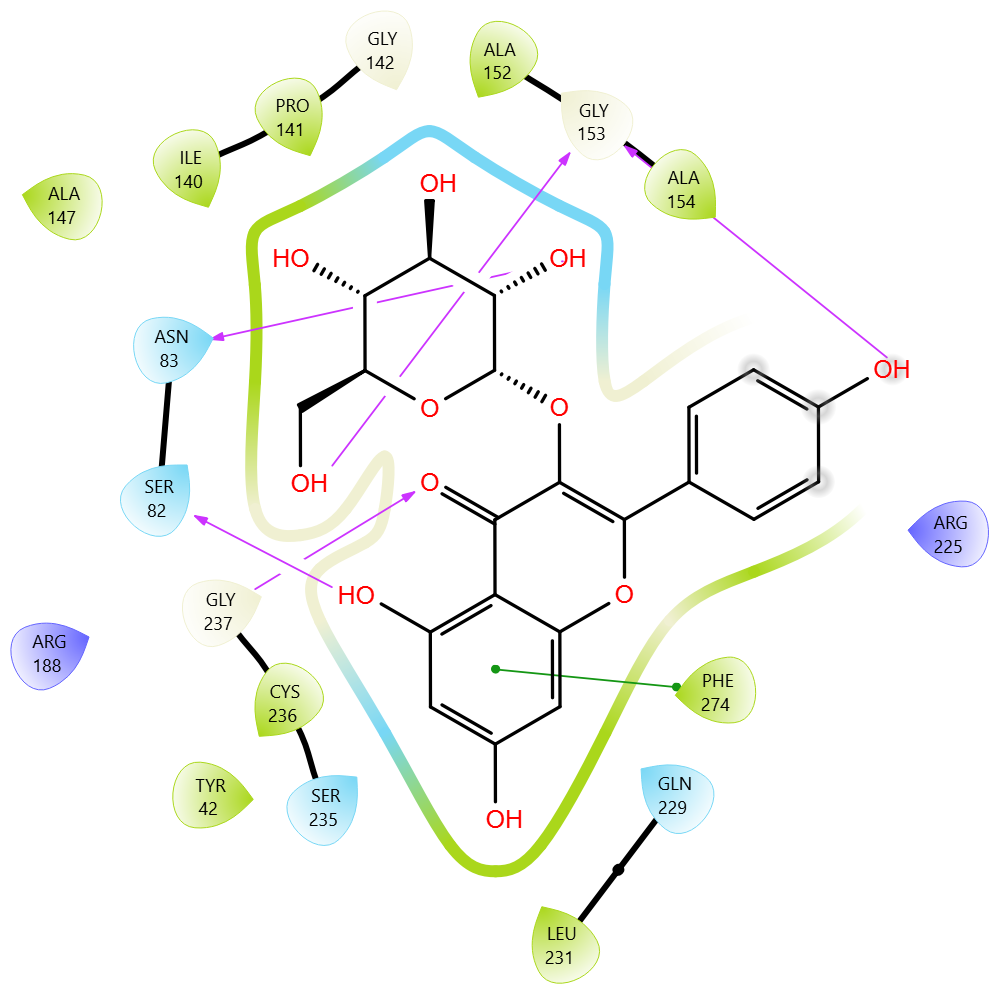
**

**Figure 59S.** 2D interaction diagram with 1HSK for kaempferol-3-glucoside.


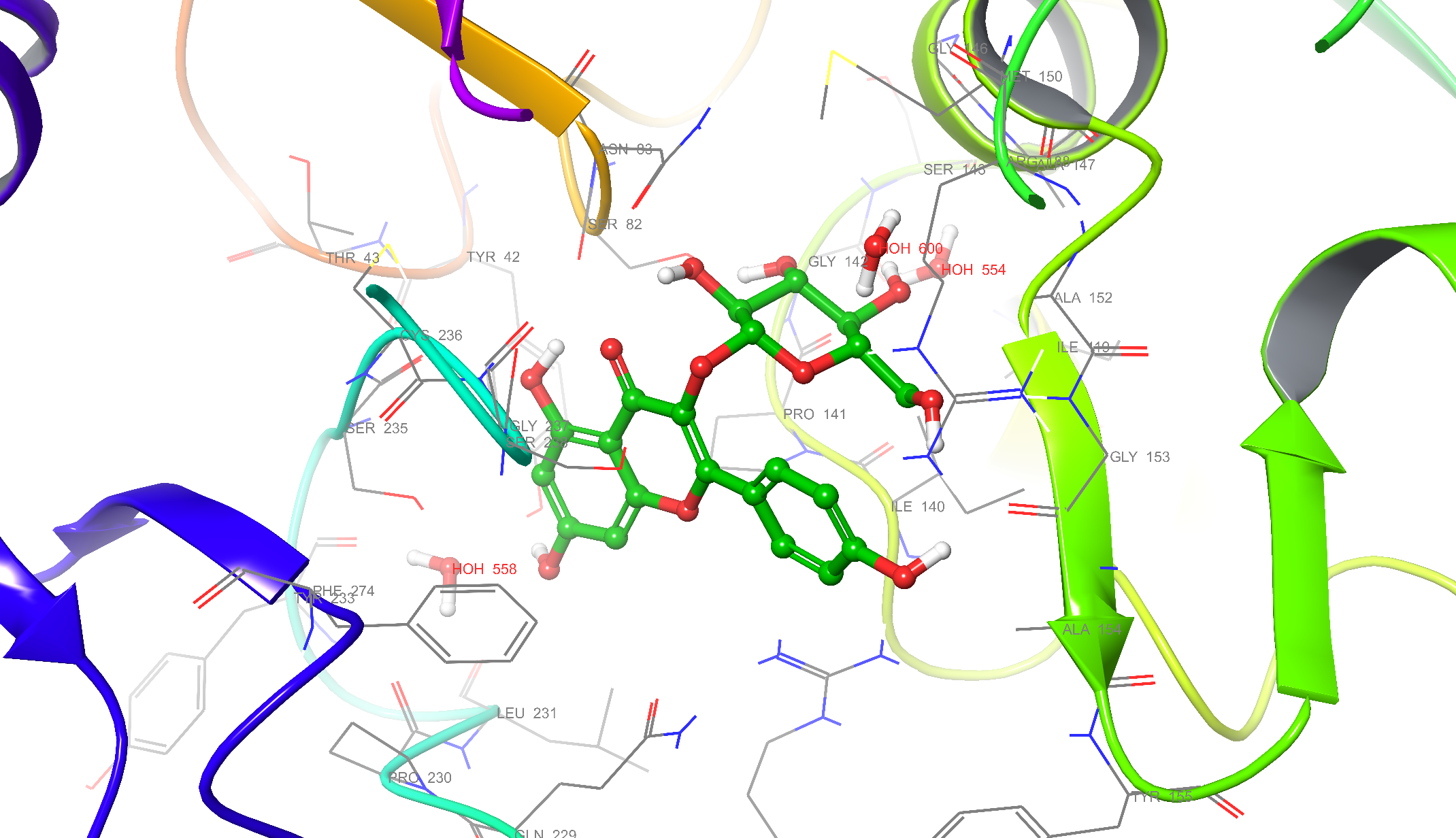


**Figure 60S.** 3D interaction diagram with 1HSK for kaempferol-3-glucoside.

**
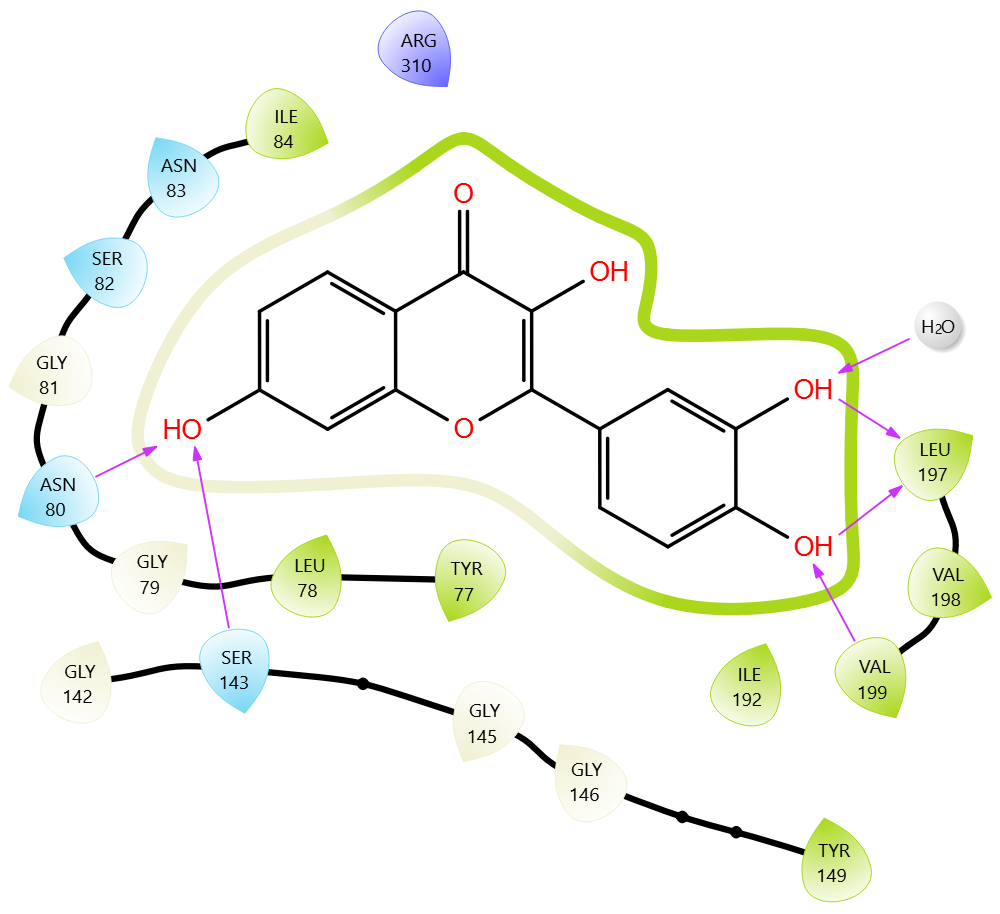
**

**Figure 61S.** 2D interaction diagram with 1HSK for fisetin.


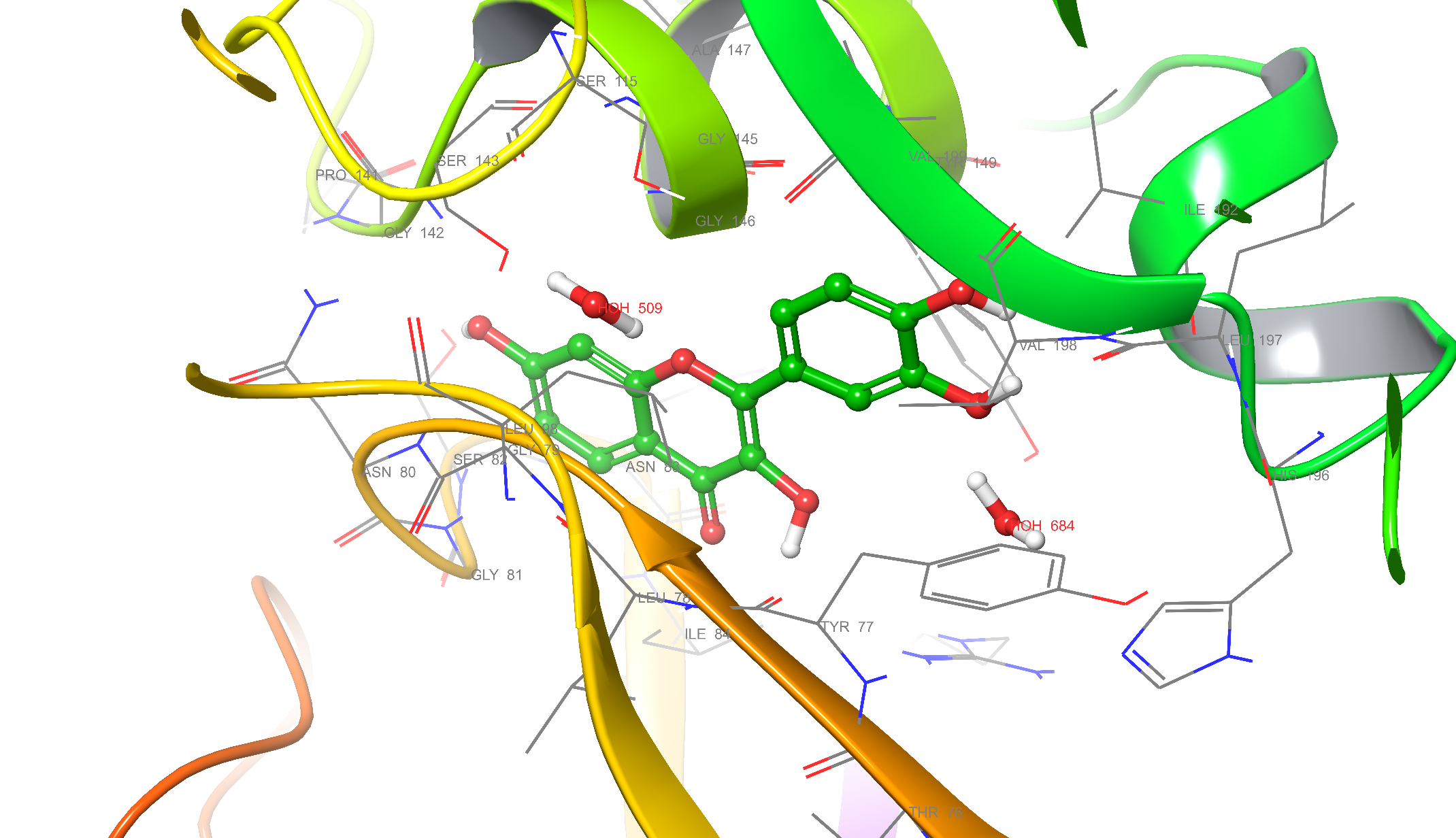


**Figure 62S.** 3D interaction diagram with 1HSK for fisetin.

**
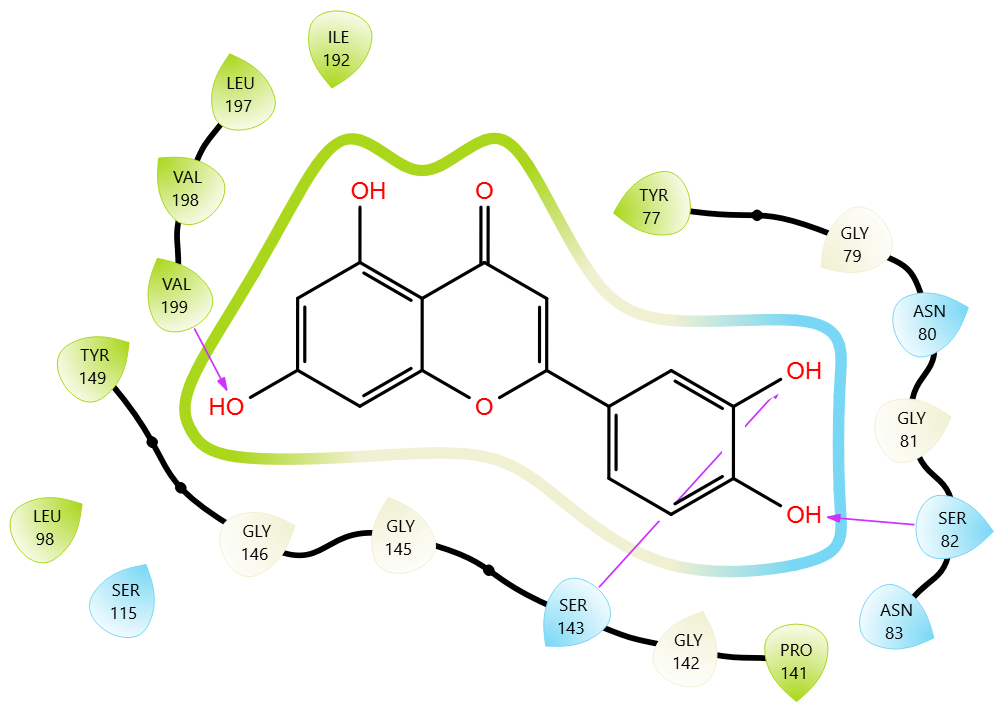
**

**Figure 63S.** 2D interaction diagram with 1HSK for luteolin.


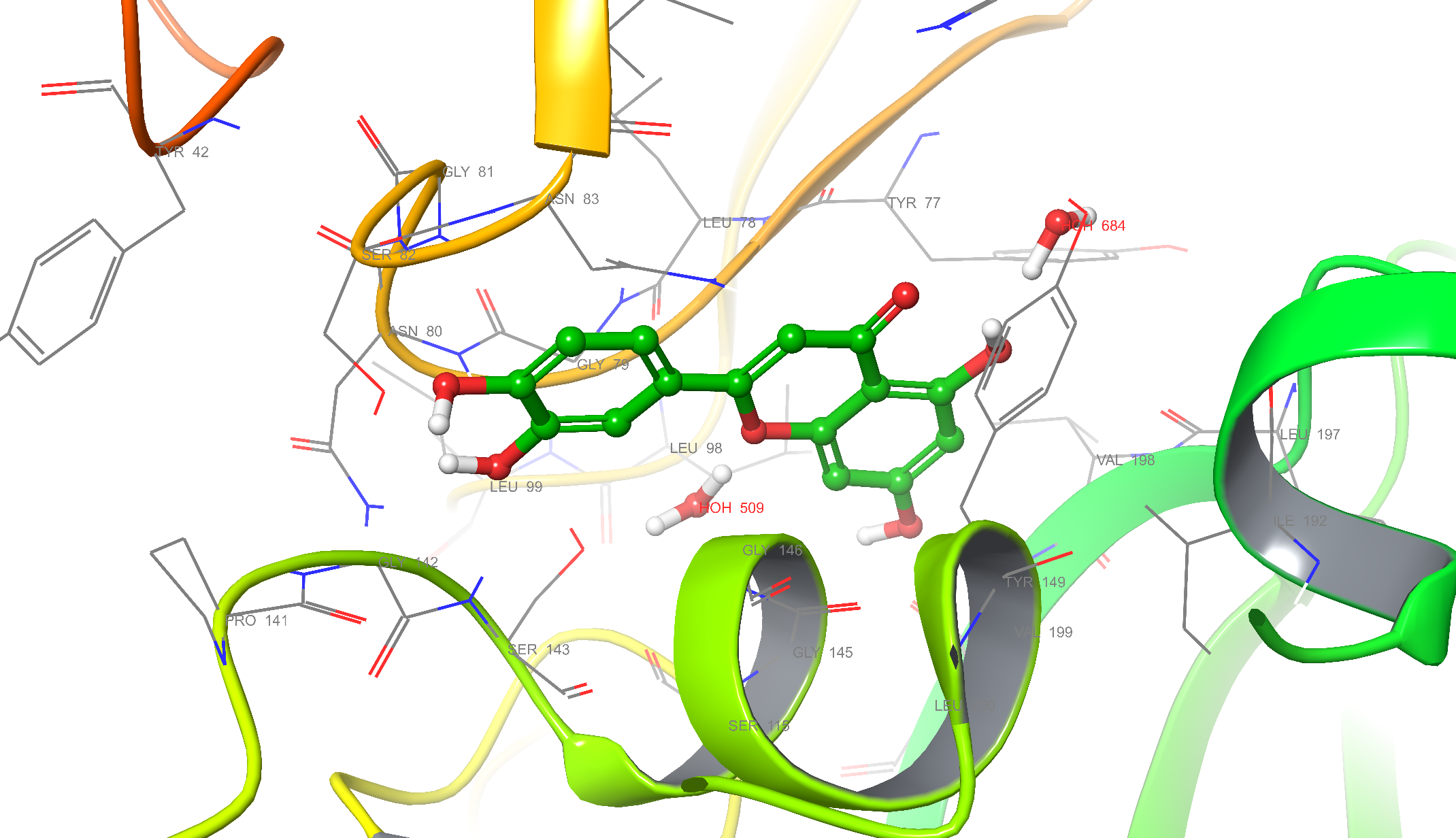


**Figure 64S.** 3D interaction diagram with 1HSK for luteolin.
